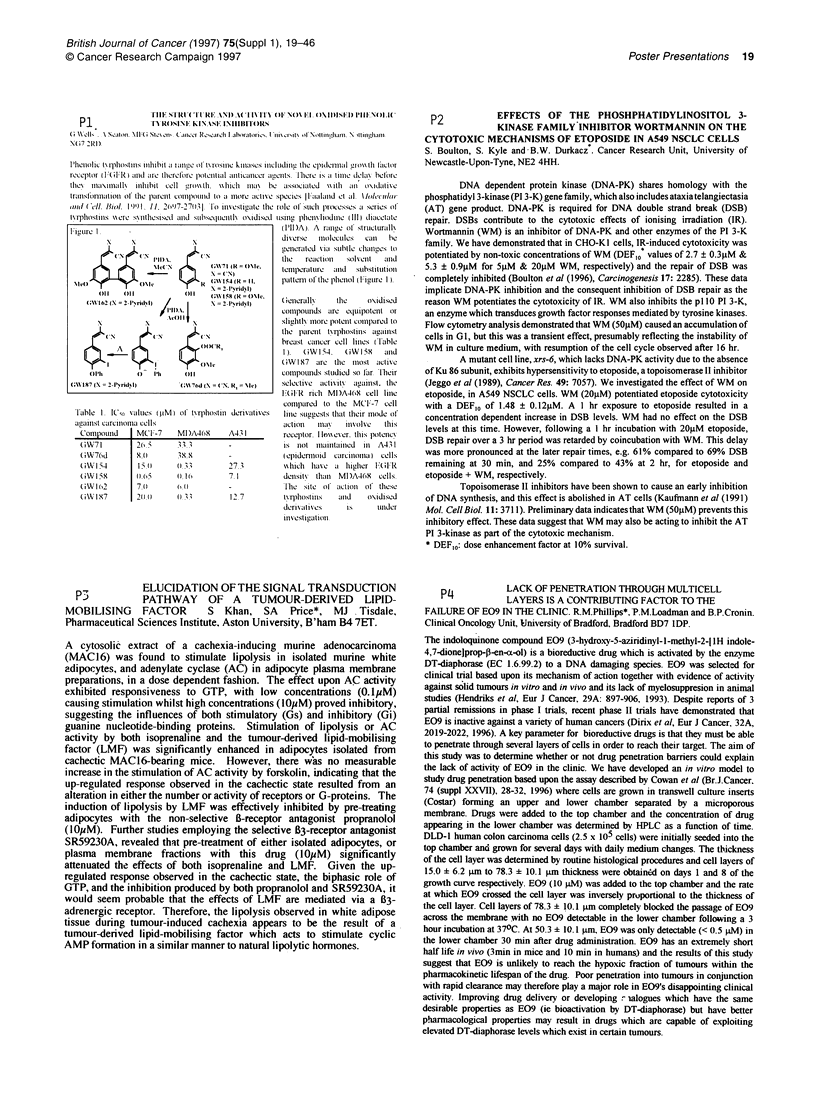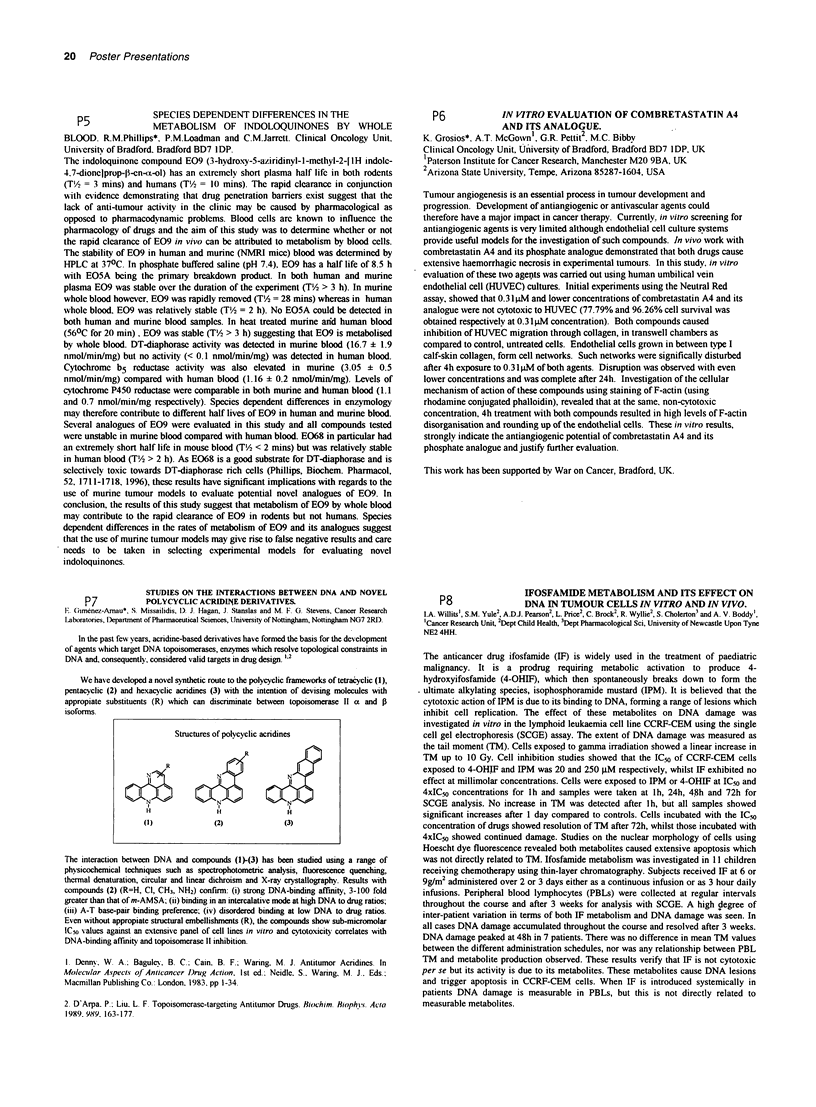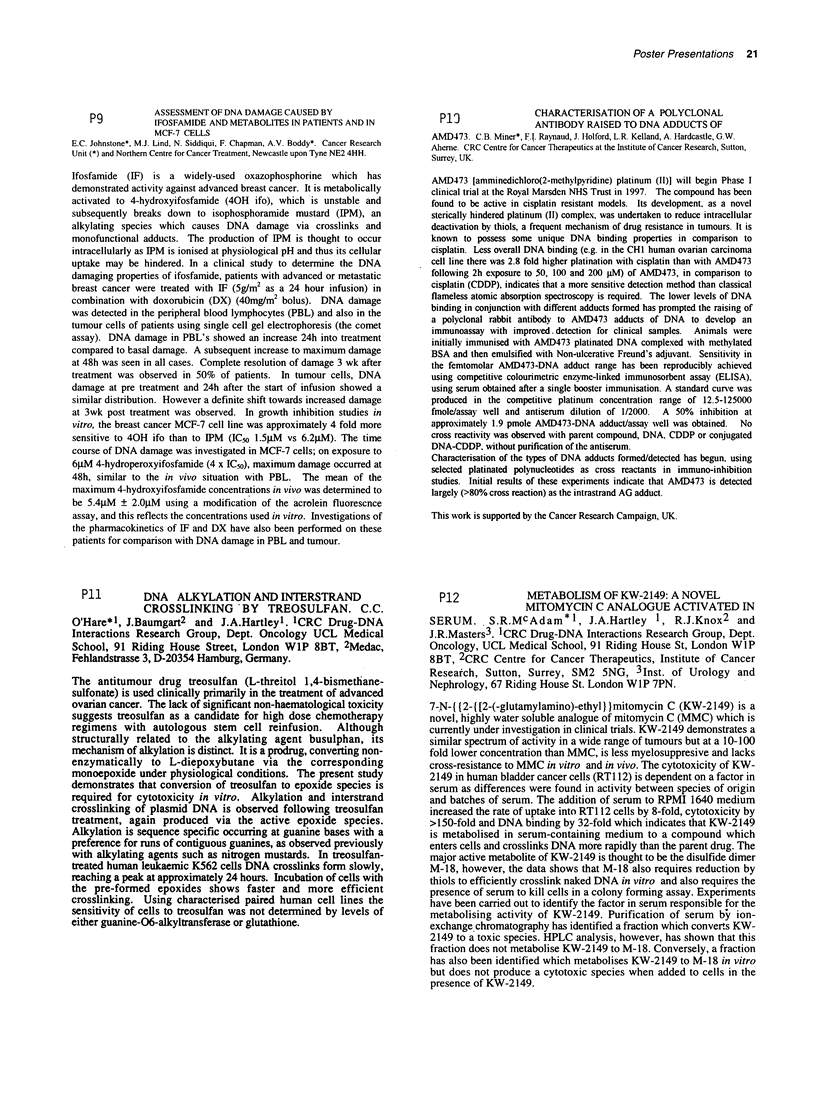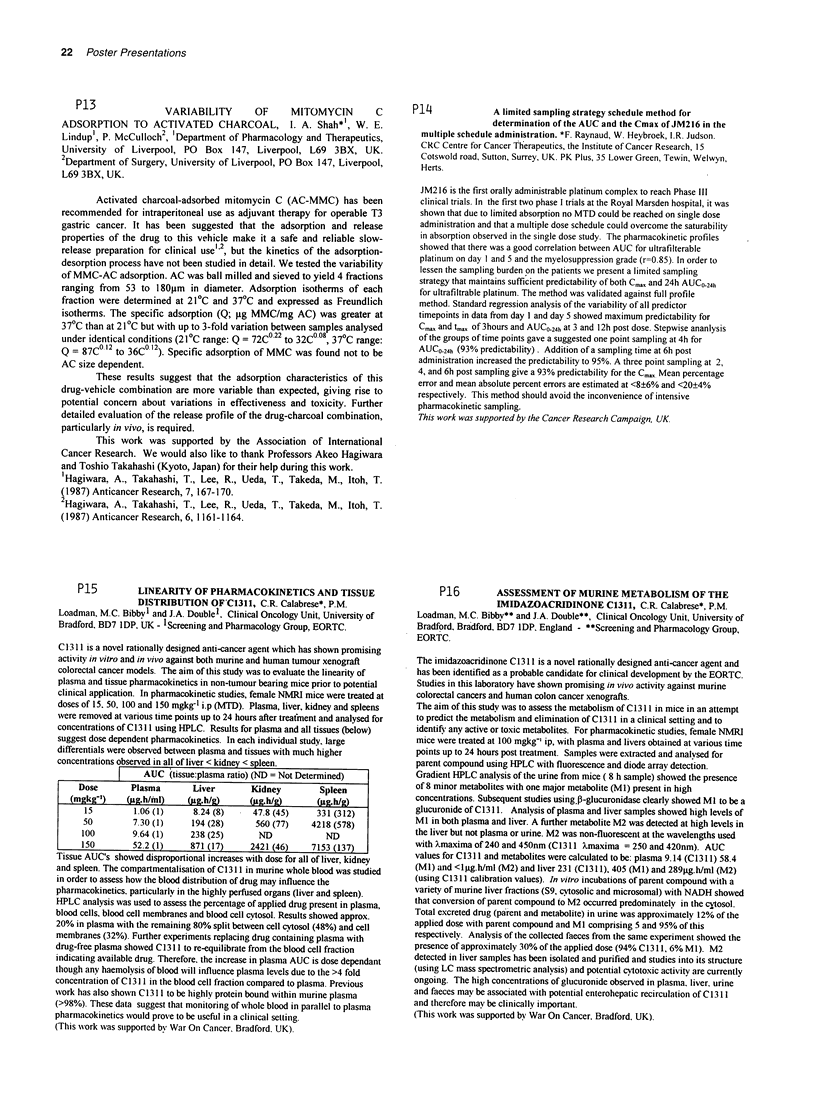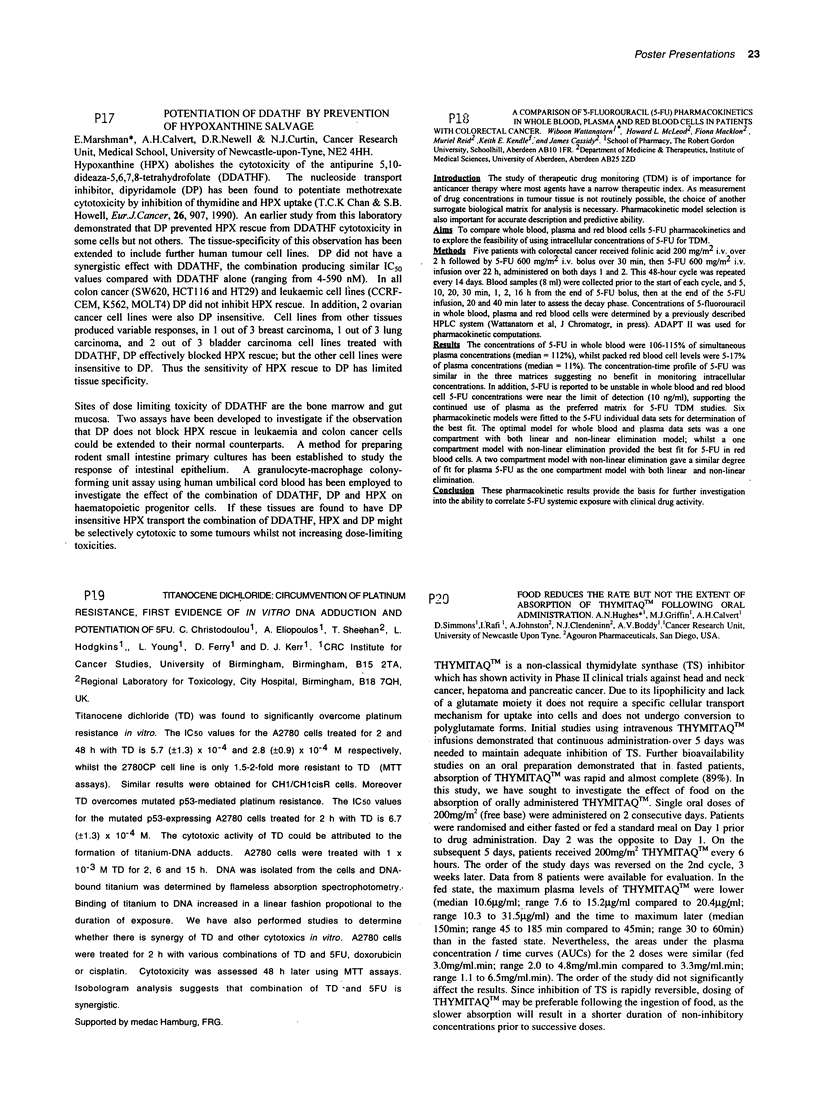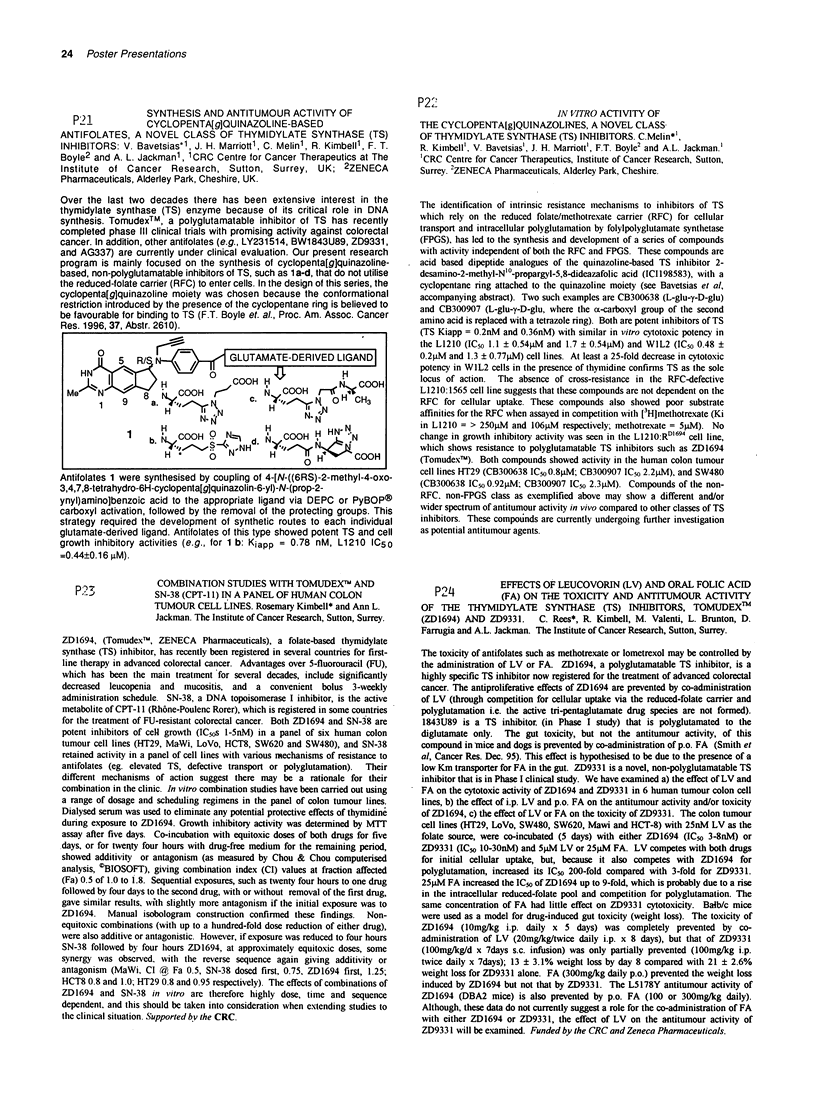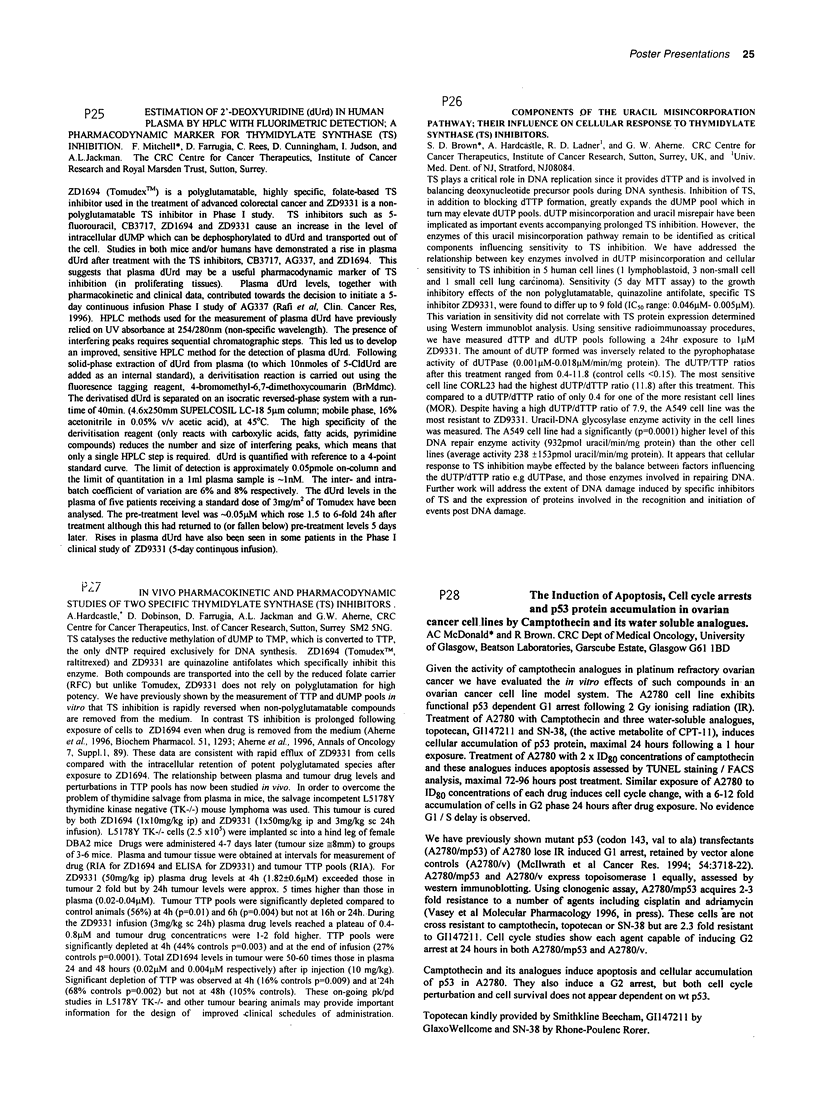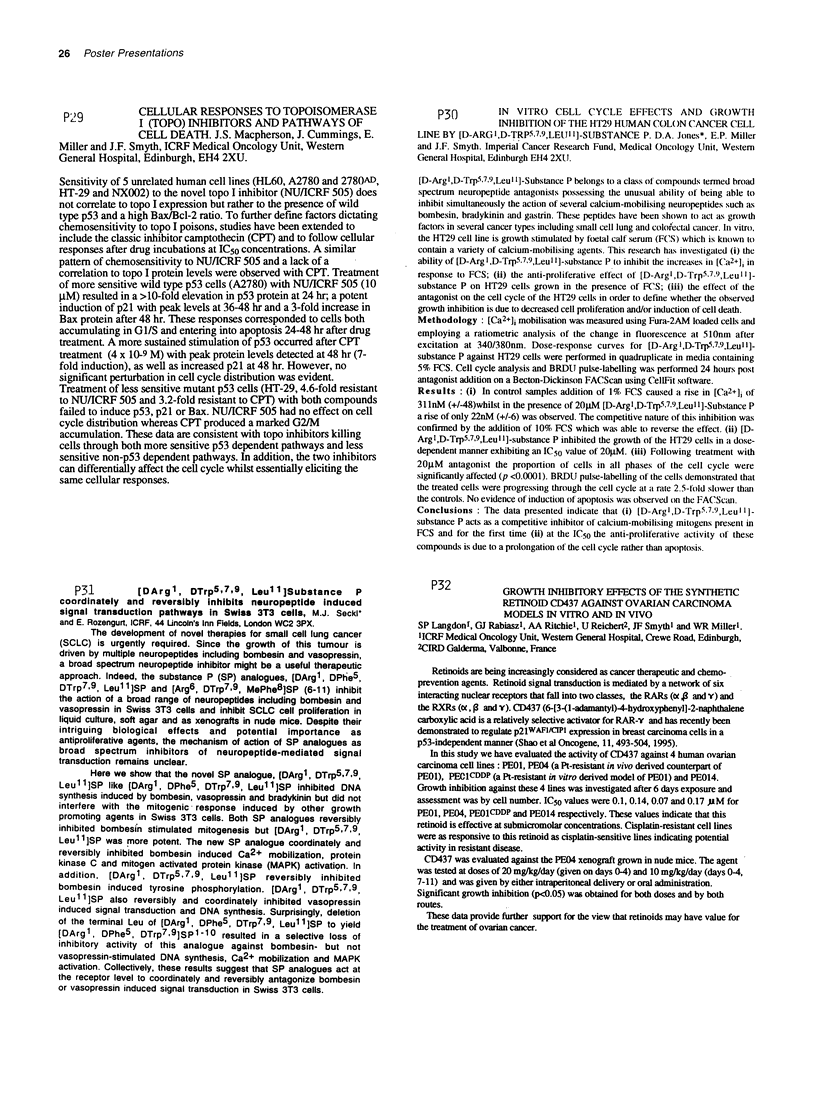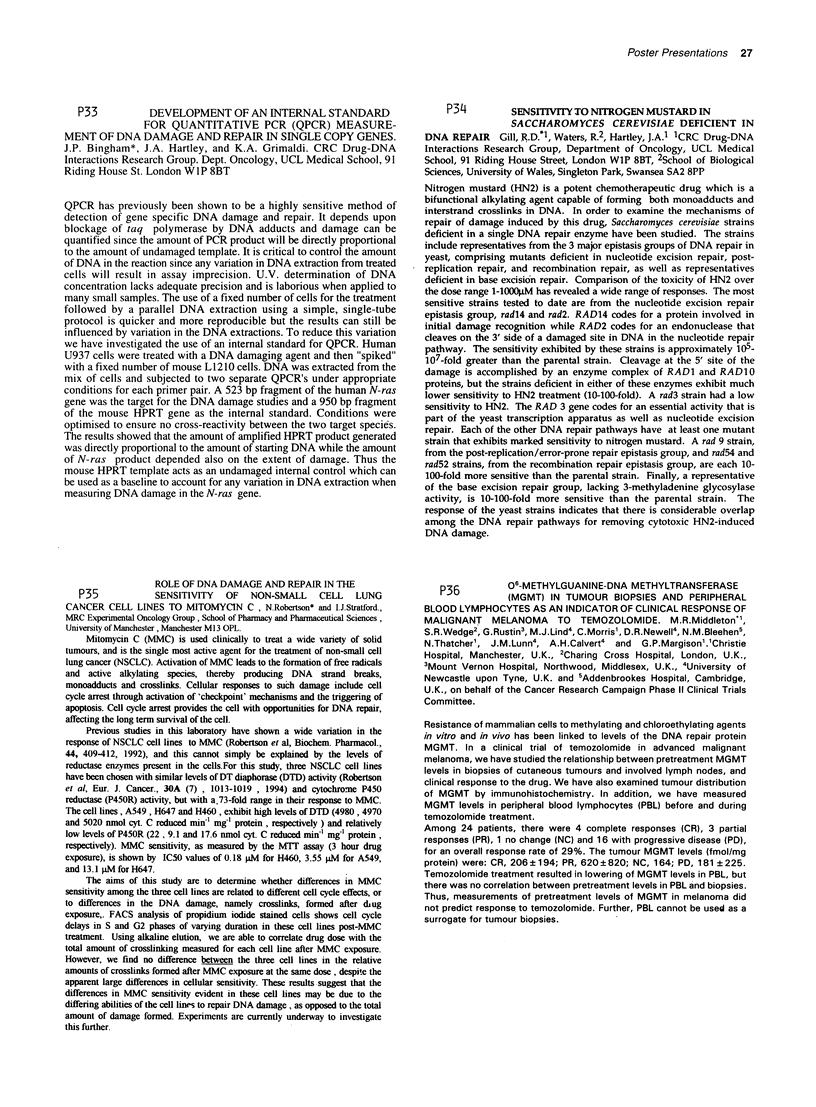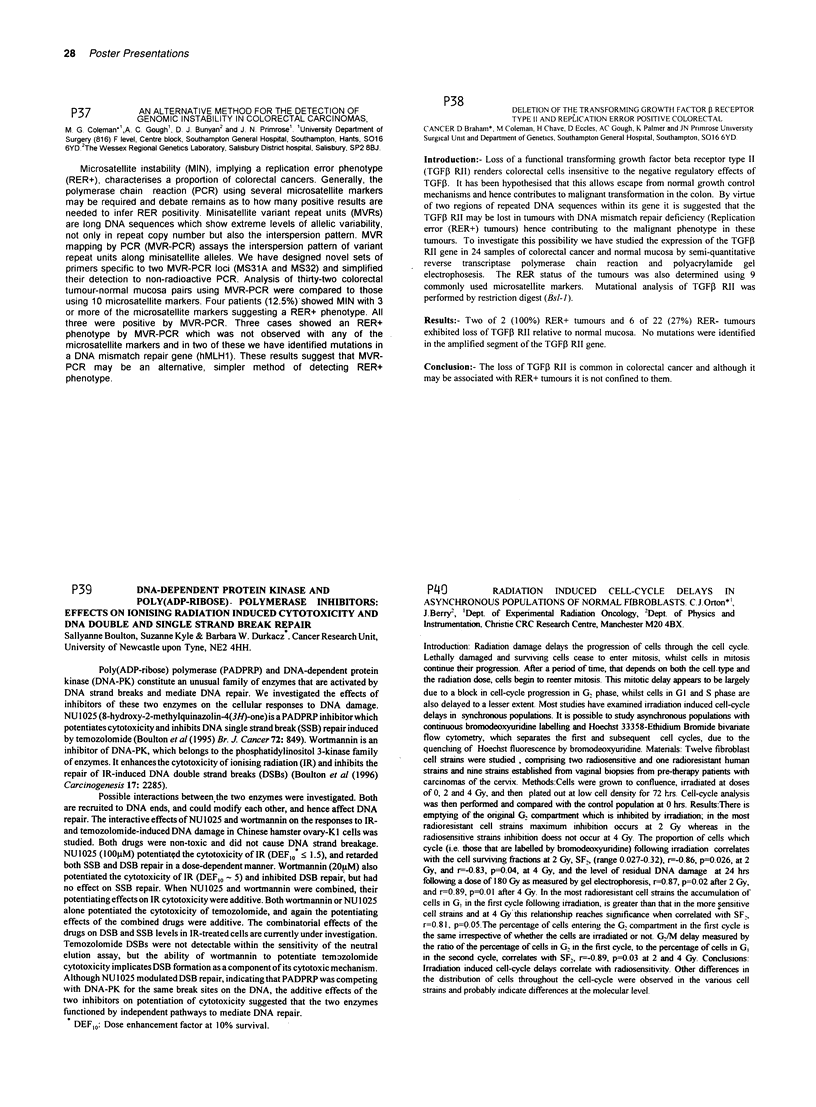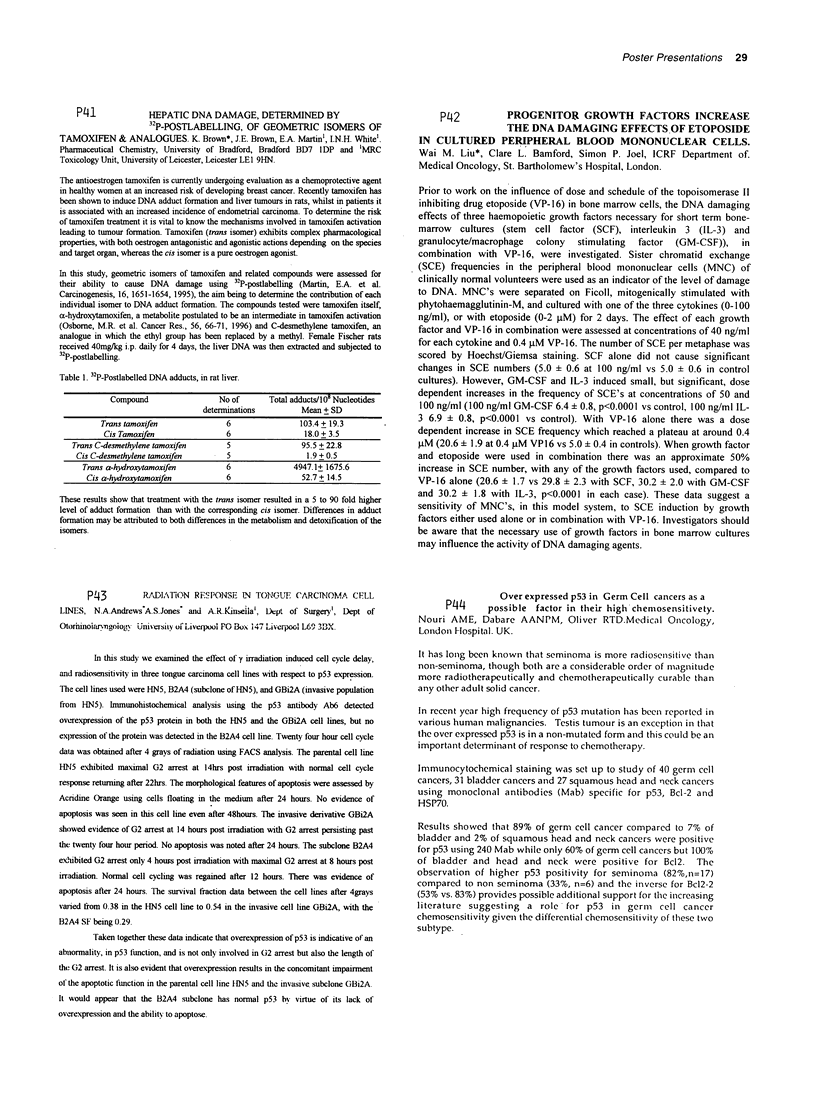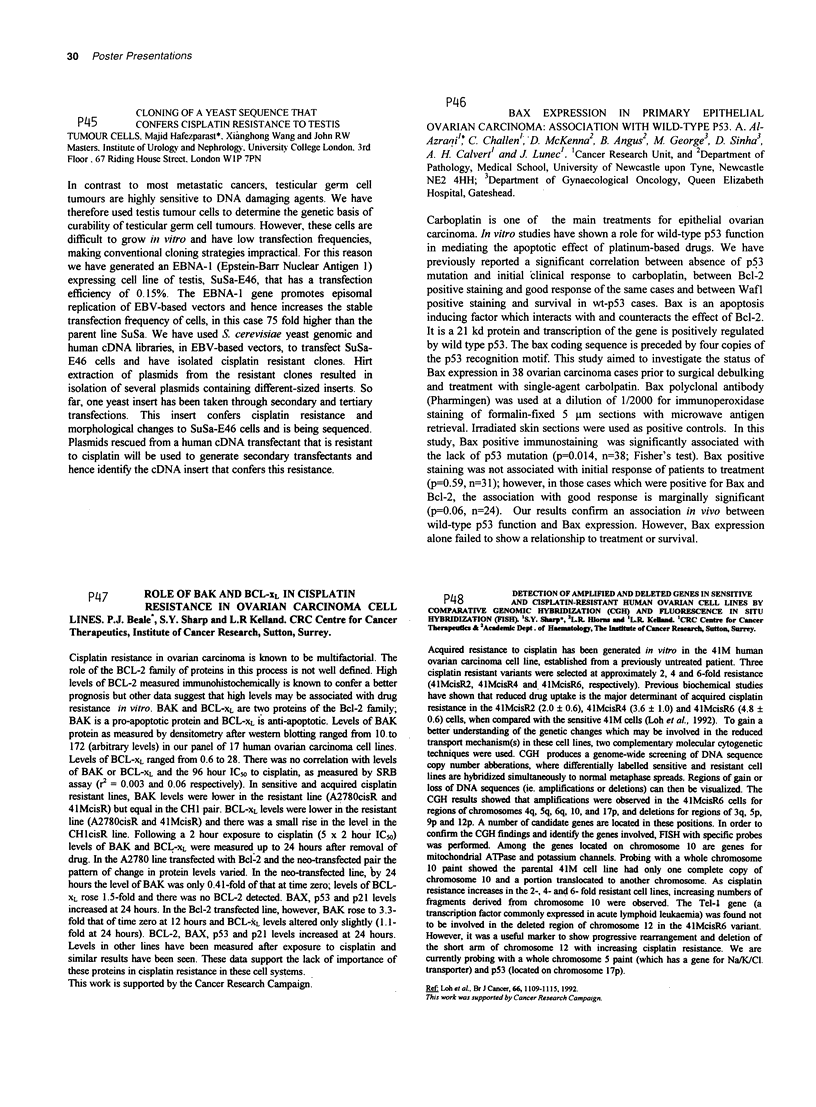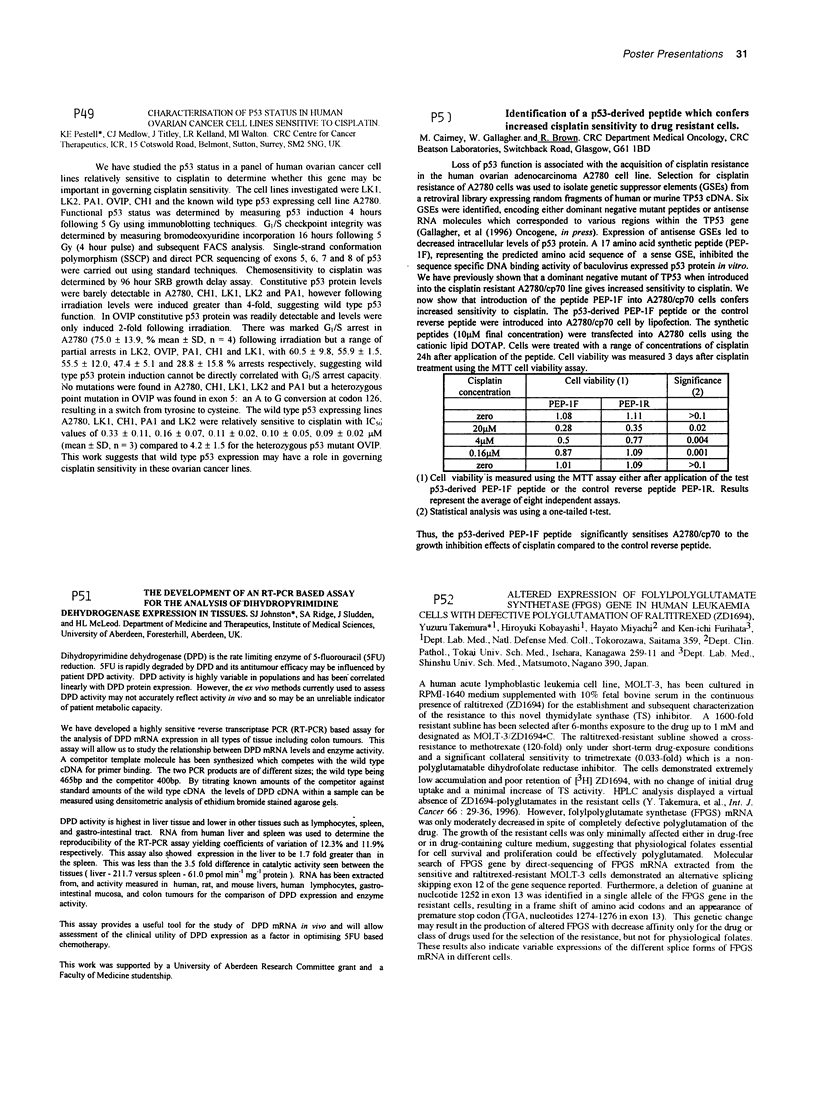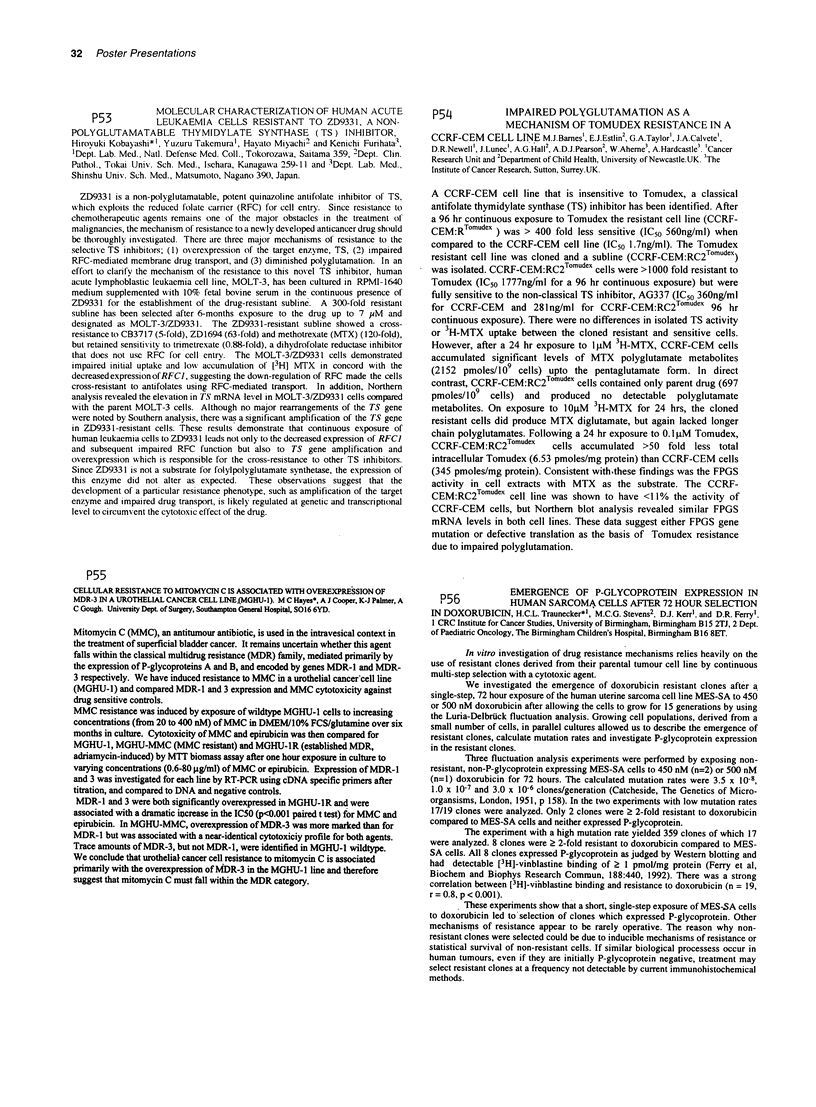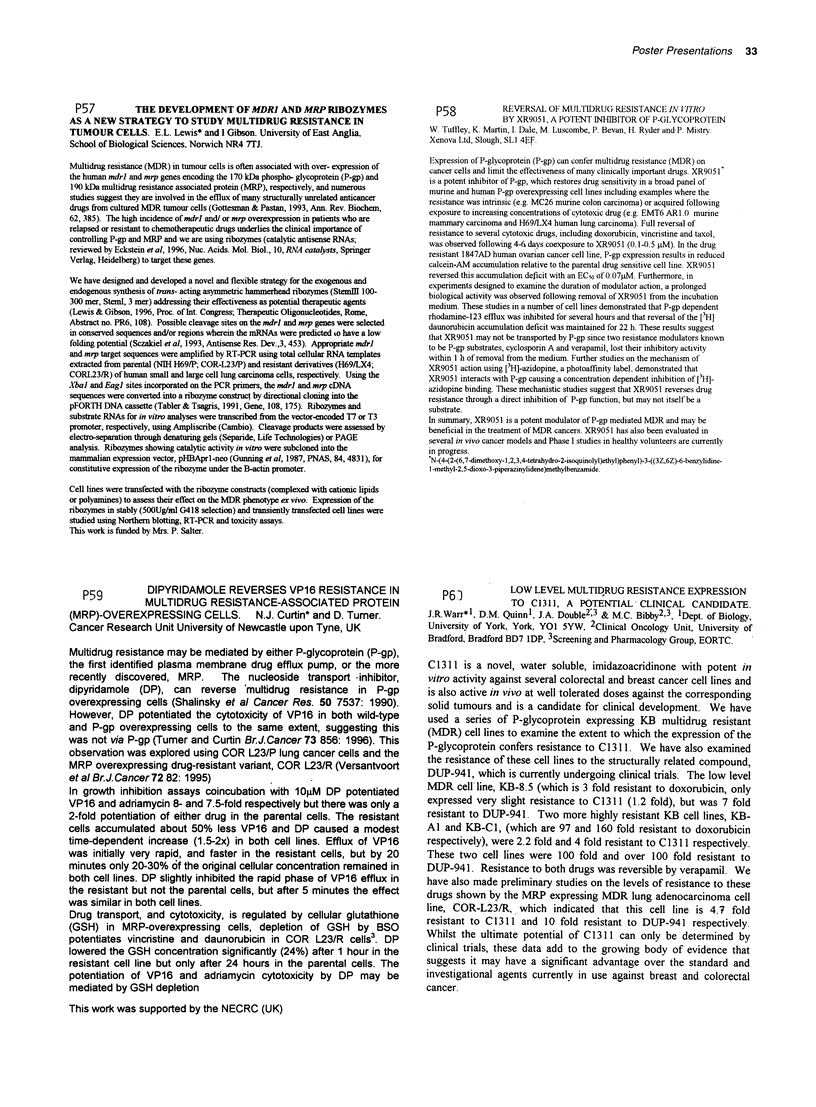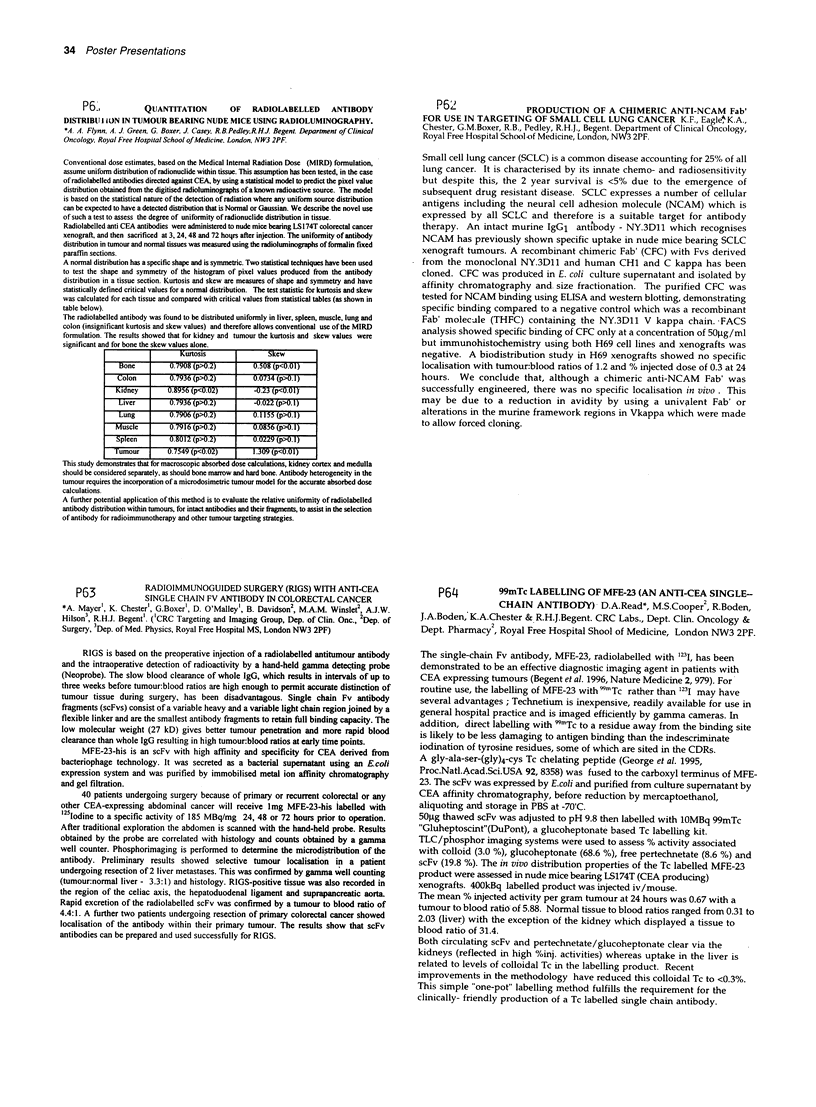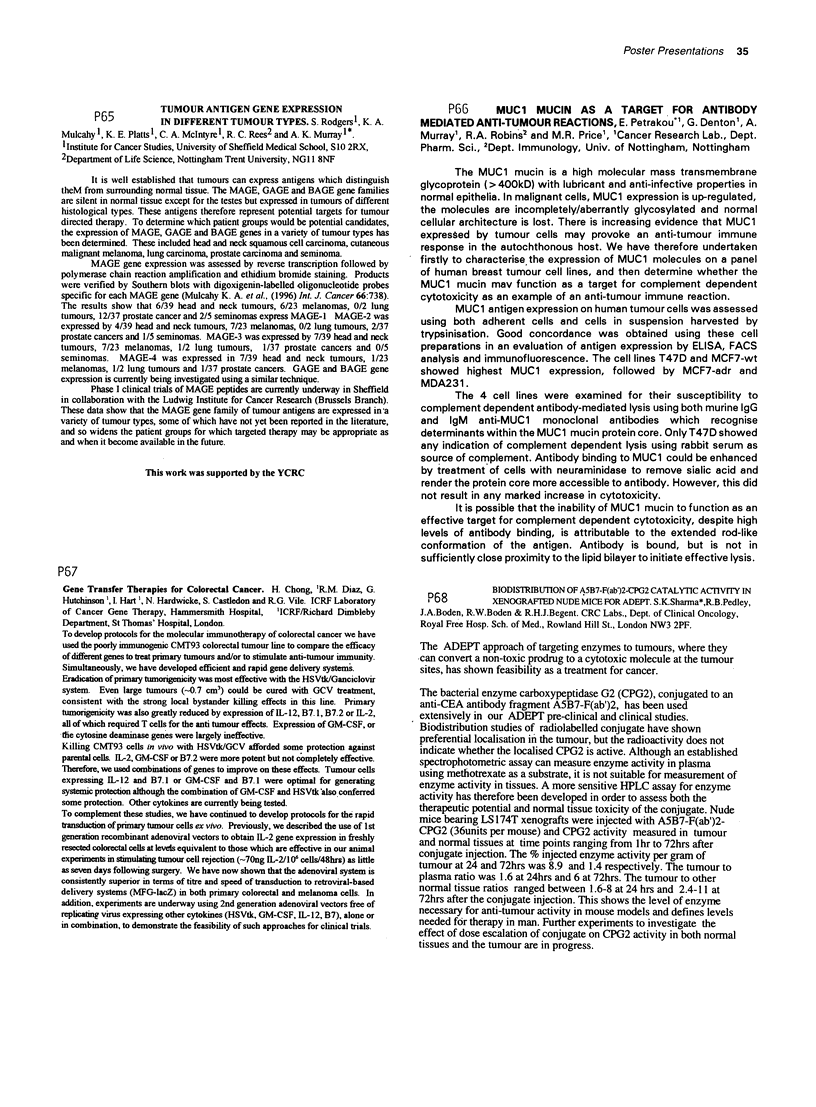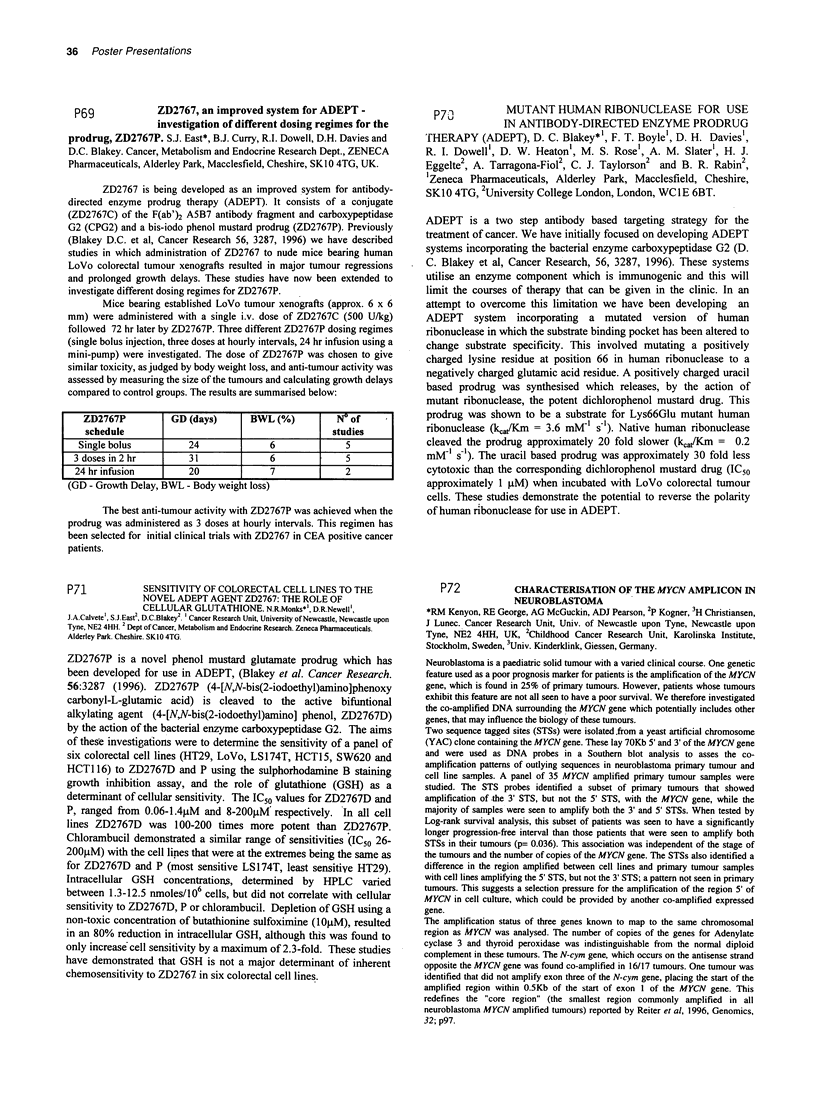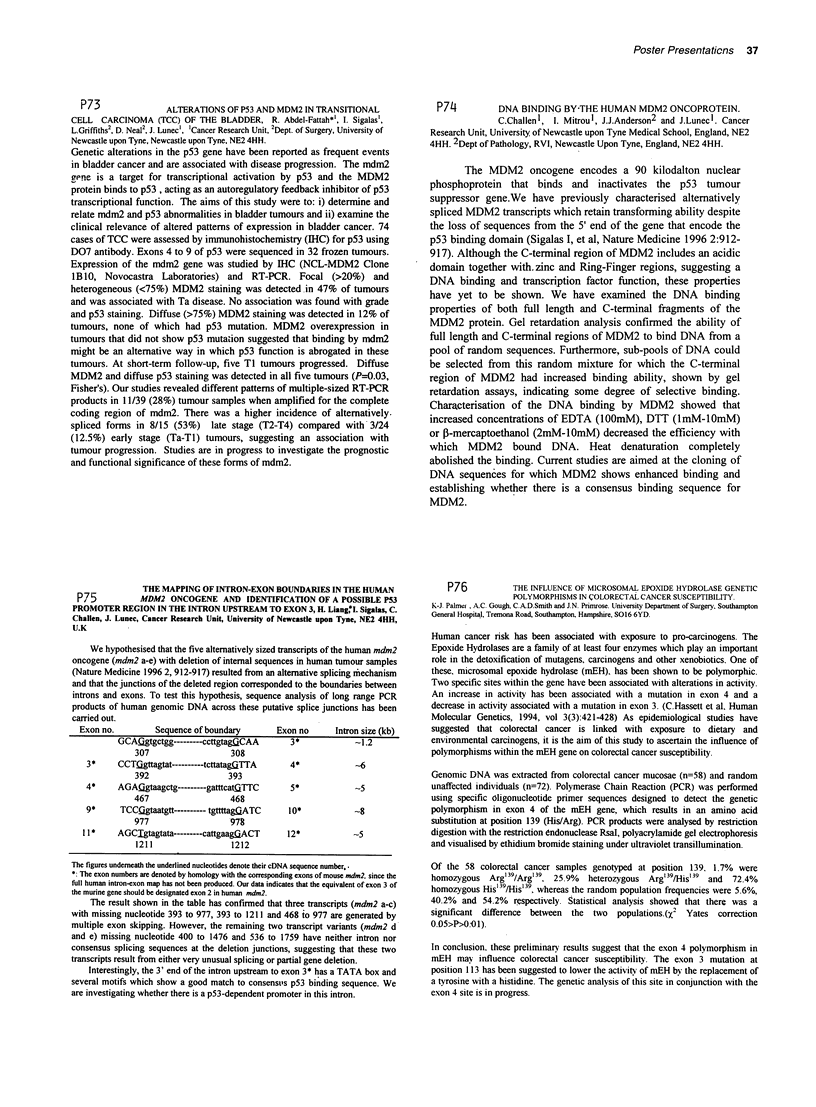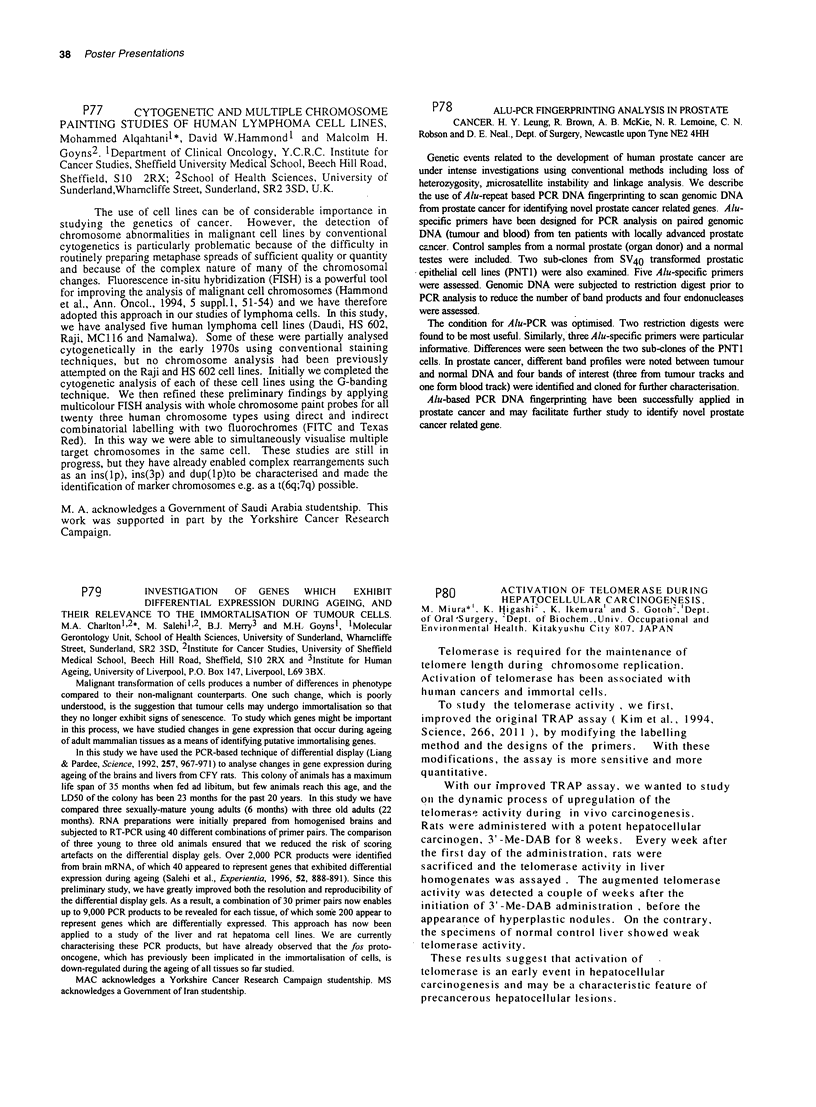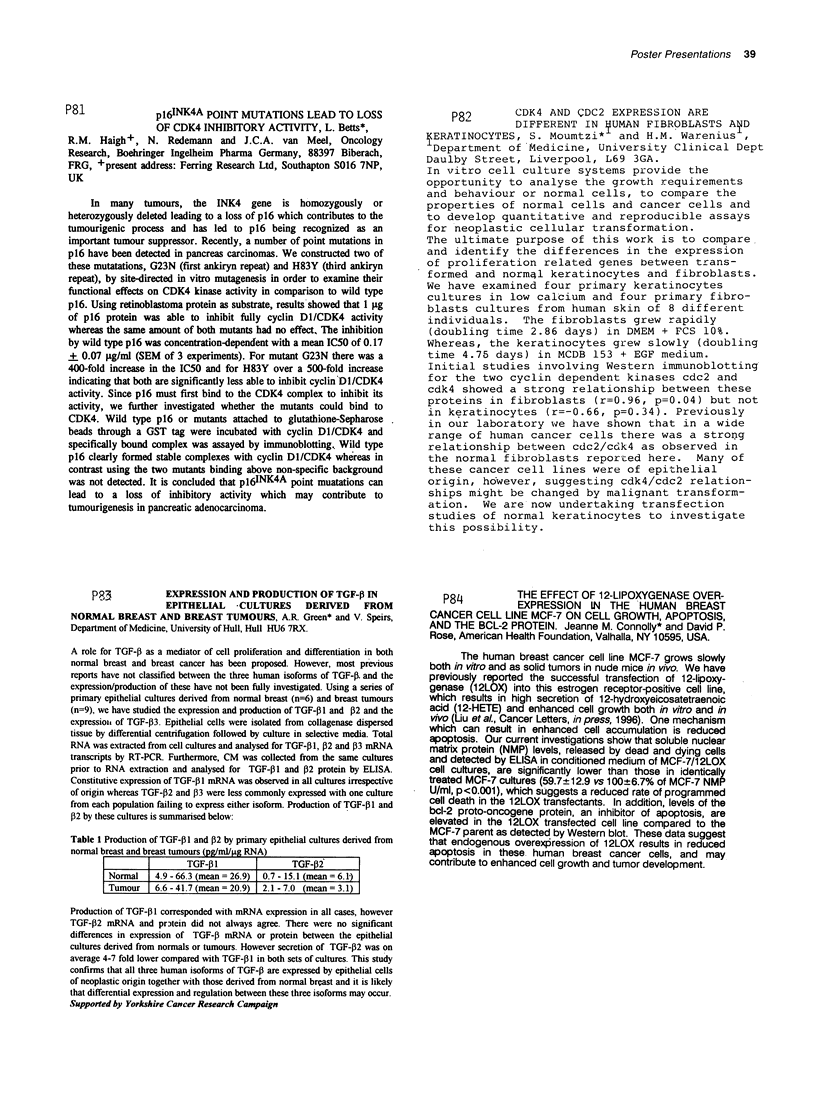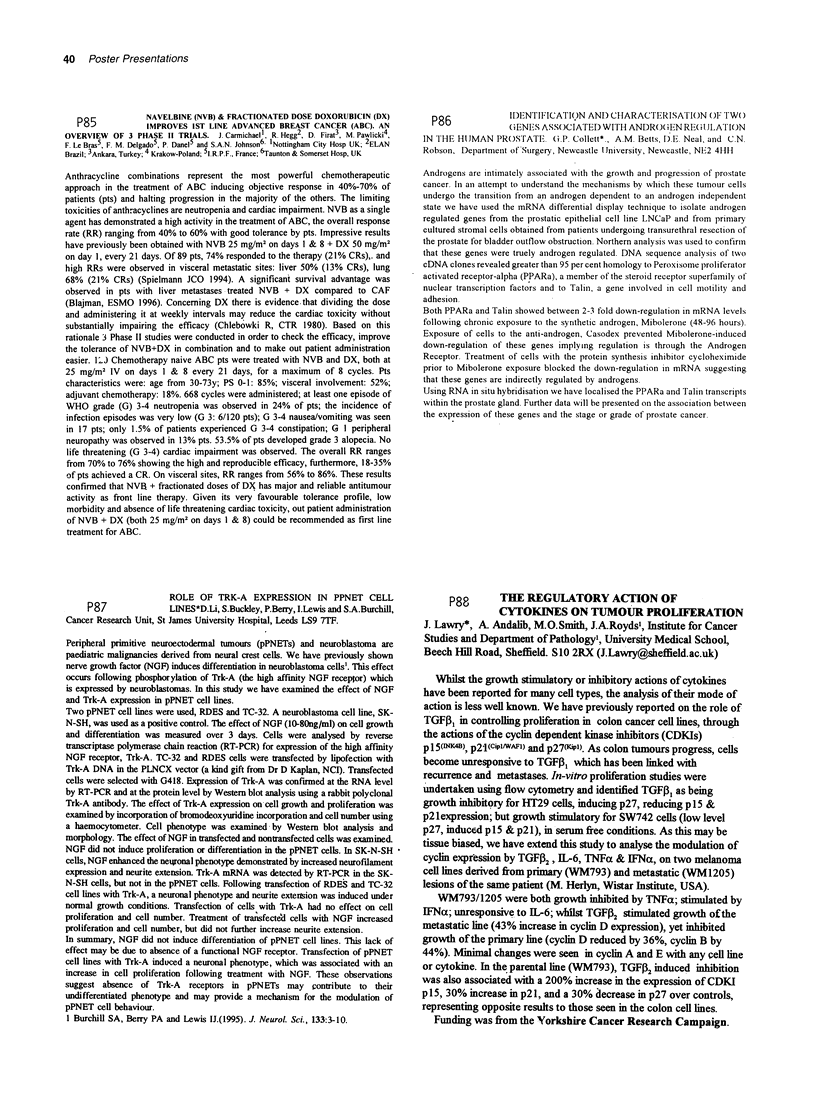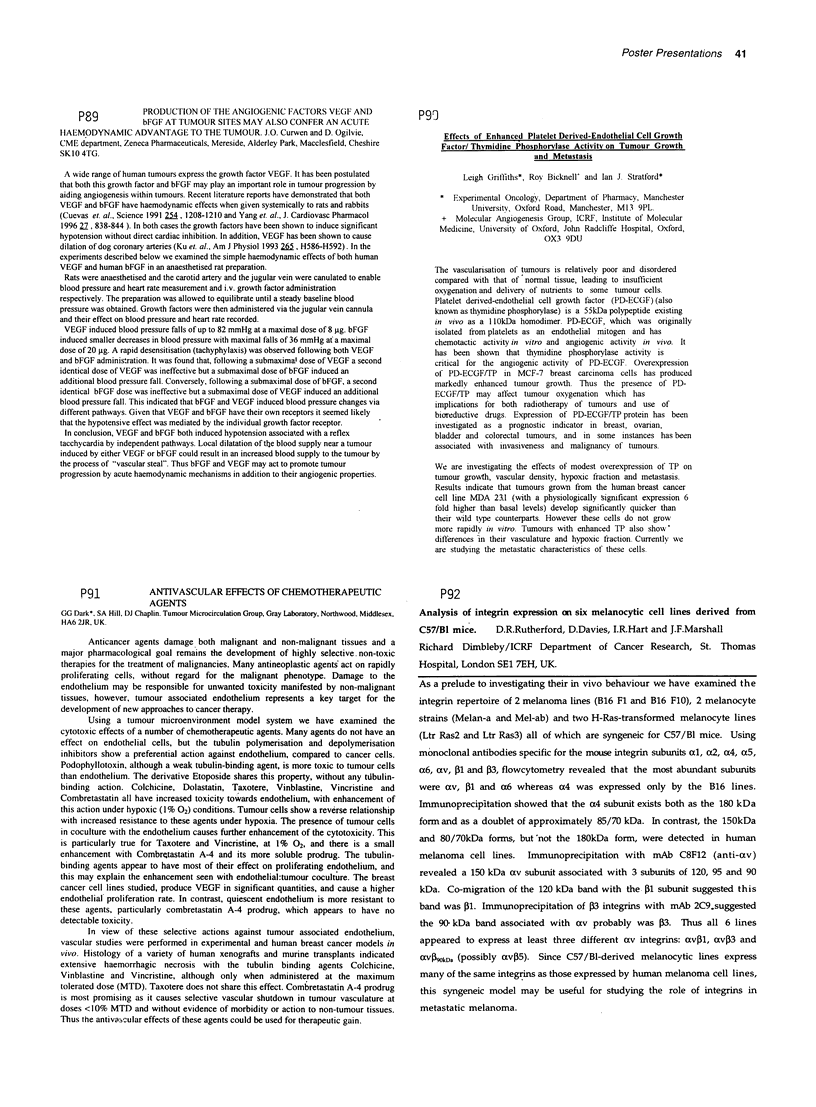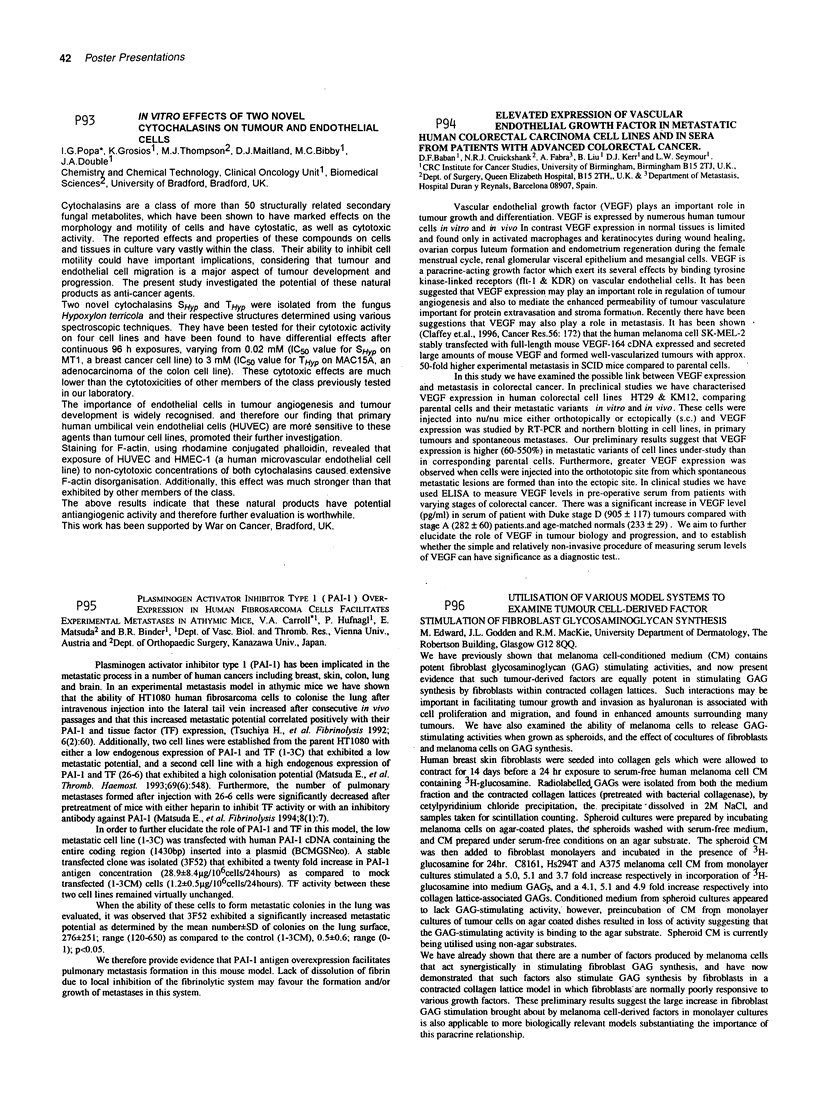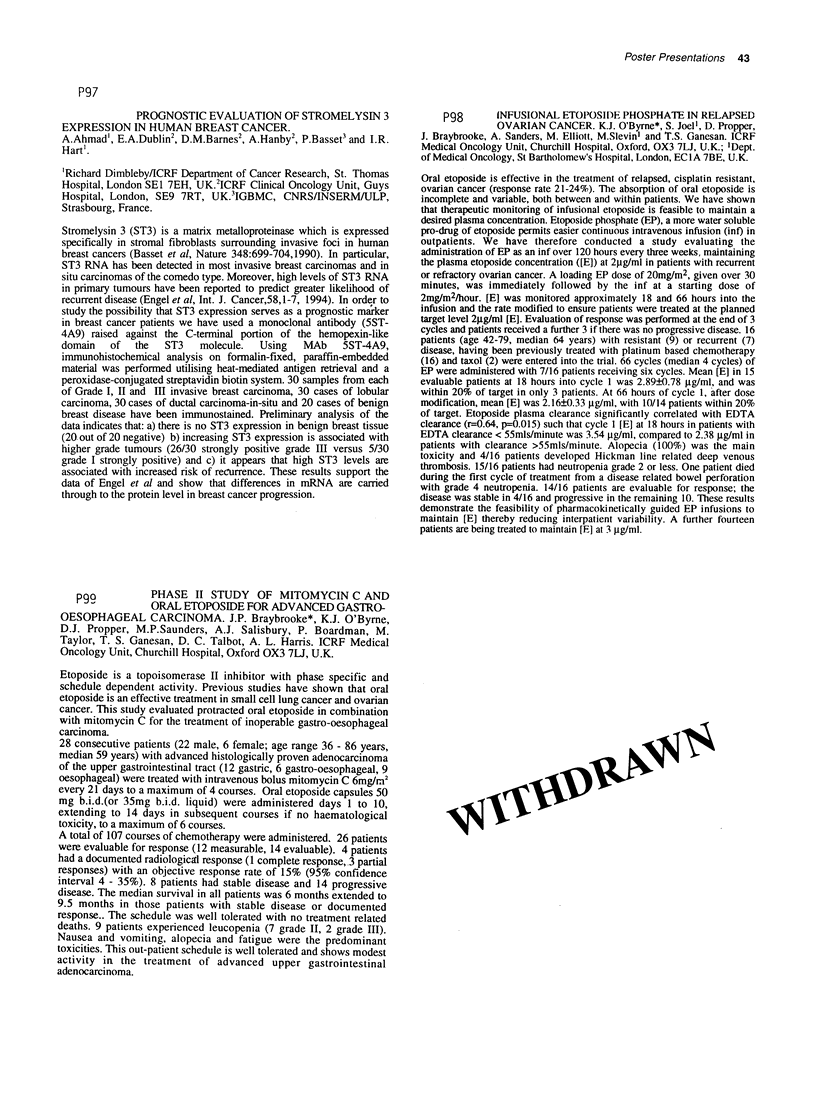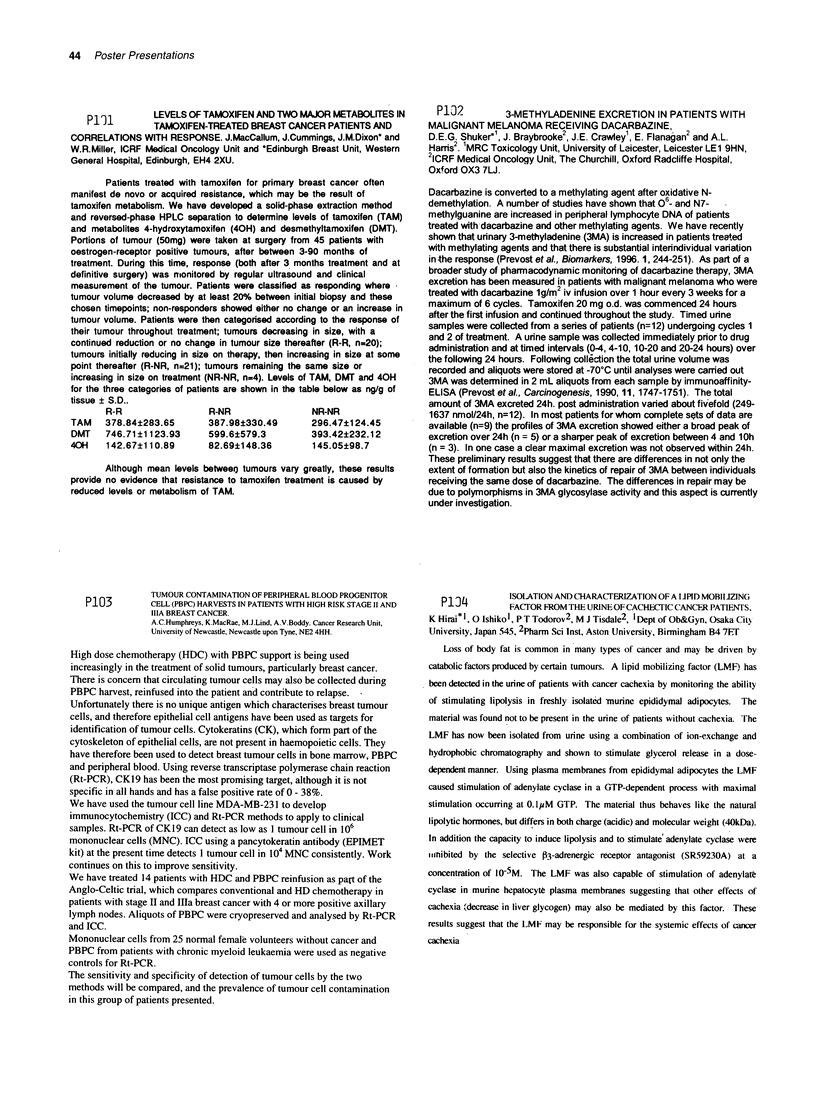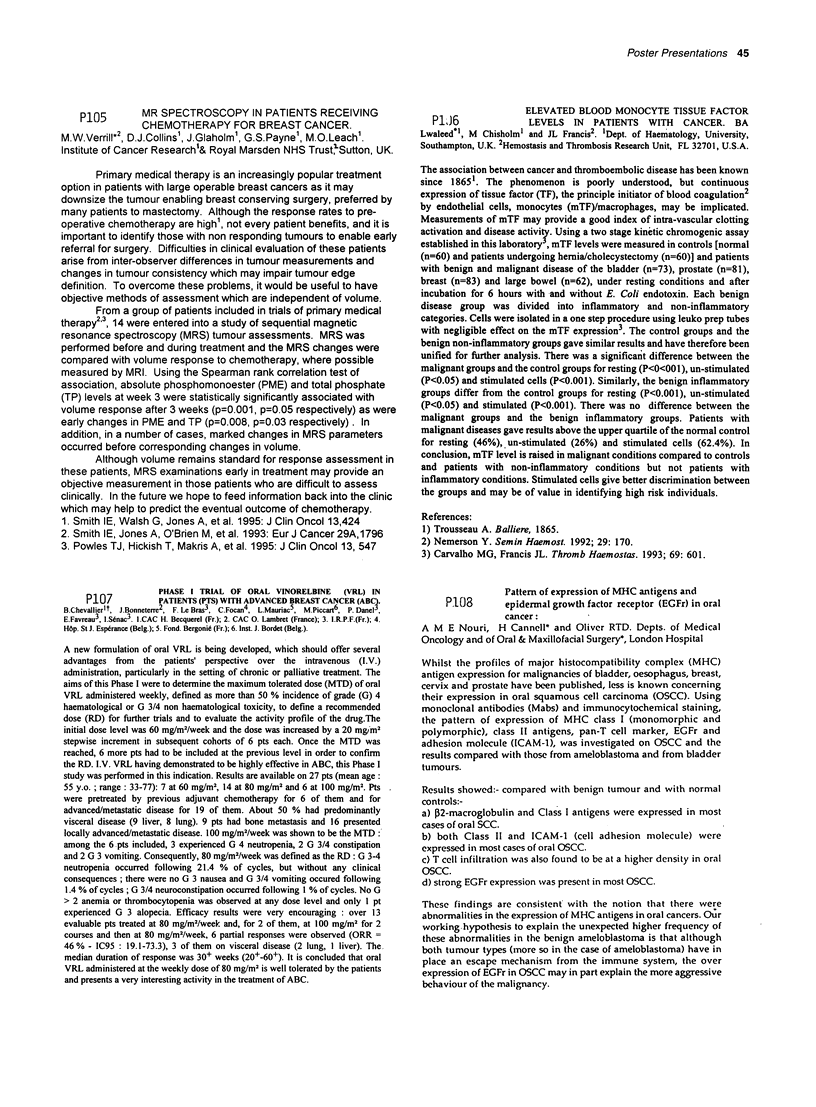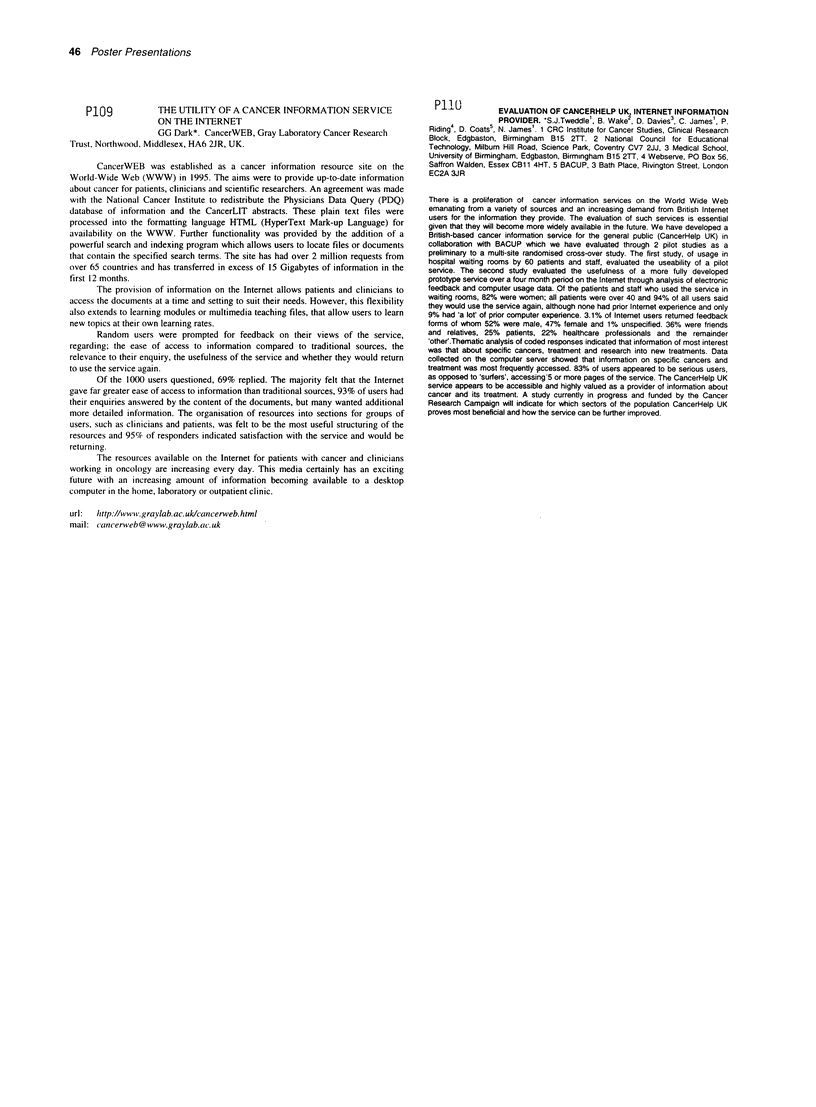# Poster presentations

**Published:** 1997

**Authors:** 


### P2 EFFECTS OF THE PHOSPHATIDYLINOSITOL 3-KINASE FAMILY INHIBITOR WORTMANNIN ON THE CYTOTOXIC MECHANISMS OF ETOPOSIDE IN A549 NSCLC CELLS

S. Boulton, S. Kyle and B.W. Durkacz*. Cancer Research Unit, University of Newcastle-Upon-Tyne, NE2 4HH.

DNA dependent protein kinase (DNA-PK) shares homology with the phosphatidyl 3-kinase (PI 3-K) gene family, which also includes ataxia telangiectasia (AT) gene product. DNA-PK is required for DNA double strand break (DSB) repair. DSBs contribute to the cytotoxic effects of ionising irradiation (IR). Wortmannin (WM) is an inhibitor of DNA-PK and other enzymes of the PI 3-K family. We have demonstrated that in CHO-K1 cells, IR-induced cytotoxicity was potentiated by non-toxic concentrations of WM ($DEF_{10}$* values of 2.7 ± 0.3μM & 5.3 ± 0.9μM for 5μM & 20μM WM, respectively) and the repair of DSB was completely inhibited (Boulton *et al* (1996), *Carcinogenesis* **17**: 2285). These data implicate DNA-PK inhibition and the consequent inhibition of DSB repair as the reason WM potentiates the cytotoxicity of IR. WM also inhibits the p110 PI 3-K, an enzyme which transduces growth factor responses mediated by tyrosine kinases. Flow cytometry analysis demonstrated that WM (50μM) caused an accumulation of cells in G1, but this was a transient effect, presumably reflecting the instability of WM in culture medium, with resumption of the cell cycle observed after 16 hr.

A mutant cell line, *xrs-6*, which lacks DNA-PK activity due to the absence of Ku 86 subunit, exhibits hypersensitivity to etoposide, a topoisomerase II inhibitor (Jeggo *et al* (1989), *Cancer Res.* **49**: 7057). We investigated the effect of WM on etoposide, in A549 NSCLC cells. WM (20μM) potentiated etoposide cytotoxicity with a $DEF_{10}$ of 1.48 ± 0.12μM. A 1 hr exposure to etoposide resulted in a concentration dependent increase in DSB levels. WM had no effect on the DSB levels at this time. However, following a 1 hr incubation with 20μM etoposide, DSB repair over a 3 hr period was retarded by coincubation with WM. This delay was more pronounced at the later repair times, e.g. 61% compared to 69% DSB remaining at 30 min, and 25% compared to 43% at 2 hr, for etoposide and etoposide + WM, respectively.

Topoisomerase II inhibitors have been shown to cause an early inhibition of DNA synthesis, and this effect is abolished in AT cells (Kaufmann *et al* (1991) *Mol. Cell Biol.* **11**: 3711). Preliminary data indicates that WM (50μM) prevents this inhibitory effect. These data suggest that WM may also be acting to inhibit the AT PI 3-kinase as part of the cytotoxic mechanism.

* $DEF_{10}$: dose enhancement factor at 10% survival.

### P3 ELUCIDATION OF THE SIGNAL TRANSDUCTION PATHWAY OF A TUMOUR-DERIVED LIPID-MOBILISING FACTOR

S Khan, SA Price*, MJ Tisdale, Pharmaceutical Sciences Institute, Aston University, B'ham B4 7ET.

A cytosolic extract of a cachexia-inducing murine adenocarcinoma (MAC16) was found to stimulate lipolysis in isolated murine white adipocytes, and adenylate cyclase (AC) in adipocyte plasma membrane preparations, in a dose dependent fashion. The effect upon AC activity exhibited responsiveness to GTP, with low concentrations (0.1μM) causing stimulation whilst high concentrations (10μM) proved inhibitory, suggesting the influences of both stimulatory (Gs) and inhibitory (Gi) guanine nucleotide-binding proteins. Stimulation of lipolysis or AC activity by both isoprenaline and the tumour-derived lipid-mobilising factor (LMF) was significantly enhanced in adipocytes isolated from cachectic MAC16-bearing mice. However, there was no measurable increase in the stimulation of AC activity by forskolin, indicating that the up-regulated response observed in the cachectic state resulted from an alteration in either the number or activity of receptors or G-proteins. The induction of lipolysis by LMF was effectively inhibited by pre-treating adipocytes with the non-selective ß-receptor antagonist propranolol (10μM). Further studies employing the selective ß3-receptor antagonist SR59230A, revealed that pre-treatment of either isolated adipocytes, or plasma membrane fractions with this drug (10μM) significantly attenuated the effects of both isoprenaline and LMF. Given the up-regulated response observed in the cachectic state, the biphasic role of GTP, and the inhibition produced by both propranolol and SR59230A, it would seem probable that the effects of LMF are mediated via a ß3-adrenergic receptor. Therefore, the lipolysis observed in white adipose tissue during tumour-induced cachexia appears to be the result of a tumour-derived lipid-mobilising factor which acts to stimulate cyclic AMP formation in a similar manner to natural lipolytic hormones.

### P4 LACK OF PENETRATION THROUGH MULTICELL LAYERS IS A CONTRIBUTING FACTOR TO THE FAILURE OF EO9 IN THE CLINIC.

R.M.Phillips*, P.M.Loadman and B.P.Cronin. Clinical Oncology Unit, University of Bradford, Bradford BD7 1DP.

The indoloquinone compound EO9 (3-hydroxy-5-aziridinyl-1-methyl-2-[1H indole-4,7-dione]prop-β-en-α-ol) is a bioreductive drug which is activated by the enzyme DT-diaphorase (EC 1.6.99.2) to a DNA damaging species. EO9 was selected for clinical trial based upon its mechanism of action together with evidence of activity against solid tumours *in vitro* and *in vivo* and its lack of myelosuppresion in animal studies (Hendriks *et al*, Eur J Cancer, 29A: 897-906, 1993). Despite reports of 3 partial remissions in phase I trials, recent phase II trials have demonstrated that EO9 is inactive against a variety of human cancers (Dirix *et al*, Eur J Cancer, 32A, 2019-2022, 1996). A key parameter for bioreductive drugs is that they must be able to penetrate through several layers of cells in order to reach their target. The aim of this study was to determine whether or not drug penetration barriers could explain the lack of activity of EO9 in the clinic. We have developed an *in vitro* model to study drug penetration based upon the assay described by Cowan *et al* (Br.J.Cancer, 74 (suppl XXVII), 28-32, 1996) where cells are grown in transwell culture inserts (Costar) forming an upper and lower chamber separated by a microporous membrane. Drugs were added to the top chamber and the concentration of drug appearing in the lower chamber was determined by HPLC as a function of time. DLD-1 human colon carcinoma cells (2.5 x $10^5$ cells) were initially seeded into the top chamber and grown for several days with daily medium changes. The thickness of the cell layer was determined by routine histological procedures and cell layers of 15.0 ± 6.2 μm to 78.3 ± 10.1 μm thickness were obtained on days 1 and 8 of the growth curve respectively. EO9 (10 μM) was added to the top chamber and the rate at which EO9 crossed the cell layer was inversely proportional to the thickness of the cell layer. Cell layers of 78.3 ± 10.1 μm completely blocked the passage of EO9 across the membrane with no EO9 detectable in the lower chamber following a 3 hour incubation at 37°C. At 50.3 ± 10.1 μm, EO9 was only detectable (< 0.5 μM) in the lower chamber 30 min after drug administration. EO9 has an extremely short half life *in vivo* (3min in mice and 10 min in humans) and the results of this study suggest that EO9 is unlikely to reach the hypoxic fraction of tumours within the pharmacokinetic lifespan of the drug. Poor penetration into tumours in conjunction with rapid clearance may therefore play a major role in EO9's disappointing clinical activity. Improving drug delivery or developing analogues which have the same desirable properties as EO9 (ie bioactivation by DT-diaphorase) but have better pharmacological properties may result in drugs which are capable of exploiting elevated DT-diaphorase levels which exist in certain tumours.

### P5 SPECIES DEPENDENT DIFFERENCES IN THE METABOLISM OF INDOLOQUINONES BY WHOLE BLOOD.

R.M.Phillips*, P.M.Loadman and C.M.Jarrett. Clinical Oncology Unit, University of Bradford, Bradford BD7 1DP.

The indoloquinone compound EO9 (3-hydroxy-5-aziridinyl-1-methyl-2-[1H indole-4,7-dione]prop-β-en-α-ol) has an extremely short plasma half life in both rodents (T½ = 3 mins) and humans (T½ = 10 mins). The rapid clearance in conjunction with evidence demonstrating that drug penetration barriers exist suggest that the lack of anti-tumour activity in the clinic may be caused by pharmacological as opposed to pharmacodynamic problems. Blood cells are known to influence the pharmacology of drugs and the aim of this study was to determine whether or not the rapid clearance of EO9 *in vivo* can be attributed to metabolism by blood cells. The stability of EO9 in human and murine (NMRI mice) blood was determined by HPLC at 37$^{o}$C. In phosphate buffered saline (pH 7.4), EO9 has a half life of 8.5 h with EO5A being the primary breakdown product. In both human and murine plasma EO9 was stable over the duration of the experiment (T½ > 3 h). In murine whole blood however, EO9 was rapidly removed (T½ = 28 mins) whereas in human whole blood, EO9 was relatively stable (T½ = 2 h). No EO5A could be detected in both human and murine blood samples. In heat treated murine and human blood (56$^{o}$C for 20 min), EO9 was stable (T½ > 3 h) suggesting that EO9 is metabolised by whole blood. DT-diaphorase activity was detected in murine blood (16.7 ± 1.9 nmol/min/mg) but no activity (< 0.1 nmol/min/mg) was detected in human blood. Cytochrome $b_5$ reductase activity was also elevated in murine (3.05 ± 0.5 nmol/min/mg) compared with human blood (1.16 ± 0.2 nmol/min/mg). Levels of cytochrome P450 reductase were comparable in both murine and human blood (1.1 and 0.7 nmol/min/mg respectively). Species dependent differences in enzymology may therefore contribute to different half lives of EO9 in human and murine blood. Several analogues of EO9 were evaluated in this study and all compounds tested were unstable in murine blood compared with human blood. EO68 in particular had an extremely short half life in mouse blood (T½ < 2 mins) but was relatively stable in human blood (T½ > 2 h). As EO68 is a good substrate for DT-diaphorase and is selectively toxic towards DT-diaphorase rich cells (Phillips, Biochem. Pharmacol, 52, 1711-1718, 1996), these results have significant implications with regards to the use of murine tumour models to evaluate potential novel analogues of EO9. In conclusion, the results of this study suggest that metabolism of EO9 by whole blood may contribute to the rapid clearance of EO9 in rodents but not humans. Species dependent differences in the rates of metabolism of EO9 and its analogues suggest that the use of murine tumour models may give rise to false negative results and care needs to be taken in selecting experimental models for evaluating novel indoloquinones.

### P6 *IN VITRO* EVALUATION OF COMBRETASTATIN A4 AND ITS ANALOGUE.

K. Grosios*, A.T. McGown[1], G.R. Pettit[2], M.C. Bibby

Clinical Oncology Unit, University of Bradford, Bradford BD7 1DP, UK

[1]Paterson Institute for Cancer Research, Manchester M20 9BA, UK

[2]Arizona State University, Tempe, Arizona 85287-1604, USA

Tumour angiogenesis is an essential process in tumour development and progression. Development of antiangiogenic or antivascular agents could therefore have a major impact in cancer therapy. Currently, *in vitro* screening for antiangiogenic agents is very limited although endothelial cell culture systems provide useful models for the investigation of such compounds. *In vivo* work with combretastatin A4 and its phosphate analogue demonstrated that both drugs cause extensive haemorrhagic necrosis in experimental tumours. In this study, *in vitro* evaluation of these two agents was carried out using human umbilical vein endothelial cell (HUVEC) cultures. Initial experiments using the Neutral Red assay, showed that 0.31µM and lower concentrations of combretastatin A4 and its analogue were not cytotoxic to HUVEC (77.79% and 96.26% cell survival was obtained respectively at 0.31µM concentration). Both compounds caused inhibition of HUVEC migration through collagen, in transwell chambers as compared to control, untreated cells. Endothelial cells grown in between type I calf-skin collagen, form cell networks. Such networks were significantly disturbed after 4h exposure to 0.31µM of both agents. Disruption was observed with even lower concentrations and was complete after 24h. Investigation of the cellular mechanism of action of these compounds using staining of F-actin (using rhodamine conjugated phalloidin), revealed that at the same, non-cytotoxic concentration, 4h treatment with both compounds resulted in high levels of F-actin disorganisation and rounding up of the endothelial cells. These *in vitro* results, strongly indicate the antiangiogenic potential of combretastatin A4 and its phosphate analogue and justify further evaluation.

This work has been supported by War on Cancer, Bradford, UK.

### P7 STUDIES ON THE INTERACTIONS BETWEEN DNA AND NOVEL POLYCYCLIC ACRIDINE DERIVATIVES.

E. Giménez-Arnau*, S. Missailidis, D. J. Hagan, J. Stanslas and M. F. G. Stevens, Cancer Research Laboratories, Department of Pharmaceutical Sciences, University of Nottingham, Nottingham NG7 2RD.

In the past few years, acridine-based derivatives have formed the basis for the development of agents which target DNA topoisomerases, enzymes which resolve topological constraints in DNA and, consequently, considered valid targets in drug design. [1,2]

We have developed a novel synthetic route to the polycyclic frameworks of tetracyclic (**1**), pentacyclic (**2**) and hexacyclic acridines (**3**) with the intention of devising molecules with appropiate substituents (R) which can discriminate between topoisomerase II α and β isoforms.

Structures of polycyclic acridines

(1) (2) (3)

The interaction between DNA and compounds (**1**)-(**3**) has been studied using a range of physicochemical techniques such as spectrophotometric analysis, fluorescence quenching, thermal denaturation, circular and linear dichroism and X-ray crystallography. Results with compounds (**2**) (R=H, Cl, $CH_3$, $NH_2$) confirm: (i) strong DNA-binding affinity, 3-100 fold greater than that of *m*-AMSA; (ii) binding in an intercalative mode at high DNA to drug ratios; (iii) A-T base-pair binding preference; (iv) disordered binding at low DNA to drug ratios. Even without appropiate structural embellishments (R), the compounds show sub-micromolar $IC_{50}$ values against an extensive panel of cell lines *in vitro* and cytotoxicity correlates with DNA-binding affinity and topoisomerase II inhibition.

1. Denny, W. A.; Baguley, B. C.; Cain, B. F.; Waring, M. J. Antitumor Acridines. In *Molecular Aspects of Anticancer Drug Action*, 1st ed.; Neidle, S., Waring, M. J., Eds.; Macmillan Publishing Co.; London, 1983, pp 1-34.

2. D'Arpa, P.; Liu, L. F. Topoisomerase-targeting Antitumor Drugs. *Biochim. Biophys. Acta* 1989, *989*, 163-177.

### P8 IFOSFAMIDE METABOLISM AND ITS EFFECT ON DNA IN TUMOUR CELLS *IN VITRO* AND *IN VIVO*.

I.A. Willits[1], S.M. Yule[2], A.D.J. Pearson[2], L. Price[2], C. Brock[2], R. Wyllie[2], S. Cholerton[3] and A. V. Boddy[1], [1]Cancer Research Unit, [2]Dept Child Health, [3]Dept Pharmacological Sci, University of Newcastle Upon Tyne NE2 4HH.

The anticancer drug ifosfamide (IF) is widely used in the treatment of paediatric malignancy. It is a prodrug requiring metabolic activation to produce 4-hydroxyifosfamide (4-OHIF), which then spontaneously breaks down to form the ultimate alkylating species, isophosphoramide mustard (IPM). It is believed that the cytotoxic action of IPM is due to its binding to DNA, forming a range of lesions which inhibit cell replication. The effect of these metabolites on DNA damage was investigated *in vitro* in the lymphoid leukaemia cell line CCRF-CEM using the single cell gel electrophoresis (SCGE) assay. The extent of DNA damage was measured as the tail moment (TM). Cells exposed to gamma irradiation showed a linear increase in TM up to 10 Gy. Cell inhibition studies showed that the $IC_{50}$ of CCRF-CEM cells exposed to 4-OHIF and IPM was 20 and 250 µM respectively, whilst IF exhibited no effect at millimolar concentrations. Cells were exposed to IPM or 4-OHIF at $IC_{50}$ and 4x$IC_{50}$ concentrations for 1h and samples were taken at 1h, 24h, 48h and 72h for SCGE analysis. No increase in TM was detected after 1h, but all samples showed significant increases after 1 day compared to controls. Cells incubated with the $IC_{50}$ concentration of drugs showed resolution of TM after 72h, whilst those incubated with 4x$IC_{50}$ showed continued damage. Studies on the nuclear morphology of cells using Hoescht dye fluorescence revealed both metabolites caused extensive apoptosis which was not directly related to TM. Ifosfamide metabolism was investigated in 11 children receiving chemotherapy using thin-layer chromatography. Subjects received IF at 6 or 9g/m$^2$ administered over 2 or 3 days either as a continuous infusion or as 3 hour daily infusions. Peripheral blood lymphocytes (PBLs) were collected at regular intervals throughout the course and after 3 weeks for analysis with SCGE. A high degree of inter-patient variation in terms of both IF metabolism and DNA damage was seen. In all cases DNA damage accumulated throughout the course and resolved after 3 weeks. DNA damage peaked at 48h in 7 patients. There was no difference in mean TM values between the different administration schedules, nor was any relationship between PBL TM and metabolite production observed. These results verify that IF is not cytotoxic *per se* but its activity is due to its metabolites. These metabolites cause DNA lesions and trigger apoptosis in CCRF-CEM cells. When IF is introduced systemically in patients DNA damage is measurable in PBLs, but this is not directly related to measurable metabolites.

P9 ASSESSMENT OF DNA DAMAGE CAUSED BY IFOSFAMIDE AND METABOLITES IN PATIENTS AND IN MCF-7 CELLS

E.C. Johnstone*, M.J. Lind, N. Siddiqui, F. Chapman, A.V. Boddy*. Cancer Research Unit (*) and Northern Centre for Cancer Treatment, Newcastle upon Tyne NE2 4HH.

Ifosfamide (IF) is a widely-used oxazophosphorine which has demonstrated activity against advanced breast cancer. It is metabolically activated to 4-hydroxyifosfamide (4OH ifo), which is unstable and subsequently breaks down to isophosphoramide mustard (IPM), an alkylating species which causes DNA damage via crosslinks and monofunctional adducts. The production of IPM is thought to occur intracellularly as IPM is ionised at physiological pH and thus its cellular uptake may be hindered. In a clinical study to determine the DNA damaging properties of ifosfamide, patients with advanced or metastatic breast cancer were treated with IF ($5g/m^2$ as a 24 hour infusion) in combination with doxorubicin (DX) ($40mg/m^2$ bolus). DNA damage was detected in the peripheral blood lymphocytes (PBL) and also in the tumour cells of patients using single cell gel electrophoresis (the comet assay). DNA damage in PBL's showed an increase 24h into treatment compared to basal damage. A subsequent increase to maximum damage at 48h was seen in all cases. Complete resolution of damage 3 wk after treatment was observed in 50% of patients. In tumour cells, DNA damage at pre treatment and 24h after the start of infusion showed a similar distribution. However a definite shift towards increased damage at 3wk post treatment was observed. In growth inhibition studies *in vitro*, the breast cancer MCF-7 cell line was approximately 4 fold more sensitive to 4OH ifo than to IPM ($IC_{50}$ 1.5μM vs 6.2μM). The time course of DNA damage was investigated in MCF-7 cells; on exposure to 6μM 4-hydroperoxyifosfamide (4 x $IC_{50}$), maximum damage occurred at 48h, similar to the *in vivo* situation with PBL. The mean of the maximum 4-hydroxyifosfamide concentrations *in vivo* was determined to be 5.4μM ± 2.0μM using a modification of the acrolein fluorescnce assay, and this reflects the concentrations used *in vitro*. Investigations of the pharmacokinetics of IF and DX have also been performed on these patients for comparison with DNA damage in PBL and tumour.

P10 CHARACTERISATION OF A POLYCLONAL ANTIBODY RAISED TO DNA ADDUCTS OF AMD473.

C.B. Miner*, F.I. Raynaud, J. Holford, L.R. Kelland, A. Hardcastle, G.W. Aherne. CRC Centre for Cancer Therapeutics at the Institute of Cancer Research, Sutton, Surrey, UK.

AMD473 [amminedichloro(2-methylpyridine) platinum (II)] will begin Phase I clinical trial at the Royal Marsden NHS Trust in 1997. The compound has been found to be active in cisplatin resistant models. Its development, as a novel sterically hindered platinum (II) complex, was undertaken to reduce intracellular deactivation by thiols, a frequent mechanism of drug resistance in tumours. It is known to possess some unique DNA binding properties in comparison to cisplatin. Less overall DNA binding (e.g. in the CH1 human ovarian carcinoma cell line there was 2.8 fold higher platination with cisplatin than with AMD473 following 2h exposure to 50, 100 and 200 μM) of AMD473, in comparison to cisplatin (CDDP), indicates that a more sensitive detection method than classical flameless atomic absorption spectroscopy is required. The lower levels of DNA binding in conjunction with different adducts formed has prompted the raising of a polyclonal rabbit antibody to AMD473 adducts of DNA to develop an immunoassay with improved detection for clinical samples. Animals were initially immunised with AMD473 platinated DNA complexed with methylated BSA and then emulsified with Non-ulcerative Freund's adjuvant. Sensitivity in the femtomolar AMD473-DNA adduct range has been reproducibly achieved using competitive colourimetric enzyme-linked immunosorbent assay (ELISA), using serum obtained after a single booster immunisation. A standard curve was produced in the competitive platinum concentration range of 12.5-125000 fmole/assay well and antiserum dilution of 1/2000. A 50% inhibition at approximately 1.9 pmole AMD473-DNA adduct/assay well was obtained. No cross reactivity was observed with parent compound, DNA, CDDP or conjugated DNA-CDDP, without purification of the antiserum.
Characterisation of the types of DNA adducts formed/detected has begun, using selected platinated polynucleotides as cross reactants in immuno-inhibition studies. Initial results of these experiments indicate that AMD473 is detected largely (>80% cross reaction) as the intrastrand AG adduct.

This work is supported by the Cancer Research Campaign, UK.

P11 DNA ALKYLATION AND INTERSTRAND CROSSLINKING BY TREOSULFAN.

C.C. O'Hare*[1], J.Baumgart[2] and J.A.Hartley[1]. [1]CRC Drug-DNA Interactions Research Group, Dept. Oncology UCL Medical School, 91 Riding House Street, London W1P 8BT, [2]Medac, Fehlandstrasse 3, D-20354 Hamburg, Germany.

The antitumour drug treosulfan (L-threitol 1,4-bismethane-sulfonate) is used clinically primarily in the treatment of advanced ovarian cancer. The lack of significant non-haematological toxicity suggests treosulfan as a candidate for high dose chemotherapy regimens with autologous stem cell reinfusion. Although structurally related to the alkylating agent busulphan, its mechanism of alkylation is distinct. It is a prodrug, converting non-enzymatically to L-diepoxybutane via the corresponding monoepoxide under physiological conditions. The present study demonstrates that conversion of treosulfan to epoxide species is required for cytotoxicity *in vitro*. Alkylation and interstrand crosslinking of plasmid DNA is observed following treosulfan treatment, again produced via the active epoxide species. Alkylation is sequence specific occurring at guanine bases with a preference for runs of contiguous guanines, as observed previously with alkylating agents such as nitrogen mustards. In treosulfan-treated human leukaemic K562 cells DNA crosslinks form slowly, reaching a peak at approximately 24 hours. Incubation of cells with the pre-formed epoxides shows faster and more efficient crosslinking. Using characterised paired human cell lines the sensitivity of cells to treosulfan was not determined by levels of either guanine-O6-alkyltransferase or glutathione.

P12 METABOLISM OF KW-2149: A NOVEL MITOMYCIN C ANALOGUE ACTIVATED IN SERUM.

S.R.McAdam*[1], J.A.Hartley [1], R.J.Knox[2] and J.R.Masters[3]. [1]CRC Drug-DNA Interactions Research Group, Dept. Oncology, UCL Medical School, 91 Riding House St, London W1P 8BT, [2]CRC Centre for Cancer Therapeutics, Institute of Cancer Research, Sutton, Surrey, SM2 5NG, [3]Inst. of Urology and Nephrology, 67 Riding House St. London W1P 7PN.

7-N-{{2-{[2-(-glutamylamino)-ethyl]}}mitomycin C (KW-2149) is a novel, highly water soluble analogue of mitomycin C (MMC) which is currently under investigation in clinical trials. KW-2149 demonstrates a similar spectrum of activity in a wide range of tumours but at a 10-100 fold lower concentration than MMC, is less myelosuppresive and lacks cross-resistance to MMC *in vitro* and *in vivo*. The cytotoxicity of KW-2149 in human bladder cancer cells (RT112) is dependent on a factor in serum as differences were found in activity between species of origin and batches of serum. The addition of serum to RPMI 1640 medium increased the rate of uptake into RT112 cells by 8-fold, cytotoxicity by >150-fold and DNA binding by 32-fold which indicates that KW-2149 is metabolised in serum-containing medium to a compound which enters cells and crosslinks DNA more rapidly than the parent drug. The major active metabolite of KW-2149 is thought to be the disulfide dimer M-18, however, the data shows that M-18 also requires reduction by thiols to efficiently crosslink naked DNA *in vitro* and also requires the presence of serum to kill cells in a colony forming assay. Experiments have been carried out to identify the factor in serum responsible for the metabolising activity of KW-2149. Purification of serum by ion-exchange chromatography has identified a fraction which converts KW-2149 to a toxic species. HPLC analysis, however, has shown that this fraction does not metabolise KW-2149 to M-18. Conversely, a fraction has also been identified which metabolises KW-2149 to M-18 *in vitro* but does not produce a cytotoxic species when added to cells in the presence of KW-2149.

P13 **VARIABILITY OF MITOMYCIN C ADSORPTION TO ACTIVATED CHARCOAL,**
I. A. Shah*[1], W. E. Lindup[1], P. McCulloch[2], [1]Department of Pharmacology and Therapeutics, University of Liverpool, PO Box 147, Liverpool, L69 3BX, UK. [2]Department of Surgery, University of Liverpool, PO Box 147, Liverpool, L69 3BX, UK.

Activated charcoal-adsorbed mitomycin C (AC-MMC) has been recommended for intraperitoneal use as adjuvant therapy for operable T3 gastric cancer. It has been suggested that the adsorption and release properties of the drug to this vehicle make it a safe and reliable slow-release preparation for clinical use[1,2], but the kinetics of the adsorption-desorption process have not been studied in detail. We tested the variability of MMC-AC adsorption. AC was ball milled and sieved to yield 4 fractions ranging from 53 to 180µm in diameter. Adsorption isotherms of each fraction were determined at 21°C and 37°C and expressed as Freundlich isotherms. The specific adsorption (Q; µg MMC/mg AC) was greater at 37°C than at 21°C but with up to 3-fold variation between samples analysed under identical conditions (21°C range: $Q = 72C^{0.22}$ to $32C^{0.08}$, 37°C range: $Q = 87C^{0.12}$ to $36C^{0.12}$). Specific adsorption of MMC was found not to be AC size dependent.

These results suggest that the adsorption characteristics of this drug-vehicle combination are more variable than expected, giving rise to potential concern about variations in effectiveness and toxicity. Further detailed evaluation of the release profile of the drug-charcoal combination, particularly *in vivo*, is required.

This work was supported by the Association of International Cancer Research. We would also like to thank Professors Akeo Hagiwara and Toshio Takahashi (Kyoto, Japan) for their help during this work.

[1]Hagiwara, A., Takahashi, T., Lee, R., Ueda, T., Takeda, M., Itoh, T. (1987) Anticancer Research, 7, 167-170.
[2]Hagiwara, A., Takahashi, T., Lee, R., Ueda, T., Takeda, M., Itoh, T. (1987) Anticancer Research, 6, 1161-1164.

P14 **A limited sampling strategy schedule method for determination of the AUC and the Cmax of JM216 in the multiple schedule administration.**
*F. Raynaud, W. Heybroek, I.R. Judson. CRC Centre for Cancer Therapeutics, the Institute of Cancer Research, 15 Cotswold road, Sutton, Surrey, UK. PK Plus, 35 Lower Green, Tewin, Welwyn, Herts.

JM216 is the first orally administrable platinum complex to reach Phase III clinical trials. In the first two phase I trials at the Royal Marsden hospital, it was shown that due to limited absorption no MTD could be reached on single dose administration and that a multiple dose schedule could overcome the saturability in absorption observed in the single dose study. The pharmacokinetic profiles showed that there was a good correlation between AUC for ultrafilterable platinum on day 1 and 5 and the myelosuppression grade (r=0.85). In order to lessen the sampling burden on the patients we present a limited sampling strategy that maintains sufficient predictability of both $C_{max}$ and 24h $AUC_{0-24h}$ for ultrafiltrable platinum. The method was validated against full profile method. Standard regression analysis of the variability of all predictor timepoints in data from day 1 and day 5 showed maximum predictability for $C_{max}$ and $t_{max}$ of 3hours and $AUC_{0-24h}$ at 3 and 12h post dose. Stepwise ananlysis of the groups of time points gave a suggested one point sampling at 4h for $AUC_{0-24h}$ (93% predictability). Addition of a sampling time at 6h post administration increased the predictability to 95%. A three point sampling at 2, 4, and 6h post sampling give a 93% predictability for the $C_{max}$. Mean percentage error and mean absolute percent errors are estimated at <8±6% and <20±4% respectively. This method should avoid the inconvenience of intensive pharmacokinetic sampling.

*This work was supported by the Cancer Research Campaign, UK.*

P15 **LINEARITY OF PHARMACOKINETICS AND TISSUE DISTRIBUTION OF C1311,**
C.R. Calabrese*, P.M. Loadman, M.C. Bibby[1] and J.A. Double[1], Clinical Oncology Unit, University of Bradford, BD7 1DP, UK - [1]Screening and Pharmacology Group, EORTC.

C1311 is a novel rationally designed anti-cancer agent which has shown promising activity *in vitro* and *in vivo* against both murine and human tumour xenograft colorectal cancer models. The aim of this study was to evaluate the linearity of plasma and tissue pharmacokinetics in non-tumour bearing mice prior to potential clinical application. In pharmacokinetic studies, female NMRI mice were treated at doses of 15, 50, 100 and 150 $mgkg^{-1}$ i.p (MTD). Plasma, liver, kidney and spleens were removed at various time points up to 24 hours after treatment and analysed for concentrations of C1311 using HPLC. Results for plasma and all tissues (below) suggest dose dependent pharmacokinetics. In each individual study, large differentials were observed between plasma and tissues with much higher concentrations observed in all of liver < kidney < spleen.

| | AUC (tissue:plasma ratio) (ND = Not Determined) | | | |
|---|---|---|---|---|
| Dose ($mgkg^{-1}$) | Plasma (µg.h/ml) | Liver (µg.h/g) | Kidney (µg.h/g) | Spleen (µg.h/g) |
| 15 | 1.06 (1) | 8.24 (8) | 47.8 (45) | 331 (312) |
| 50 | 7.30 (1) | 194 (28) | 560 (77) | 4218 (578) |
| 100 | 9.64 (1) | 238 (25) | ND | ND |
| 150 | 52.2 (1) | 871 (17) | 2421 (46) | 7153 (137) |

Tissue AUC's showed disproportional increases with dose for all of liver, kidney and spleen. The compartmentalisation of C1311 in murine whole blood was studied in order to assess how the blood distribution of drug may influence the pharmacokinetics, particularly in the highly perfused organs (liver and spleen). HPLC analysis was used to assess the percentage of applied drug present in plasma, blood cells, blood cell membranes and blood cell cytosol. Results showed approx. 20% in plasma with the remaining 80% split between cell cytosol (48%) and cell membranes (32%). Further experiments replacing drug containing plasma with drug-free plasma showed C1311 to re-equilibrate from the blood cell fraction indicating available drug. Therefore, the increase in plasma AUC is dose dependant though any haemolysis of blood will influence plasma levels due to the >4 fold concentration of C1311 in the blood cell fraction compared to plasma. Previous work has also shown C1311 to be highly protein bound within murine plasma (>98%). These data suggest that monitoring of whole blood in parallel to plasma pharmacokinetics would prove to be useful in a clinical setting.

(This work was supported by War On Cancer, Bradford, UK).

P16 **ASSESSMENT OF MURINE METABOLISM OF THE IMIDAZOACRIDINONE C1311,**
C.R. Calabrese*, P.M. Loadman, M.C. Bibby** and J.A. Double**, Clinical Oncology Unit, University of Bradford, Bradford, BD7 1DP, England - **Screening and Pharmacology Group, EORTC.

The imidazoacridinone C1311 is a novel rationally designed anti-cancer agent and has been identified as a probable candidate for clinical development by the EORTC. Studies in this laboratory have shown promising *in vivo* activity against murine colorectal cancers and human colon cancer xenografts.

The aim of this study was to assess the metabolism of C1311 in mice in an attempt to predict the metabolism and elimination of C1311 in a clinical setting and to identify any active or toxic metabolites. For pharmacokinetic studies, female NMRI mice were treated at 100 $mgkg^{-1}$ ip, with plasma and livers obtained at various time points up to 24 hours post treatment. Samples were extracted and analysed for parent compound using HPLC with fluorescence and diode array detection. Gradient HPLC analysis of the urine from mice ( 8 h sample) showed the presence of 8 minor metabolites with one major metabolite (M1) present in high concentrations. Subsequent studies using β-glucuronidase clearly showed M1 to be a glucuronide of C1311. Analysis of plasma and liver samples showed high levels of M1 in both plasma and liver. A further metabolite M2 was detected at high levels in the liver but not plasma or urine. M2 was non-fluorescent at the wavelengths used with λmaxima of 240 and 450nm (C1311 λmaxima = 250 and 420nm). AUC values for C1311 and metabolites were calculated to be: plasma 9.14 (C1311) 58.4 (M1) and <1µg.h/ml (M2) and liver 231 (C1311), 405 (M1) and 289µg.h/ml (M2) (using C1311 calibration values). *In vitro* incubations of parent compound with a variety of murine liver fractions (S9, cytosolic and microsomal) with NADH showed that conversion of parent compound to M2 occurred predominately in the cytosol. Total excreted drug (parent and metabolite) in urine was approximately 12% of the applied dose with parent compound and M1 comprising 5 and 95% of this respectively. Analysis of the collected faeces from the same experiment showed the presence of approximately 30% of the applied dose (94% C1311, 6% M1). M2 detected in liver samples has been isolated and purified and studies into its structure (using LC mass spectrometric analysis) and potential cytotoxic activity are currently ongoing. The high concentrations of glucuronide observed in plasma, liver, urine and faeces may be associated with potential enterohepatic recirculation of C1311 and therefore may be clinically important.

(This work was supported by War On Cancer, Bradford, UK).

### P17 POTENTIATION OF DDATHF BY PREVENTION OF HYPOXANTHINE SALVAGE

E.Marshman*, A.H.Calvert, D.R.Newell & N.J.Curtin, Cancer Research Unit, Medical School, University of Newcastle-upon-Tyne, NE2 4HH.

Hypoxanthine (HPX) abolishes the cytotoxicity of the antipurine 5,10-dideaza-5,6,7,8-tetrahydrofolate (DDATHF). The nucleoside transport inhibitor, dipyridamole (DP) has been found to potentiate methotrexate cytotoxicity by inhibition of thymidine and HPX uptake (T.C.K Chan & S.B. Howell, *Eur.J.Cancer*, **26**, 907, 1990). An earlier study from this laboratory demonstrated that DP prevented HPX rescue from DDATHF cytotoxicity in some cells but not others. The tissue-specificity of this observation has been extended to include further human tumour cell lines. DP did not have a synergistic effect with DDATHF, the combination producing similar $IC_{50}$ values compared with DDATHF alone (ranging from 4-590 nM). In all colon cancer (SW620, HCT116 and HT29) and leukaemic cell lines (CCRF-CEM, K562, MOLT4) DP did not inhibit HPX rescue. In addition, 2 ovarian cancer cell lines were also DP insensitive. Cell lines from other tissues produced variable responses, in 1 out of 3 breast carcinoma, 1 out of 3 lung carcinoma, and 2 out of 3 bladder carcinoma cell lines treated with DDATHF, DP effectively blocked HPX rescue; but the other cell lines were insensitive to DP. Thus the sensitivity of HPX rescue to DP has limited tissue specificity.

Sites of dose limiting toxicity of DDATHF are the bone marrow and gut mucosa. Two assays have been developed to investigate if the observation that DP does not block HPX rescue in leukaemia and colon cancer cells could be extended to their normal counterparts. A method for preparing rodent small intestine primary cultures has been established to study the response of intestinal epithelium. A granulocyte-macrophage colony-forming unit assay using human umbilical cord blood has been employed to investigate the effect of the combination of DDATHF, DP and HPX on haematopoietic progenitor cells. If these tissues are found to have DP insensitive HPX transport the combination of DDATHF, HPX and DP might be selectively cytotoxic to some tumours whilst not increasing dose-limiting toxicities.

### P18 A COMPARISON OF 5-FLUOROURACIL (5-FU) PHARMACOKINETICS IN WHOLE BLOOD, PLASMA AND RED BLOOD CELLS IN PATIENTS WITH COLORECTAL CANCER.

*Wiboon Wattanatorn[1]*, Howard L. McLeod[2], Fiona Macklon[2], Muriel Reid[2], Keith E. Kendle[1], and James Cassidy[2].* [1]School of Pharmacy, The Robert Gordon University, Schoolhill, Aberdeen AB10 1FR. [2]Department of Medicine & Therapeutics, Institute of Medical Sciences, University of Aberdeen, Aberdeen AB25 2ZD

**Introduction** The study of therapeutic drug monitoring (TDM) is of importance for anticancer therapy where most agents have a narrow therapeutic index. As measurement of drug concentrations in tumour tissue is not routinely possible, the choice of another surrogate biological matrix for analysis is necessary. Pharmacokinetic model selection is also important for accurate description and predictive ability.

**Aims** To compare whole blood, plasma and red blood cells 5-FU pharmacokinetics and to explore the feasibility of using intracellular concentrations of 5-FU for TDM.

**Methods** Five patients with colorectal cancer received folinic acid 200 mg/m$^2$ i.v. over 2 h followed by 5-FU 600 mg/m$^2$ i.v. bolus over 30 min, then 5-FU 600 mg/m$^2$ i.v. infusion over 22 h, administered on both days 1 and 2. This 48-hour cycle was repeated every 14 days. Blood samples (8 ml) were collected prior to the start of each cycle, and 5, 10, 20, 30 min, 1, 2, 16 h from the end of 5-FU bolus, then at the end of the 5-FU infusion, 20 and 40 min later to assess the decay phase. Concentrations of 5-fluorouracil in whole blood, plasma and red blood cells were determined by a previously described HPLC system (Wattanatorn et al, J Chromatogr, in press). ADAPT II was used for pharmacokinetic computations.

**Results** The concentrations of 5-FU in whole blood were 106-115% of simultaneous plasma concentrations (median = 112%), whilst packed red blood cell levels were 5-17% of plasma concentrations (median = 11%). The concentration-time profile of 5-FU was similar in the three matrices suggesting no benefit in monitoring intracellular concentrations. In addition, 5-FU is reported to be unstable in whole blood and red blood cell 5-FU concentrations were near the limit of detection (10 ng/ml), supporting the continued use of plasma as the preferred matrix for 5-FU TDM studies. Six pharmacokinetic models were fitted to the 5-FU individual data sets for determination of the best fit. The optimal model for whole blood and plasma data sets was a one compartment with both linear and non-linear elimination model; whilst a one compartment model with non-linear elimination provided the best fit for 5-FU in red blood cells. A two compartment model with non-linear elimination gave a similar degree of fit for plasma 5-FU as the one compartment model with both linear and non-linear elimination.

**Conclusion** These pharmacokinetic results provide the basis for further investigation into the ability to correlate 5-FU systemic exposure with clinical drug activity.

### P19 TITANOCENE DICHLORIDE: CIRCUMVENTION OF PLATINUM RESISTANCE, FIRST EVIDENCE OF *IN VITRO* DNA ADDUCTION AND POTENTIATION OF 5FU.

C. Christodoulou[1], A. Eliopoulos[1], T. Sheehan[2], L. Hodgkins[1],, L. Young[1], D. Ferry[1] and D. J. Kerr[1]. [1]CRC Institute for Cancer Studies, University of Birmingham, Birmingham, B15 2TA, [2]Regional Laboratory for Toxicology, City Hospital, Birmingham, B18 7QH, UK.

Titanocene dichloride (TD) was found to significantly overcome platinum resistance *in vitro*. The $IC_{50}$ values for the A2780 cells treated for 2 and 48 h with TD is 5.7 (±1.3) x $10^{-4}$ and 2.8 (±0.9) x $10^{-4}$ M respectively, whilst the 2780CP cell line is only 1.5-2-fold more resistant to TD (MTT assays). Similar results were obtained for CH1/CH1cisR cells. Moreover TD overcomes mutated p53-mediated platinum resistance. The $IC_{50}$ values for the mutated p53-expressing A2780 cells treated for 2 h with TD is 6.7 (±1.3) x $10^{-4}$ M. The cytotoxic activity of TD could be attributed to the formation of titanium-DNA adducts. A2780 cells were treated with 1 x $10^{-3}$ M TD for 2, 6 and 15 h. DNA was isolated from the cells and DNA-bound titanium was determined by flameless absorption spectrophotometry. Binding of titanium to DNA increased in a linear fashion propotional to the duration of exposure. We have also performed studies to determine whether there is synergy of TD and other cytotoxics *in vitro*. A2780 cells were treated for 2 h with various combinations of TD and 5FU, doxorubicin or cisplatin. Cytotoxicity was assessed 48 h later using MTT assays. Isobologram analysis suggests that combination of TD and 5FU is synergistic.

Supported by medac Hamburg, FRG.

### P20 FOOD REDUCES THE RATE BUT NOT THE EXTENT OF ABSORPTION OF THYMITAQ™ FOLLOWING ORAL ADMINISTRATION.

A.N.Hughes*[1], M.J.Griffin[1], A.H.Calvert[1] D.Simmons[1], I.Rafi[1], A.Johnston[2], N.J.Clendeninn[2], A.V.Boddy[1]. [1]Cancer Research Unit, University of Newcastle Upon Tyne. [2]Agouron Pharmaceuticals, San Diego, USA.

THYMITAQ™ is a non-classical thymidylate synthase (TS) inhibitor which has shown activity in Phase II clinical trials against head and neck cancer, hepatoma and pancreatic cancer. Due to its lipophilicity and lack of a glutamate moiety it does not require a specific cellular transport mechanism for uptake into cells and does not undergo conversion to polyglutamate forms. Initial studies using intravenous THYMITAQ™ infusions demonstrated that continuous administration over 5 days was needed to maintain adequate inhibition of TS. Further bioavailability studies on an oral preparation demonstrated that in fasted patients, absorption of THYMITAQ™ was rapid and almost complete (89%). In this study, we have sought to investigate the effect of food on the absorption of orally administered THYMITAQ™. Single oral doses of 200mg/m$^2$ (free base) were administered on 2 consecutive days. Patients were randomised and either fasted or fed a standard meal on Day 1 prior to drug administration. Day 2 was the opposite to Day 1. On the subsequent 5 days, patients received 200mg/m$^2$ THYMITAQ™ every 6 hours. The order of the study days was reversed on the 2nd cycle, 3 weeks later. Data from 8 patients were available for evaluation. In the fed state, the maximum plasma levels of THYMITAQ™ were lower (median 10.6µg/ml; range 7.6 to 15.2µg/ml compared to 20.4µg/ml; range 10.3 to 31.5µg/ml) and the time to maximum later (median 150min; range 45 to 185 min compared to 45min; range 30 to 60min) than in the fasted state. Nevertheless, the areas under the plasma concentration / time curves (AUCs) for the 2 doses were similar (fed 3.0mg/ml.min; range 2.0 to 4.8mg/ml.min compared to 3.3mg/ml.min; range 1.1 to 6.5mg/ml.min). The order of the study did not significantly affect the results. Since inhibition of TS is rapidly reversible, dosing of THYMITAQ™ may be preferable following the ingestion of food, as the slower absorption will result in a shorter duration of non-inhibitory concentrations prior to successive doses.

P21 SYNTHESIS AND ANTITUMOUR ACTIVITY OF CYCLOPENTA[*g*]QUINAZOLINE-BASED ANTIFOLATES, A NOVEL CLASS OF THYMIDYLATE SYNTHASE (TS) INHIBITORS: V. Bavetsias*[1], J. H. Marriott[1], C. Melin[1], R. Kimbell[1], F. T. Boyle[2] and A. L. Jackman[1], [1]CRC Centre for Cancer Therapeutics at The Institute of Cancer Research, Sutton, Surrey, UK; [2]ZENECA Pharmaceuticals, Alderley Park, Cheshire, UK.

Over the last two decades there has been extensive interest in the thymidylate synthase (TS) enzyme because of its critical role in DNA synthesis. Tomudex™, a polyglutamatable inhibitor of TS has recently completed phase III clinical trials with promising activity against colorectal cancer. In addition, other antifolates (*e.g.*, LY231514, BW1843U89, ZD9331, and AG337) are currently under clinical evaluation. Our present research program is mainly focused on the synthesis of cyclopenta[*g*]quinazoline-based, non-polyglutamatable inhibitors of TS, such as **1a-d**, that do not utilise the reduced-folate carrier (RFC) to enter cells. In the design of this series, the cyclopenta[*g*]quinazoline moiety was chosen because the conformational restriction introduced by the presence of the cyclopentane ring is believed to be favourable for binding to TS (F.T. Boyle *et. al.*, Proc. Am. Assoc. Cancer Res. 1996, **37**, Abstr. 2610).

Antifolates **1** were synthesised by coupling of 4-[*N*-((6RS)-2-methyl-4-oxo-3,4,7,8-tetrahydro-6H-cyclopenta[*g*]quinazolin-6-yl)-*N*-(prop-2-ynyl)amino]benzoic acid to the appropriate ligand via DEPC or PyBOP® carboxyl activation, followed by the removal of the protecting groups. This strategy required the development of synthetic routes to each individual glutamate-derived ligand. Antifolates of this type showed potent TS and cell growth inhibitory activities (*e.g.*, for **1 b**: $K_{iapp}$ = 0.78 nM, L1210 $IC_{50}$ =0.44±0.16 μM).

P22 *IN VITRO* ACTIVITY OF THE CYCLOPENTA[g]QUINAZOLINES, A NOVEL CLASS OF THYMIDYLATE SYNTHASE (TS) INHIBITORS. C.Melin*[1], R. Kimbell[1], V. Bavetsias[1], J. H. Marriott[1], F.T. Boyle[2] and A.L. Jackman.[1] [1]CRC Centre for Cancer Therapeutics, Institute of Cancer Research, Sutton, Surrey. [2]ZENECA Pharmaceuticals, Alderley Park, Cheshire.

The identification of intrinsic resistance mechanisms to inhibitors of TS which rely on the reduced folate/methotrexate carrier (RFC) for cellular transport and intracellular polyglutamation by folylpolyglutamate synthetase (FPGS), has led to the synthesis and development of a series of compounds with activity independent of both the RFC and FPGS. These compounds are acid based dipeptide analogues of the quinazoline-based TS inhibitor 2-desamino-2-methyl-N[10]-propargyl-5,8-dideazafolic acid (ICI198583), with a cyclopentene ring attached to the quinazoline moiety (see Bavetsias *et al*, accompanying abstract). Two such examples are CB300638 (L-glu-γ-D-glu) and CB300907 (L-glu-γ-D-glu, where the α-carboxyl group of the second amino acid is replaced with a tetrazole ring). Both are potent inhibitors of TS (TS Kiapp = 0.2nM and 0.36nM) with similar in *vitro* cytotoxic potency in the L1210 ($IC_{50}$ 1.1 ± 0.54μM and 1.7 ± 0.54μM) and W1L2 ($IC_{50}$ 0.48 ± 0.2μM and 1.3 ± 0.77μM) cell lines. At least a 25-fold decrease in cytotoxic potency in W1L2 cells in the presence of thymidine confirms TS as the sole locus of action. The absence of cross-resistance in the RFC-defective L1210:1565 cell line suggests that these compounds are not dependent on the RFC for cellular uptake. These compounds also showed poor substrate affinities for the RFC when assayed in competition with [$^3$H]methotrexate (Ki in L1210 = > 250μM and 106μM respectively; methotrexate = 5μM). No change in growth inhibitory activity was seen in the L1210:R$^{D1694}$ cell line, which shows resistance to polyglutamatable TS inhibitors such as ZD1694 (Tomudex™). Both compounds showed activity in the human colon tumour cell lines HT29 (CB300638 $IC_{50}$ 0.8μM; CB300907 $IC_{50}$ 2.2μM), and SW480 (CB300638 $IC_{50}$ 0.92μM; CB300907 $IC_{50}$ 2.3μM). Compounds of the non-RFC, non-FPGS class as exemplified above may show a different and/or wider spectrum of antitumour activity *in vivo* compared to other classes of TS inhibitors. These compounds are currently undergoing further investigation as potential antitumour agents.

P23 COMBINATION STUDIES WITH TOMUDEX™ AND SN-38 (CPT-11) IN A PANEL OF HUMAN COLON TUMOUR CELL LINES. Rosemary Kimbell* and Ann L. Jackman. The Institute of Cancer Research, Sutton, Surrey.

ZD1694, (Tomudex™, ZENECA Pharmaceuticals), a folate-based thymidylate synthase (TS) inhibitor, has recently been registered in several countries for first-line therapy in advanced colorectal cancer. Advantages over 5-fluorouracil (FU), which has been the main treatment for several decades, include significantly decreased leucopenia and mucositis, and a convenient bolus 3-weekly administration schedule. SN-38, a DNA topoisomerase I inhibitor, is the active metabolite of CPT-11 (Rhône-Poulenc Rorer), which is registered in some countries for the treatment of FU-resistant colorectal cancer. Both ZD1694 and SN-38 are potent inhibitors of cell growth ($IC_{50}$s 1-5nM) in a panel of six human colon tumour cell lines (HT29, MaWi, LoVo, HCT8, SW620 and SW480), and SN-38 retained activity in a panel of cell lines with various mechanisms of resistance to antifolates (eg. elevated TS, defective transport or polyglutamation). Their different mechanisms of action suggest there may be a rationale for their combination in the clinic. *In vitro* combination studies have been carried out using a range of dosage and scheduling regimens in the panel of colon tumour lines. Dialysed serum was used to eliminate any potential protective effects of thymidine during exposure to ZD1694. Growth inhibitory activity was determined by MTT assay after five days. Co-incubation with equitoxic doses of both drugs for five days, or for twenty four hours with drug-free medium for the remaining period, showed additivity or antagonism (as measured by Chou & Chou computerised analysis, ©BIOSOFT), giving combination index (CI) values at fraction affected (Fa) 0.5 of 1.0 to 1.8. Sequential exposures, such as twenty four hours to one drug followed by four days to the second drug, with or without removal of the first drug, gave similar results, with slightly more antagonism if the initial exposure was to ZD1694. Manual isobologram construction confirmed these findings. Non-equitoxic combinations (with up to a hundred-fold dose reduction of either drug), were also additive or antagonistic. However, if exposure was reduced to four hours SN-38 followed by four hours ZD1694, at approximately equitoxic doses, some synergy was observed, with the reverse sequence again giving additivity or antagonism (MaWi, CI @ Fa 0.5, SN-38 dosed first, 0.75, ZD1694 first, 1.25; HCT8 0.8 and 1.0; HT29 0.8 and 0.95 respectively). The effects of combinations of ZD1694 and SN-38 *in vitro* are therefore highly dose, time and sequence dependent, and this should be taken into consideration when extending studies to the clinical situation. *Supported by the* **CRC**.

P24 EFFECTS OF LEUCOVORIN (LV) AND ORAL FOLIC ACID (FA) ON THE TOXICITY AND ANTITUMOUR ACTIVITY OF THE THYMIDYLATE SYNTHASE (TS) INHIBITORS, TOMUDEX™ (ZD1694) AND ZD9331. C. Rees*, R. Kimbell, M. Valenti, L. Brunton, D. Farrugia and A.L. Jackman. The Institute of Cancer Research, Sutton, Surrey.

The toxicity of antifolates such as methotrexate or lometrexol may be controlled by the administration of LV or FA. ZD1694, a polyglutamatable TS inhibitor, is a highly specific TS inhibitor now registered for the treatment of advanced colorectal cancer. The antiproliferative effects of ZD1694 are prevented by co-administration of LV (through competition for cellular uptake via the reduced-folate carrier and polyglutamation i.e. the active tri-pentaglutamate drug species are not formed). 1843U89 is a TS inhibitor, (in Phase I study) that is polyglutamated to the diglutamate only. The gut toxicity, but not the antitumour activity, of this compound in mice and dogs is prevented by co-administration of p.o. FA (Smith *et al*, Cancer Res. Dec. 95). This effect is hypothesised to be due to the presence of a low Km transporter for FA in the gut. ZD9331 is a novel, non-polyglutamatable TS inhibitor that is in Phase I clinical study. We have examined a) the effect of LV and FA on the cytotoxic activity of ZD1694 and ZD9331 in 6 human tumour colon cell lines, b) the effect of i.p. LV and p.o. FA on the antitumour activity and/or toxicity of ZD1694, c) the effect of LV or FA on the toxicity of ZD9331. The colon tumour cell lines (HT29, LoVo, SW480, SW620, Mawi and HCT-8) with 25nM LV as the folate source, were co-incubated (5 days) with either ZD1694 ($IC_{50}$ 3-8nM) or ZD9331 ($IC_{50}$ 10-30nM) and 5μM LV or 25μM FA. LV competes with both drugs for initial cellular uptake, but, because it also competes with ZD1694 for polyglutamation, increased its $IC_{50}$ 200-fold compared with 3-fold for ZD9331. 25μM FA increased the $IC_{50}$ of ZD1694 up to 9-fold, which is probably due to a rise in the intracellular reduced-folate pool and competition for polyglutamation. The same concentration of FA had little effect on ZD9331 cytotoxicity. Balb/c mice were used as a model for drug-induced gut toxicity (weight loss). The toxicity of ZD1694 (10mg/kg i.p. daily x 5 days) was completely prevented by co-administration of LV (20mg/kg/twice daily i.p. x 8 days), but that of ZD9331 (100mg/kg/d x 7days s.c. infusion) was only partially prevented (100mg/kg i.p. twice daily x 7days); 13 ± 3.1% weight loss by day 8 compared with 21 ± 2.6% weight loss for ZD9331 alone. FA (300mg/kg daily p.o.) prevented the weight loss induced by ZD1694 but not that by ZD9331. The L5178Y antitumour activity of ZD1694 (DBA2 mice) is also prevented by p.o. FA (100 or 300mg/kg daily). Although, these data do not currently suggest a role for the co-administration of FA with either ZD1694 or ZD9331, the effect of LV on the antitumour activity of ZD9331 will be examined. *Funded by the CRC and Zeneca Pharmaceuticals.*

P25 ESTIMATION OF 2'-DEOXYURIDINE (dUrd) IN HUMAN PLASMA BY HPLC WITH FLUORIMETRIC DETECTION; A PHARMACODYNAMIC MARKER FOR THYMIDYLATE SYNTHASE (TS) INHIBITION.

F. Mitchell*, D. Farrugia, C. Rees, D. Cunningham, I. Judson, and A.L.Jackman. The CRC Centre for Cancer Therapeutics, Institute of Cancer Research and Royal Marsden Trust, Sutton, Surrey.

ZD1694 (Tomudex™) is a polyglutamatable, highly specific, folate-based TS inhibitor used in the treatment of advanced colorectal cancer and ZD9331 is a non-polyglutamatable TS inhibitor in Phase I study. TS inhibitors such as 5-fluorouracil, CB3717, ZD1694 and ZD9331 cause an increase in the level of intracellular dUMP which can be dephosphorylated to dUrd and transported out of the cell. Studies in both mice and/or humans have demonstrated a rise in plasma dUrd after treatment with the TS inhibitors, CB3717, AG337, and ZD1694. This suggests that plasma dUrd may be a useful pharmacodynamic marker of TS inhibition (in proliferating tissues). Plasma dUrd levels, together with pharmacokinetic and clinical data, contributed towards the decision to initiate a 5-day continuous infusion Phase I study of AG337 (Rafi *et al*, Clin. Cancer Res, 1996). HPLC methods used for the measurement of plasma dUrd have previously relied on UV absorbance at 254/280nm (non-specific wavelength). The presence of interfering peaks requires sequential chromatographic steps. This led us to develop an improved, sensitive HPLC method for the detection of plasma dUrd. Following solid-phase extraction of dUrd from plasma (to which 10nmoles of 5-CldUrd are added as an internal standard), a derivitisation reaction is carried out using the fluoresence tagging reagent, 4-bromomethyl-6,7-dimethoxycoumarin (BrMdmc). The derivatised dUrd is separated on an isocratic reversed-phase system with a run-time of 40min. (4.6x250mm SUPELCOSIL LC-18 5μm column; mobile phase, 16% acetonitrile in 0.05% v/v acetic acid), at 45°C. The high specificity of the derivitisation reagent (only reacts with carboxylic acids, fatty acids, pyrimidine compounds) reduces the number and size of interfering peaks, which means that only a single HPLC step is required. dUrd is quantified with reference to a 4-point standard curve. The limit of detection is approximately 0.05pmole on-column and the limit of quantitation in a 1ml plasma sample is ~1nM. The inter- and intra-batch coefficient of variation are 6% and 8% respectively. The dUrd levels in the plasma of five patients receiving a standard dose of 3mg/m$^2$ of Tomudex have been analysed. The pre-treatment level was ~0.05μM which rose 1.5 to 6-fold 24h after treatment although this had returned to (or fallen below) pre-treatment levels 5 days later. Rises in plasma dUrd have also been seen in some patients in the Phase I clinical study of ZD9331 (5-day continuous infusion).

P26 COMPONENTS OF THE URACIL MISINCORPORATION PATHWAY; THEIR INFLUENCE ON CELLULAR RESPONSE TO THYMIDYLATE SYNTHASE (TS) INHIBITORS.

S. D. Brown*, A. Hardcastle, R. D. Ladner[1], and G. W. Aherne. CRC Centre for Cancer Therapeutics, Institute of Cancer Research, Sutton, Surrey, UK, and [1]Univ. Med. Dent. of NJ, Stratford, NJ08084.

TS plays a critical role in DNA replication since it provides dTTP and is involved in balancing deoxynucleotide precursor pools during DNA synthesis. Inhibition of TS, in addition to blocking dTTP formation, greatly expands the dUMP pool which in turn may elevate dUTP pools. dUTP misincorporation and uracil misrepair have been implicated as important events accompanying prolonged TS inhibition. However, the enzymes of this uracil misincorporation pathway remain to be identified as critical components influencing sensitivity to TS inhibition. We have addressed the relationship between key enzymes involved in dUTP misincorporation and cellular sensitivity to TS inhibition in 5 human cell lines (1 lymphoblastoid, 3 non-small cell and 1 small cell lung carcinoma). Sensitivity (5 day MTT assay) to the growth inhibitory effects of the non polyglutamatable, quinazoline antifolate, specific TS inhibitor ZD9331, were found to differ up to 9 fold ($IC_{50}$ range: 0.046μM- 0.005μM). This variation in sensitivity did not correlate with TS protein expression determined using Western immunoblot analysis. Using sensitive radioimmunoassay procedures, we have measured dTTP and dUTP pools following a 24hr exposure to 1μM ZD9331. The amount of dUTP formed was inversely related to the pyrophophatase activity of dUTPase (0.001μM-0.018μM/min/mg protein). The dUTP/TTP ratios after this treatment ranged from 0.4-11.8 (control cells <0.15). The most sensitive cell line CORL23 had the highest dUTP/dTTP ratio (11.8) after this treatment. This compared to a dUTP/dTTP ratio of only 0.4 for one of the more resistant cell lines (MOR). Despite having a high dUTP/dTTP ratio of 7.9, the A549 cell line was the most resistant to ZD9331. Uracil-DNA glycosylase enzyme activity in the cell lines was measured. The A549 cell line had a significantly (p=0.0001) higher level of this DNA repair enzyme activity (932pmol uracil/min/mg protein) than the other cell lines (average activity 238 ±153pmol uracil/min/mg protein). It appears that cellular response to TS inhibition maybe effected by the balance between factors influencing the dUTP/dTTP ratio e.g dUTPase, and those enzymes involved in repairing DNA. Further work will address the extent of DNA damage induced by specific inhibitors of TS and the expression of proteins involved in the recognition and initiation of events post DNA damage.

P27 IN VIVO PHARMACOKINETIC AND PHARMACODYNAMIC STUDIES OF TWO SPECIFIC THYMIDYLATE SYNTHASE (TS) INHIBITORS.

A.Hardcastle,* D. Dobinson, D. Farrugia, A.L. Jackman and G.W. Aherne, CRC Centre for Cancer Therapeutics, Inst. of Cancer Research, Sutton, Surrey SM2 5NG.

TS catalyses the reductive methylation of dUMP to TMP, which is converted to TTP, the only dNTP required exclusively for DNA synthesis. ZD1694 (Tomudex™, raltitrexed) and ZD9331 are quinazoline antifolates which specifically inhibit this enzyme. Both compounds are transported into the cell by the reduced folate carrier (RFC) but unlike Tomudex, ZD9331 does not rely on polyglutamation for high potency. We have previously shown by the measurement of TTP and dUMP pools *in vitro* that TS inhibition is rapidly reversed when non-polyglutamatable compounds are removed from the medium. In contrast TS inhibition is prolonged following exposure of cells to ZD1694 even when drug is removed from the medium (Aherne *et al.*, 1996, Biochem Pharmacol. 51, 1293; Aherne *et al.*, 1996, Annals of Oncology 7, Suppl.1, 89). These data are consistent with rapid efflux of ZD9331 from cells compared with the intracellular retention of potent polyglutamated species after exposure to ZD1694. The relationship between plasma and tumour drug levels and perturbations in TTP pools has now been studied *in vivo*. In order to overcome the problem of thymidine salvage from plasma in mice, the salvage incompetent L5178Y thymidine kinase negative (TK-/-) mouse lymphoma was used. This tumour is cured by both ZD1694 (1x10mg/kg ip) and ZD9331 (1x50mg/kg ip and 3mg/kg sc 24h infusion). L5178Y TK-/- cells (2.5 x10$^5$) were implanted sc into a hind leg of female DBA2 mice Drugs were administered 4-7 days later (tumour size ≅8mm) to groups of 3-6 mice. Plasma and tumour tissue were obtained at intervals for measurement of drug (RIA for ZD1694 and ELISA for ZD9331) and tumour TTP pools (RIA). For ZD9331 (50mg/kg ip) plasma drug levels at 4h (1.82±0.6μM) exceeded those in tumour 2 fold but by 24h tumour levels were approx. 5 times higher than those in plasma (0.02-0.04μM). Tumour TTP pools were significantly depleted compared to control animals (56%) at 4h (p=0.01) and 6h (p=0.004) but not at 16h or 24h. During the ZD9331 infusion (3mg/kg sc 24h) plasma drug levels reached a plateau of 0.4-0.8μM and tumour drug concentrations were 1-2 fold higher. TTP pools were significantly depleted at 4h (44% controls p=0.003) and at the end of infusion (27% controls p=0.0001). Total ZD1694 levels in tumour were 50-60 times those in plasma 24 and 48 hours (0.02μM and 0.004μM respectively) after ip injection (10 mg/kg). Significant depletion of TTP was observed at 4h (16% controls p=0.009) and at 24h (68% controls p=0.002) but not at 48h (105% controls). These on-going pk/pd studies in L5178Y TK-/- and other tumour bearing animals may provide important information for the design of improved clinical schedules of administration.

P28 **The Induction of Apoptosis, Cell cycle arrests and p53 protein accumulation in ovarian cancer cell lines by Camptothecin and its water soluble analogues.**

AC McDonald* and R Brown. CRC Dept of Medical Oncology, University of Glasgow, Beatson Laboratories, Garscube Estate, Glasgow G61 1BD

Given the activity of camptothecin analogues in platinum refractory ovarian cancer we have evaluated the *in vitro* effects of such compounds in an ovarian cancer cell line model system. The A2780 cell line exhibits functional p53 dependent G1 arrest following 2 Gy ionising radiation (IR). Treatment of A2780 with Camptothecin and three water-soluble analogues, topotecan, GI147211 and SN-38, (the active metabolite of CPT-11), induces cellular accumulation of p53 protein, maximal 24 hours following a 1 hour exposure. Treatment of A2780 with 2 x $ID_{80}$ concentrations of camptothecin and these analogues induces apoptosis assessed by TUNEL staining / FACS analysis, maximal 72-96 hours post treatment. Similar exposure of A2780 to $ID_{80}$ concentrations of each drug induces cell cycle change, with a 6-12 fold accumulation of cells in G2 phase 24 hours after drug exposure. No evidence G1 / S delay is observed.

We have previously shown mutant p53 (codon 143, val to ala) transfectants (A2780/mp53) of A2780 lose IR induced G1 arrest, retained by vector alone controls (A2780/v) (McIlwrath et al Cancer Res. 1994; 54:3718-22). A2780/mp53 and A2780/v express topoisomerase 1 equally, assessed by western immunoblotting. Using clonogenic assay, A2780/mp53 acquires 2-3 fold resistance to a number of agents including cisplatin and adriamycin (Vasey et al Molecular Pharmacology 1996, in press). These cells are not cross resistant to camptothecin, topotecan or SN-38 but are 2.3 fold resistant to GI147211. Cell cycle studies show each agent capable of inducing G2 arrest at 24 hours in both A2780/mp53 and A2780/v.

Camptothecin and its analogues induce apoptosis and cellular accumulation of p53 in A2780. They also induce a G2 arrest, but both cell cycle perturbation and cell survival does not appear dependent on wt p53.

Topotecan kindly provided by Smithkline Beecham, GI147211 by GlaxoWellcome and SN-38 by Rhone-Poulenc Rorer.

P29 **CELLULAR RESPONSES TO TOPOISOMERASE I (TOPO) INHIBITORS AND PATHWAYS OF CELL DEATH.** J.S. Macpherson, J. Cummings, E. Miller and J.F. Smyth, ICRF Medical Oncology Unit, Western General Hospital, Edinburgh, EH4 2XU.

Sensitivity of 5 unrelated human cell lines (HL60, A2780 and 2780$^{AD}$, HT-29 and NX002) to the novel topo I inhibitor (NU/ICRF 505) does not correlate to topo I expression but rather to the presence of wild type p53 and a high Bax/Bcl-2 ratio. To further define factors dictating chemosensitivity to topo I poisons, studies have been extended to include the classic inhibitor camptothecin (CPT) and to follow cellular responses after drug incubations at $IC_{50}$ concentrations. A similar pattern of chemosensitivity to NU/ICRF 505 and a lack of a correlation to topo I protein levels were observed with CPT. Treatment of more sensitive wild type p53 cells (A2780) with NU/ICRF 505 (10 μM) resulted in a >10-fold elevation in p53 protein at 24 hr; a potent induction of p21 with peak levels at 36-48 hr and a 3-fold increase in Bax protein after 48 hr. These responses corresponded to cells both accumulating in G1/S and entering into apoptosis 24-48 hr after drug treatment. A more sustained stimulation of p53 occurred after CPT treatment (4 x $10^{-9}$ M) with peak protein levels detected at 48 hr (7-fold induction), as well as increased p21 at 48 hr. However, no significant perturbation in cell cycle distribution was evident. Treatment of less sensitive mutant p53 cells (HT-29, 4.6-fold resistant to NU/ICRF 505 and 3.2-fold resistant to CPT) with both compounds failed to induce p53, p21 or Bax. NU/ICRF 505 had no effect on cell cycle distribution whereas CPT produced a marked G2/M accumulation. These data are consistent with topo inhibitors killing cells through both more sensitive p53 dependent pathways and less sensitive non-p53 dependent pathways. In addition, the two inhibitors can differentially affect the cell cycle whilst essentially eliciting the same cellular responses.

P30 **IN VITRO CELL CYCLE EFFECTS AND GROWTH INHIBITION OF THE HT29 HUMAN COLON CANCER CELL LINE BY [D-ARG$^{1}$,D-TRP$^{5,7,9}$,LEU$^{11}$]-SUBSTANCE P.** D.A. Jones*, E.P. Miller and J.F. Smyth. Imperial Cancer Research Fund, Medical Oncology Unit, Western General Hospital, Edinburgh EH4 2XU.

[D-Arg$^{1}$,D-Trp$^{5,7,9}$,Leu$^{11}$]-Substance P belongs to a class of compounds termed broad spectrum neuropeptide antagonists possessing the unusual ability of being able to inhibit simultaneously the action of several calcium-mobilising neuropeptides such as bombesin, bradykinin and gastrin. These peptides have been shown to act as growth factors in several cancer types including small cell lung and colorectal cancer. In vitro, the HT29 cell line is growth stimulated by foetal calf serum (FCS) which is known to contain a variety of calcium-mobilising agents. This research has investigated **(i)** the ability of [D-Arg$^{1}$,D-Trp$^{5,7,9}$,Leu$^{11}$]-substance P to inhibit the increases in $[Ca^{2+}]_i$ in response to FCS; **(ii)** the anti-proliferative effect of [D-Arg$^{1}$,D-Trp$^{5,7,9}$,Leu$^{11}$]-substance P on HT29 cells grown in the presence of FCS; **(iii)** the effect of the antagonist on the cell cycle of the HT29 cells in order to define whether the observed growth inhibition is due to decreased cell proliferation and/or induction of cell death.
**Methodology** : $[Ca^{2+}]_i$ mobilisation was measured using Fura-2AM loaded cells and employing a ratiometric analysis of the change in fluorescence at 510nm after excitation at 340/380nm. Dose-response curves for [D-Arg$^{1}$,D-Trp$^{5,7,9}$,Leu$^{11}$]-substance P against HT29 cells were performed in quadruplicate in media containing 5% FCS. Cell cycle analysis and BRDU pulse-labelling was performed 24 hours post antagonist addition on a Becton-Dickinson FACScan using CellFit software.
**Results** : **(i)** In control samples addition of 1% FCS caused a rise in $[Ca^{2+}]_i$ of 311nM (+/-48)whilst in the presence of 20μM [D-Arg$^{1}$,D-Trp$^{5,7,9}$,Leu$^{11}$]-Substance P a rise of only 22nM (+/-6) was observed. The competitive nature of this inhibition was confirmed by the addition of 10% FCS which was able to reverse the effect. **(ii)** [D-Arg$^{1}$,D-Trp$^{5,7,9}$,Leu$^{11}$]-substance P inhibited the growth of the HT29 cells in a dose-dependent manner exhibiting an $IC_{50}$ value of 20μM. **(iii)** Following treatment with 20μM antagonist the proportion of cells in all phases of the cell cycle were significantly affected ($p$ <0.0001). BRDU pulse-labelling of the cells demonstrated that the treated cells were progressing through the cell cycle at a rate 2.5-fold slower than the controls. No evidence of induction of apoptosis was observed on the FACScan.
**Conclusions** : The data presented indicate that **(i)** [D-Arg$^{1}$,D-Trp$^{5,7,9}$,Leu$^{11}$]-substance P acts as a competitive inhibitor of calcium-mobilising mitogens present in FCS and for the first time **(ii)** at the $IC_{50}$ the anti-proliferative activity of these compounds is due to a prolongation of the cell cycle rather than apoptosis.

P31 **[DArg$^{1}$, DTrp$^{5,7,9}$, Leu$^{11}$]Substance P coordinately and reversibly inhibits neuropeptide induced signal transduction pathways in Swiss 3T3 cells,** M.J. Seckl* and E. Rozengurt, ICRF, 44 Lincoln's Inn Fields, London WC2 3PX.

The development of novel therapies for small cell lung cancer (SCLC) is urgently required. Since the growth of this tumour is driven by multiple neuropeptides including bombesin and vasopressin, a broad spectrum neuropeptide inhibitor might be a useful therapeutic approach. Indeed, the substance P (SP) analogues, [DArg$^{1}$, DPhe$^{5}$, DTrp$^{7,9}$, Leu$^{11}$]SP and [Arg$^{6}$, DTrp$^{7,9}$, MePhe$^{8}$]SP (6-11) inhibit the action of a broad range of neuropeptides including bombesin and vasopressin in Swiss 3T3 cells and inhibit SCLC cell proliferation in liquid culture, soft agar and as xenografts in nude mice. Despite their intriguing biological effects and potential importance as antiproliferative agents, the mechanism of action of SP analogues as broad spectrum inhibitors of neuropeptide-mediated signal transduction remains unclear.

Here we show that the novel SP analogue, [DArg$^{1}$, DTrp$^{5,7,9}$, Leu$^{11}$]SP like [DArg$^{1}$, DPhe$^{5}$, DTrp$^{7,9}$, Leu$^{11}$]SP inhibited DNA synthesis induced by bombesin, vasopressin and bradykinin but did not interfere with the mitogenic response induced by other growth promoting agents in Swiss 3T3 cells. Both SP analogues reversibly inhibited bombesin stimulated mitogenesis but [DArg$^{1}$, DTrp$^{5,7,9}$, Leu$^{11}$]SP was more potent. The new SP analogue coordinately and reversibly inhibited bombesin induced $Ca^{2+}$ mobilization, protein kinase C and mitogen activated protein kinase (MAPK) activation. In addition, [DArg$^{1}$, DTrp$^{5,7,9}$, Leu$^{11}$]SP reversibly inhibited bombesin induced tyrosine phosphorylation. [DArg$^{1}$, DTrp$^{5,7,9}$, Leu$^{11}$]SP also reversibly and coordinately inhibited vasopressin induced signal transduction and DNA synthesis. Surprisingly, deletion of the terminal Leu of [DArg$^{1}$, DPhe$^{5}$, DTrp$^{7,9}$, Leu$^{11}$]SP to yield [DArg$^{1}$, DPhe$^{5}$, DTrp$^{7,9}$]SP$^{1-10}$ resulted in a selective loss of inhibitory activity of this analogue against bombesin- but not vasopressin-stimulated DNA synthesis, $Ca^{2+}$ mobilization and MAPK activation. Collectively, these results suggest that SP analogues act at the receptor level to coordinately and reversibly antagonize bombesin or vasopressin induced signal transduction in Swiss 3T3 cells.

P32 **GROWTH INHIBITORY EFFECTS OF THE SYNTHETIC RETINOID CD437 AGAINST OVARIAN CARCINOMA MODELS IN VITRO AND IN VIVO**

SP Langdon[1], GJ Rabiasz[1], AA Ritchie[1], U Reichert[2], JF Smyth[1] and WR Miller[1].

[1]ICRF Medical Oncology Unit, Western General Hospital, Crewe Road, Edinburgh,

[2]CIRD Galderma, Valbonne, France

Retinoids are being increasingly considered as cancer therapeutic and chemo-prevention agents. Retinoid signal transduction is mediated by a network of six interacting nuclear receptors that fall into two classes, the RARs (α,β and γ) and the RXRs (α, β and γ). CD437 (6-[3-(1-adamantyl)-4-hydroxyphenyl]-2-naphthalene carboxylic acid is a relatively selective activator for RAR-γ and has recently been demonstrated to regulate p21$^{WAF1/CIP1}$ expression in breast carcinoma cells in a p53-independent manner (Shao et al Oncogene, 11, 493-504, 1995).

In this study we have evaluated the activity of CD437 against 4 human ovarian carcinoma cell lines : PE01, PE04 (a Pt-resistant *in vivo* derived counterpart of PE01), PEC1$^{CDDP}$ (a Pt-resistant *in vitro* derived model of PE01) and PE014. Growth inhibition against these 4 lines was investigated after 6 days exposure and assessment was by cell number. $IC_{50}$ values were 0.1, 0.14, 0.07 and 0.17 μM for PE01, PE04, PE01$^{CDDP}$ and PE014 respectively. These values indicate that this retinoid is effective at submicromolar concentrations. Cisplatin-resistant cell lines were as responsive to this retinoid as cisplatin-sensitive lines indicating potential activity in resistant disease.

CD437 was evaluated against the PE04 xenograft grown in nude mice. The agent was tested at doses of 20 mg/kg/day (given on days 0-4) and 10 mg/kg/day (days 0-4, 7-11) and was given by either intraperitoneal delivery or oral administration. Significant growth inhibition (p<0.05) was obtained for both doses and by both routes.

These data provide further support for the view that retinoids may have value for the treatment of ovarian cancer.

P33 DEVELOPMENT OF AN INTERNAL STANDARD FOR QUANTITATIVE PCR (QPCR) MEASUREMENT OF DNA DAMAGE AND REPAIR IN SINGLE COPY GENES.
J.P. Bingham*, J.A. Hartley, and K.A. Grimaldi. CRC Drug-DNA Interactions Research Group. Dept. Oncology, UCL Medical School, 91 Riding House St. London W1P 8BT

QPCR has previously been shown to be a highly sensitive method of detection of gene specific DNA damage and repair. It depends upon blockage of *taq* polymerase by DNA adducts and damage can be quantified since the amount of PCR product will be directly proportional to the amount of undamaged template. It is critical to control the amount of DNA in the reaction since any variation in DNA extraction from treated cells will result in assay imprecision. U.V. determination of DNA concentration lacks adequate precision and is laborious when applied to many small samples. The use of a fixed number of cells for the treatment followed by a parallel DNA extraction using a simple, single-tube protocol is quicker and more reproducible but the results can still be influenced by variation in the DNA extractions. To reduce this variation we have investigated the use of an internal standard for QPCR. Human U937 cells were treated with a DNA damaging agent and then "spiked" with a fixed number of mouse L1210 cells. DNA was extracted from the mix of cells and subjected to two separate QPCR's under appropriate conditions for each primer pair. A 523 bp fragment of the human *N-ras* gene was the target for the DNA damage studies and a 950 bp fragment of the mouse HPRT gene as the internal standard. Conditions were optimised to ensure no cross-reactivity between the two target species. The results showed that the amount of amplified HPRT product generated was directly proportional to the amount of starting DNA while the amount of *N-ras* product depended also on the extent of damage. Thus the mouse HPRT template acts as an undamaged internal control which can be used as a baseline to account for any variation in DNA extraction when measuring DNA damage in the *N-ras* gene.

P34 **SENSITIVITY TO NITROGEN MUSTARD IN *SACCHAROMYCES CEREVISIAE* DEFICIENT IN DNA REPAIR**
Gill, R.D.*[1], Waters, R.[2], Hartley, J.A.[1] [1]CRC Drug-DNA Interactions Research Group, Department of Oncology, UCL Medical School, 91 Riding House Street, London W1P 8BT, [2]School of Biological Sciences, University of Wales, Singleton Park, Swansea SA2 8PP

Nitrogen mustard (HN2) is a potent chemotherapeutic drug which is a bifunctional alkylating agent capable of forming both monoadducts and interstrand crosslinks in DNA. In order to examine the mechanisms of repair of damage induced by this drug, *Saccharomyces cerevisiae* strains deficient in a single DNA repair enzyme have been studied. The strains include representatives from the 3 major epistasis groups of DNA repair in yeast, comprising mutants deficient in nucleotide excision repair, post-replication repair, and recombination repair, as well as representatives deficient in base excision repair. Comparison of the toxicity of HN2 over the dose range 1-1000μM has revealed a wide range of responses. The most sensitive strains tested to date are from the nucleotide excision repair epistasis group, *rad*14 and *rad*2. *RAD*14 codes for a protein involved in initial damage recognition while *RAD*2 codes for an endonuclease that cleaves on the 3′ side of a damaged site in DNA in the nucleotide repair pathway. The sensitivity exhibited by these strains is approximately $10^5$-$10^7$-fold greater than the parental strain. Cleavage at the 5′ site of the damage is accomplished by an enzyme complex of *RAD*1 and *RAD*10 proteins, but the strains deficient in either of these enzymes exhibit much lower sensitivity to HN2 treatment (10-100-fold). A *rad*3 strain had a low sensitivity to HN2. The *RAD* 3 gene codes for an essential activity that is part of the yeast transcription apparatus as well as nucleotide excision repair. Each of the other DNA repair pathways have at least one mutant strain that exhibits marked sensitivity to nitrogen mustard. A *rad* 9 strain, from the post-replication/error-prone repair epistasis group, and *rad*54 and *rad*52 strains, from the recombination repair epistasis group, are each 10-100-fold more sensitive than the parental strain. Finally, a representative of the base excision repair group, lacking 3-methyladenine glycosylase activity, is 10-100-fold more sensitive than the parental strain. The response of the yeast strains indicates that there is considerable overlap among the DNA repair pathways for removing cytotoxic HN2-induced DNA damage.

P35 ROLE OF DNA DAMAGE AND REPAIR IN THE SENSITIVITY OF NON-SMALL CELL LUNG CANCER CELL LINES TO MITOMYCIN C ,
N.Robertson* and I.J.Stratford., MRC Experimental Oncology Group , School of Pharmacy and Pharmaceutical Sciences , University of Manchester , Manchester M13 OPL.

Mitomycin C (MMC) is used clinically to treat a wide variety of solid tumours, and is the single most active agent for the treatment of non-small cell lung cancer (NSCLC). Activation of MMC leads to the formation of free radicals and active alkylating species, thereby producing DNA strand breaks, monoadducts and crosslinks. Cellular responses to such damage include cell cycle arrest through activation of 'checkpoint' mechanisms and the triggering of apoptosis. Cell cycle arrest provides the cell with opportunities for DNA repair, affecting the long term survival of the cell.

Previous studies in this laboratory have shown a wide variation in the response of NSCLC cell lines to MMC (Robertson *et* al, Biochem. Pharmacol., **44,** 409-412, 1992), and this cannot simply be explained by the levels of reductase enzymes present in the cells.For this study, three NSCLC cell lines have been chosen with similar levels of DT diaphorase (DTD) activity (Robertson *et al*, Eur. J. Cancer., **30A** (7) , 1013-1019 , 1994) and cytochrome P450 reductase (P450R) activity, but with a 73-fold range in their response to MMC. The cell lines , A549 , H647 and H460 , exhibit high levels of DTD (4980 , 4970 and 5020 nmol cyt. C reduced $min^{-1}$ $mg^{-1}$ protein , respectively ) and relatively low levels of P450R (22 , 9.1 and 17.6 nmol cyt. C reduced $min^{-1}$ $mg^{-1}$ protein , respectively). MMC sensitivity, as measured by the MTT assay (3 hour drug exposure), is shown by IC50 values of 0.18 μM for H460, 3.55 μM for A549, and 13.1 μM for H647.

The aims of this study are to determine whether differences in MMC sensitivity among the three cell lines are related to different cell cycle effects, or to differences in the DNA damage, namely crosslinks, formed after drug exposure,. FACS analysis of propidium iodide stained cells shows cell cycle delays in S and G2 phases of varying duration in these cell lines post-MMC treatment. Using alkaline elution, we are able to correlate drug dose with the total amount of crosslinking measured for each cell line after MMC exposure. However, we find no difference between the three cell lines in the relative amounts of crosslinks formed after MMC exposure at the same dose , despite the apparent large differences in cellular sensitivity. These results suggest that the differences in MMC sensitivity evident in these cell lines may be due to the differing abilities of the cell lines to repair DNA damage , as opposed to the total amount of damage formed. Experiments are currently underway to investigate this further.

P36 $O^6$-METHYLGUANINE-DNA METHYLTRANSFERASE (MGMT) IN TUMOUR BIOPSIES AND PERIPHERAL BLOOD LYMPHOCYTES AS AN INDICATOR OF CLINICAL RESPONSE OF MALIGNANT MELANOMA TO TEMOZOLOMIDE.
M.R.Middleton*[1], S.R.Wedge[2], G.Rustin[3], M.J.Lind[4], C.Morris[1], D.R.Newell[4], N.M.Bleehen[5], N.Thatcher[1], J.M.Lunn[4], A.H.Calvert[4] and G.P.Margison[1]. [1]Christie Hospital, Manchester, U.K., [2]Charing Cross Hospital, London, U.K., [3]Mount Vernon Hospital, Northwood, Middlesex, U.K., [4]University of Newcastle upon Tyne, U.K. and [5]Addenbrookes Hospital, Cambridge, U.K., on behalf of the Cancer Research Campaign Phase II Clinical Trials Committee.

Resistance of mammalian cells to methylating and chloroethylating agents *in vitro* and *in vivo* has been linked to levels of the DNA repair protein MGMT. In a clinical trial of temozolomide in advanced malignant melanoma, we have studied the relationship between pretreatment MGMT levels in biopsies of cutaneous tumours and involved lymph nodes, and clinical response to the drug. We have also examined tumour distribution of MGMT by immunohistochemistry. In addition, we have measured MGMT levels in peripheral blood lymphocytes (PBL) before and during temozolomide treatment.
Among 24 patients, there were 4 complete responses (CR), 3 partial responses (PR), 1 no change (NC) and 16 with progressive disease (PD), for an overall response rate of 29%. The tumour MGMT levels (fmol/mg protein) were: CR, 206±194; PR, 620±820; NC, 164; PD, 181±225. Temozolomide treatment resulted in lowering of MGMT levels in PBL, but there was no correlation between pretreatment levels in PBL and biopsies. Thus, measurements of pretreatment levels of MGMT in melanoma did not predict response to temozolomide. Further, PBL cannot be used as a surrogate for tumour biopsies.

P37 AN ALTERNATIVE METHOD FOR THE DETECTION OF GENOMIC INSTABILITY IN COLORECTAL CARCINOMAS,

M. G. Coleman*[1],A. C. Gough[1], D. J. Bunyan[2] and J. N. Primrose[1]. [1]University Department of Surgery (816) F level, Centre block, Southampton General Hospital, Southampton, Hants, SO16 6YD.[2]The Wessex Regional Genetics Laboratory, Salisbury District hospital, Salisbury, SP2 8BJ.

Microsatellite instability (MIN), implying a replication error phenotype (RER+), characterises a proportion of colorectal cancers. Generally, the polymerase chain reaction (PCR) using several microsatellite markers may be required and debate remains as to how many positive results are needed to infer RER positivity. Minisatellite variant repeat units (MVRs) are long DNA sequences which show extreme levels of allelic variability, not only in repeat copy number but also the interspersion pattern. MVR mapping by PCR (MVR-PCR) assays the interspersion pattern of variant repeat units along minisatellite alleles. We have designed novel sets of primers specific to two MVR-PCR loci (MS31A and MS32) and simplified their detection to non-radioactive PCR. Analysis of thirty-two colorectal tumour-normal mucosa pairs using MVR-PCR were compared to those using 10 microsatellite markers. Four patients (12.5%) showed MIN with 3 or more of the microsatellite markers suggesting a RER+ phenotype. All three were positive by MVR-PCR. Three cases showed an RER+ phenotype by MVR-PCR which was not observed with any of the microsatellite markers and in two of these we have identified mutations in a DNA mismatch repair gene (hMLH1). These results suggest that MVR-PCR may be an alternative, simpler method of detecting RER+ phenotype.

P38 DELETION OF THE TRANSFORMING GROWTH FACTOR β RECEPTOR TYPE II AND REPLICATION ERROR POSITIVE COLORECTAL CANCER

D Braham*, M Coleman, H Chave, D Eccles, AC Gough, K Palmer and JN Primrose University Surgical Unit and Department of Genetics, Southampton General Hospital, Southampton, SO16 6YD.

**Introduction:-** Loss of a functional transforming growth factor beta receptor type II (TGFβ RII) renders colorectal cells insensitive to the negative regulatory effects of TGFβ. It has been hypothesised that this allows escape from normal growth control mechanisms and hence contributes to malignant transformation in the colon. By virtue of two regions of repeated DNA sequences within its gene it is suggested that the TGFβ RII may be lost in tumours with DNA mismatch repair deficiency (Replication error (RER+) tumours) hence contributing to the malignant phenotype in these tumours. To investigate this possibility we have studied the expression of the TGFβ RII gene in 24 samples of colorectal cancer and normal mucosa by semi-quantitative reverse transcriptase polymerase chain reaction and polyacrylamide gel electrophosesis. The RER status of the tumours was also determined using 9 commonly used microsatellite markers. Mutational analysis of TGFβ RII was performed by restriction digest (*Bsl-1*).

**Results:-** Two of 2 (100%) RER+ tumours and 6 of 22 (27%) RER- tumours exhibited loss of TGFβ RII relative to normal mucosa. No mutations were identified in the amplified segment of the TGFβ RII gene.

**Conclusion:-** The loss of TGFβ RII is common in colorectal cancer and although it may be associated with RER+ tumours it is not confined to them.

P39 **DNA-DEPENDENT PROTEIN KINASE AND POLY(ADP-RIBOSE) POLYMERASE INHIBITORS: EFFECTS ON IONISING RADIATION INDUCED CYTOTOXICITY AND DNA DOUBLE AND SINGLE STRAND BREAK REPAIR**

Sallyanne Boulton, Suzanne Kyle & Barbara W. Durkacz*. Cancer Research Unit, University of Newcastle upon Tyne, NE2 4HH.

Poly(ADP-ribose) polymerase (PADPRP) and DNA-dependent protein kinase (DNA-PK) constitute an unusual family of enzymes that are activated by DNA strand breaks and mediate DNA repair. We investigated the effects of inhibitors of these two enzymes on the cellular responses to DNA damage. NU1025 (8-hydroxy-2-methylquinazolin-4(*3H*)-one) is a PADPRP inhibitor which potentiates cytotoxicity and inhibits DNA single strand break (SSB) repair induced by temozolomide (Boulton *et al* (1995) *Br. J. Cancer* 72: 849). Wortmannin is an inhibitor of DNA-PK, which belongs to the phosphatidylinositol 3-kinase family of enzymes. It enhances the cytotoxicity of ionising radiation (IR) and inhibits the repair of IR-induced DNA double strand breaks (DSBs) (Boulton *et al* (1996) *Carcinogenesis* **17**: 2285).

Possible interactions between the two enzymes were investigated. Both are recruited to DNA ends, and could modify each other, and hence affect DNA repair. The interactive effects of NU1025 and wortmannin on the responses to IR- and temozolomide-induced DNA damage in Chinese hamster ovary-K1 cells was studied. Both drugs were non-toxic and did not cause DNA strand breakage. NU1025 (100μM) potentiated the cytotoxicity of IR ($DEF_{10}^{*} \leq 1.5$), and retarded both SSB and DSB repair in a dose-dependent manner. Wortmannin (20μM) also potentiated the cytotoxicity of IR ($DEF_{10} \sim 5$) and inhibited DSB repair, but had no effect on SSB repair. When NU1025 and wortmannin were combined, their potentiating effects on IR cytotoxicity were additive. Both wortmannin or NU1025 alone potentiated the cytotoxicity of temozolomide, and again the potentiating effects of the combined drugs were additive. The combinatorial effects of the drugs on DSB and SSB levels in IR-treated cells are currently under investigation. Temozolomide DSBs were not detectable within the sensitivity of the neutral elution assay, but the ability of wortmannin to potentiate temozolomide cytotoxicity implicates DSB formation as a component of its cytotoxic mechanism. Although NU1025 modulated DSB repair, indicating that PADPRP was competing with DNA-PK for the same break sites on the DNA, the additive effects of the two inhibitors on potentiation of cytotoxicity suggested that the two enzymes functioned by independent pathways to mediate DNA repair.

* $DEF_{10}$: Dose enhancement factor at 10% survival.

P40 RADIATION INDUCED CELL-CYCLE DELAYS IN ASYNCHRONOUS POPULATIONS OF NORMAL FIBROBLASTS.

C.J.Orton*[1], J.Berry[2], [1]Dept. of Experimental Radiation Oncology, [2]Dept. of Physics and Instrumentation, Christie CRC Research Centre, Manchester M20 4BX.

Introduction: Radiation damage delays the progression of cells through the cell cycle. Lethally damaged and surviving cells cease to enter mitosis, whilst cells in mitosis continue their progression. After a period of time, that depends on both the cell type and the radiation dose, cells begin to reenter mitosis. This mitotic delay appears to be largely due to a block in cell-cycle progression in $G_2$ phase, whilst cells in G1 and S phase are also delayed to a lesser extent. Most studies have examined irradiation induced cell-cycle delays in synchronous populations. It is possible to study asynchronous populations with continuous bromodeoxyuridine labelling and Hoechst 33358-Ethidium Bromide bivariate flow cytometry, which separates the first and subsequent cell cycles, due to the quenching of Hoechst fluorescence by bromodeoxyuridine. Materials: Twelve fibroblast cell strains were studied , comprising two radiosensitive and one radioresistant human strains and nine strains established from vaginal biopsies from pre-therapy patients with carcinomas of the cervix. Methods:Cells were grown to confluence, irradiated at doses of 0, 2 and 4 Gy, and then plated out at low cell density for 72 hrs. Cell-cycle analysis was then performed and compared with the control population at 0 hrs. Results:There is emptying of the original $G_2$ compartment which is inhibited by irradiation; in the most radioresistant cell strains maximum inhibition occurs at 2 Gy whereas in the radiosensitive strains inhibition doess not occur at 4 Gy. The proportion of cells which cycle (i.e. those that are labelled by bromodeoxyuridine) following irradiation correlates with the cell surviving fractions at 2 Gy, $SF_2$, (range 0.027-0.32), r=-0.86, p=0.026, at 2 Gy, and r=-0.83, p=0.04, at 4 Gy, and the level of residual DNA damage at 24 hrs following a dose of 180 Gy as measured by gel electrophoresis, r=0.87, p=0.02 after 2 Gy, and r=0.89, p=0.01 after 4 Gy. In the most radioresistant cell strains the accumulation of cells in $G_1$ in the first cycle following irradiation, is greater than that in the more sensitive cell strains and at 4 Gy this relationship reaches significance when correlated with $SF_2$, r=0.81, p=0.05.The percentage of cells entering the $G_2$ compartment in the first cycle is the same irrespective of whether the cells are irradiated or not. $G_2$/M delay measured by the ratio of the percentage of cells in $G_2$ in the first cycle, to the percentage of cells in $G_1$ in the second cycle, correlates with $SF_2$, r=-0.89, p=0.03 at 2 and 4 Gy. Conclusions: Irradiation induced cell-cycle delays correlate with radiosensitivity. Other differences in the distribution of cells throughout the cell-cycle were observed in the various cell strains and probably indicate differences at the molecular level.

P41 HEPATIC DNA DAMAGE, DETERMINED BY $^{32}P$-POSTLABELLING, OF GEOMETRIC ISOMERS OF TAMOXIFEN & ANALOGUES. K. Brown*, J.E. Brown, E.A. Martin[1], I.N.H. White[1]. Pharmaceutical Chemistry, University of Bradford, Bradford BD7 1DP and [1]MRC Toxicology Unit, University of Leicester, Leicester LE1 9HN.

The antioestrogen tamoxifen is currently undergoing evaluation as a chemoprotective agent in healthy women at an increased risk of developing breast cancer. Recently tamoxifen has been shown to induce DNA adduct formation and liver tumours in rats, whilst in patients it is associated with an increased incidence of endometrial carcinoma. To determine the risk of tamoxifen treatment it is vital to know the mechanisms involved in tamoxifen activation leading to tumour formation. Tamoxifen (*trans* isomer) exhibits complex pharmacological properties, with both oestrogen antagonistic and agonistic actions depending on the species and target organ, whereas the *cis* isomer is a pure oestrogen agonist.

In this study, geometric isomers of tamoxifen and related compounds were assessed for their ability to cause DNA damage using $^{32}P$-postlabelling (Martin, E.A. et al. Carcinogenesis, 16, 1651-1654, 1995), the aim being to determine the contribution of each individual isomer to DNA adduct formation. The compounds tested were tamoxifen itself, α-hydroxytamoxifen, a metabolite postulated to be an intermediate in tamoxifen activation (Osborne, M.R. et al. Cancer Res., 56, 66-71, 1996) and C-desmethylene tamoxifen, an analogue in which the ethyl group has been replaced by a methyl. Female Fischer rats received 40mg/kg i.p. daily for 4 days, the liver DNA was then extracted and subjected to $^{32}P$-postlabelling.

Table 1. $^{32}P$-Postlabelled DNA adducts, in rat liver.

| Compound | No of determinations | Total adducts/$10^8$ Nucleotides Mean ± SD |
|---|---|---|
| *Trans tamoxifen* | 6 | 103.4 ± 19.3 |
| *Cis Tamoxifen* | 6 | 18.0 ± 3.5 |
| *Trans C-desmethylene tamoxifen* | 5 | 95.5 ± 22.8 |
| *Cis C-desmethylene tamoxifen* | 5 | 1.9 ± 0.5 |
| *Trans α-hydroxytamoxifen* | 6 | 4947.1± 1675.6 |
| *Cis α-hydroxytamoxifen* | 6 | 52.7 ± 14.5 |

These results show that treatment with the *trans* isomer resulted in a 5 to 90 fold higher level of adduct formation than with the corresponding *cis* isomer. Differences in adduct formation may be attributed to both differences in the metabolism and detoxification of the isomers.

P42 **PROGENITOR GROWTH FACTORS INCREASE THE DNA DAMAGING EFFECTS OF ETOPOSIDE IN CULTURED PERIPHERAL BLOOD MONONUCLEAR CELLS.** Wai M. Liu*, Clare L. Bamford, Simon P. Joel, ICRF Department of. Medical Oncology, St. Bartholomew's Hospital, London.

Prior to work on the influence of dose and schedule of the topoisomerase II inhibiting drug etoposide (VP-16) in bone marrow cells, the DNA damaging effects of three haemopoietic growth factors necessary for short term bone-marrow cultures (stem cell factor (SCF), interleukin 3 (IL-3) and granulocyte/macrophage colony stimulating factor (GM-CSF)), in combination with VP-16, were investigated. Sister chromatid exchange (SCE) frequencies in the peripheral blood mononuclear cells (MNC) of clinically normal volunteers were used as an indicator of the level of damage to DNA. MNC's were separated on Ficoll, mitogenically stimulated with phytohaemagglutinin-M, and cultured with one of the three cytokines (0-100 ng/ml), or with etoposide (0-2 μM) for 2 days. The effect of each growth factor and VP-16 in combination were assessed at concentrations of 40 ng/ml for each cytokine and 0.4 μM VP-16. The number of SCE per metaphase was scored by Hoechst/Giemsa staining. SCF alone did not cause significant changes in SCE numbers (5.0 ± 0.6 at 100 ng/ml vs 5.0 ± 0.6 in control cultures). However, GM-CSF and IL-3 induced small, but significant, dose dependent increases in the frequency of SCE's at concentrations of 50 and 100 ng/ml (100 ng/ml GM-CSF 6.4 ± 0.8, $p<0.0001$ vs control, 100 ng/ml IL-3 6.9 ± 0.8, $p<0.0001$ vs control). With VP-16 alone there was a dose dependent increase in SCE frequency which reached a plateau at around 0.4 μM (20.6 ± 1.9 at 0.4 μM VP16 vs 5.0 ± 0.4 in controls). When growth factor and etoposide were used in combination there was an approximate 50% increase in SCE number, with any of the growth factors used, compared to VP-16 alone (20.6 ± 1.7 vs 29.8 ± 2.3 with SCF, 30.2 ± 2.0 with GM-CSF and 30.2 ± 1.8 with IL-3, $p<0.0001$ in each case). These data suggest a sensitivity of MNC's, in this model system, to SCE induction by growth factors either used alone or in combination with VP-16. Investigators should be aware that the necessary use of growth factors in bone marrow cultures may influence the activity of DNA damaging agents.

P43 RADIATION RESPONSE IN TONGUE CARCINOMA CELL LINES, N.A.Andrews*A.S.Jones* and A.R.Kinseila[1], Dept of Surgery[1], Dept of Otorhinolaryngology University of Liverpool PO Box 147 Liverpool L69 3BX.

In this study we examined the effect of γ irradiation induced cell cycle delay, and radiosensitivity in three tongue carcinoma cell lines with respect to p53 expression. The cell lines used were HN5, B2A4 (subclone of HN5), and GBi2A (invasive population from HN5). Immunohistochemical analysis using the p53 antibody Ab6 detected overexpression of the p53 protein in both the HN5 and the GBi2A cell lines, but no expression of the protein was detected in the B2A4 cell line. Twenty four hour cell cycle data was obtained after 4 grays of radiation using FACS analysis. The parental cell line HN5 exhibited maximal G2 arrest at 14hrs post irradiation with normal cell cycle response returning after 22hrs. The morphological features of apoptosis were assessed by Acridine Orange using cells floating in the medium after 24 hours. No evidence of apoptosis was seen in this cell line even after 48hours. The invasive derivative GBi2A showed evidence of G2 arrest at 14 hours post irradiation with G2 arrest persisting past the twenty four hour period. No apoptosis was noted after 24 hours. The subclone B2A4 exhibited G2 arrest only 4 hours post irradiation with maximal G2 arrest at 8 hours post irradiation. Normal cell cycling was regained after 12 hours. There was evidence of apoptosis after 24 hours. The survival fraction data between the cell lines after 4grays varied from 0.38 in the HN5 cell line to 0.54 in the invasive cell line GBi2A, with the B2A4 SF being 0.29.

Taken together these data indicate that overexpression of p53 is indicative of an abnormality, in p53 function, and is not only involved in G2 arrest but also the length of the G2 arrest. It is also evident that overexpression results in the concomitant impairment of the apoptotic function in the parental cell line HN5 and the invasive subclone GBi2A. It would appear that the B2A4 subclone has normal p53 by virtue of its lack of overexpression and the ability to apoptose.

P44 Over expressed p53 in Germ Cell cancers as a possible factor in their high chemosensitivety. Nouri AME, Dabare AANPM, Oliver RTD.Medical Oncology, London Hospital. UK.

It has long been known that seminoma is more radiosensitive than non-seminoma, though both are a considerable order of magnitude more radiotherapeutically and chemotherapeutically curable than any other adult solid cancer.

In recent year high frequency of p53 mutation has been reported in various human malignancies. Testis tumour is an exception in that the over expressed p53 is in a non-mutated form and this could be an important determinant of response to chemotherapy.

Immunocytochemical staining was set up to study of 40 germ cell cancers, 31 bladder cancers and 27 squamous head and neck cancers using monoclonal antibodies (Mab) specific for p53, Bcl-2 and HSP70.

Results showed that 89% of germ cell cancer compared to 7% of bladder and 2% of squamous head and neck cancers were positive for p53 using 240 Mab while only 60% of germ cell cancers but 100% of bladder and head and neck were positive for Bcl2. The observation of higher p53 positivity for seminoma (82%,n=17) compared to non seminoma (33%, n=6) and the inverse for Bcl2-2 (53% vs. 83%) provides possible additional support for the increasing literature suggesting a role for p53 in germ cell cancer chemosensitivity given the differential chemosensitivity of these two subtype.

P45 **CLONING OF A YEAST SEQUENCE THAT CONFERS CISPLATIN RESISTANCE TO TESTIS TUMOUR CELLS.** Majid Hafezparast*, Xianghong Wang and John RW Masters. Institute of Urology and Nephrology, University College London, 3rd Floor, 67 Riding House Street, London W1P 7PN

In contrast to most metastatic cancers, testicular germ cell tumours are highly sensitive to DNA damaging agents. We have therefore used testis tumour cells to determine the genetic basis of curability of testicular germ cell tumours. However, these cells are difficult to grow *in vitro* and have low transfection frequencies, making conventional cloning strategies impractical. For this reason we have generated an EBNA-1 (Epstein-Barr Nuclear Antigen 1) expressing cell line of testis, SuSa-E46, that has a transfection efficiency of 0.15%. The EBNA-1 gene promotes episomal replication of EBV-based vectors and hence increases the stable transfection frequency of cells, in this case 75 fold higher than the parent line SuSa. We have used *S. cerevisiae* yeast genomic and human cDNA libraries, in EBV-based vectors, to transfect SuSa-E46 cells and have isolated cisplatin resistant clones. Hirt extraction of plasmids from the resistant clones resulted in isolation of several plasmids containing different-sized inserts. So far, one yeast insert has been taken through secondary and tertiary transfections. This insert confers cisplatin resistance and morphological changes to SuSa-E46 cells and is being sequenced. Plasmids rescued from a human cDNA transfectant that is resistant to cisplatin will be used to generate secondary transfectants and hence identify the cDNA insert that confers this resistance.

P46 **BAX EXPRESSION IN PRIMARY EPITHELIAL OVARIAN CARCINOMA: ASSOCIATION WITH WILD-TYPE P53.** *A. Al-Azraqi*[1]* *C. Challen*[1], *D. McKenna*[2], *B. Angus*[2], *M. George*[3], *D. Sinha*[3], *A. H. Calvert*[1] *and J. Lunec*[1]. [1]Cancer Research Unit, and [2]Department of Pathology, Medical School, University of Newcastle upon Tyne, Newcastle NE2 4HH; [3]Department of Gynaecological Oncology, Queen Elizabeth Hospital, Gateshead.

Carboplatin is one of the main treatments for epithelial ovarian carcinoma. *In vitro* studies have shown a role for wild-type p53 function in mediating the apoptotic effect of platinum-based drugs. We have previously reported a significant correlation between absence of p53 mutation and initial clinical response to carboplatin, between Bcl-2 positive staining and good response of the same cases and between Waf1 positive staining and survival in wt-p53 cases. Bax is an apoptosis inducing factor which interacts with and counteracts the effect of Bcl-2. It is a 21 kd protein and transcription of the gene is positively regulated by wild type p53. The bax coding sequence is preceded by four copies of the p53 recognition motif. This study aimed to investigate the status of Bax expression in 38 ovarian carcinoma cases prior to surgical debulking and treatment with single-agent carbolpatin. Bax polyclonal antibody (Pharmingen) was used at a dilution of 1/2000 for immunoperoxidase staining of formalin-fixed 5 μm sections with microwave antigen retrieval. Irradiated skin sections were used as positive controls. In this study, Bax positive immunostaining was significantly associated with the lack of p53 mutation (p=0.014, n=38; Fisher's test). Bax positive staining was not associated with initial response of patients to treatment (p=0.59, n=31); however, in those cases which were positive for Bax and Bcl-2, the association with good response is marginally significant (p=0.06, n=24). Our results confirm an association *in vivo* between wild-type p53 function and Bax expression. However, Bax expression alone failed to show a relationship to treatment or survival.

P47 **ROLE OF BAK AND BCL-$x_L$ IN CISPLATIN RESISTANCE IN OVARIAN CARCINOMA CELL LINES.** **P.J. Beale*, S.Y. Sharp and L.R Kelland. CRC Centre for Cancer Therapeutics, Institute of Cancer Research, Sutton, Surrey.**

Cisplatin resistance in ovarian carcinoma is known to be multifactorial. The role of the BCL-2 family of proteins in this process is not well defined. High levels of BCL-2 measured immunohistochemically is known to confer a better prognosis but other data suggest that high levels may be associated with drug resistance *in vitro*. BAK and BCL-$x_L$ are two proteins of the Bcl-2 family; BAK is a pro-apoptotic protein and BCL-$x_L$ is anti-apoptotic. Levels of BAK protein as measured by densitometry after western blotting ranged from 10 to 172 (arbitrary levels) in our panel of 17 human ovarian carcinoma cell lines. Levels of BCL-$x_L$ ranged from 0.6 to 28. There was no correlation with levels of BAK or BCL-$x_L$ and the 96 hour $IC_{50}$ to cisplatin, as measured by SRB assay ($r^2$ = 0.003 and 0.06 respectively). In sensitive and acquired cisplatin resistant lines, BAK levels were lower in the resistant line (A2780cisR and 41McisR) but equal in the CH1 pair. BCL-$x_L$ levels were lower in the resistant line (A2780cisR and 41McisR) and there was a small rise in the level in the CH1cisR line. Following a 2 hour exposure to cisplatin (5 x 2 hour $IC_{50}$) levels of BAK and BCL-$x_L$ were measured up to 24 hours after removal of drug. In the A2780 line transfected with Bcl-2 and the neo-transfected pair the pattern of change in protein levels varied. In the neo-transfected line, by 24 hours the level of BAK was only 0.41-fold of that at time zero; levels of BCL-$x_L$ rose 1.5-fold and there was no BCL-2 detected. BAX, p53 and p21 levels increased at 24 hours. In the Bcl-2 transfected line, however, BAK rose to 3.3-fold that of time zero at 12 hours and BCL-$x_L$ levels altered only slightly (1.1-fold at 24 hours). BCL-2, BAX, p53 and p21 levels increased at 24 hours. Levels in other lines have been measured after exposure to cisplatin and similar results have been seen. These data support the lack of importance of these proteins in cisplatin resistance in these cell systems.

This work is supported by the Cancer Research Campaign.

P48 **DETECTION OF AMPLIFIED AND DELETED GENES IN SENSITIVE AND CISPLATIN-RESISTANT HUMAN OVARIAN CELL LINES BY COMPARATIVE GENOMIC HYBRIDIZATION (CGH) AND FLUORESCENCE IN SITU HYBRIDIZATION (FISH).** **[1]S.Y. Sharp*, [2]L.R. Hiorns and [1]L.R. Kelland. [1]CRC Centre for Cancer Therapeutics & [2]Academic Dept. of Haematology, The Institute of Cancer Research, Sutton, Surrey.**

Acquired resistance to cisplatin has been generated *in vitro* in the 41M human ovarian carcinoma cell line, established from a previously untreated patient. Three cisplatin resistant variants were selected at approximately 2, 4 and 6-fold resistance (41McisR2, 41McisR4 and 41McisR6, respectively). Previous biochemical studies have shown that reduced drug uptake is the major determinant of acquired cisplatin resistance in the 41McisR2 (2.0 ± 0.6), 41McisR4 (3.6 ± 1.0) and 41McisR6 (4.8 ± 0.6) cells, when compared with the sensitive 41M cells (Loh *et al.*, 1992). To gain a better understanding of the genetic changes which may be involved in the reduced transport mechanism(s) in these cell lines, two complementary molecular cytogenetic techniques were used. CGH produces a genome-wide screening of DNA sequence copy number abberations, where differentially labelled sensitive and resistant cell lines are hybridized simultaneously to normal metaphase spreads. Regions of gain or loss of DNA sequences (ie. amplifications or deletions) can then be visualized. The CGH results showed that amplifications were observed in the 41McisR6 cells for regions of chromosomes 4q, 5q, 6q, 10, and 17p, and deletions for regions of 3q, 5p, 9p and 12p. A number of candidate genes are located in these positions. In order to confirm the CGH findings and identify the genes involved, FISH with specific probes was performed. Among the genes located on chromosome 10 are genes for mitochondrial ATPase and potassium channels. Probing with a whole chromosome 10 paint showed the parental 41M cell line had only one complete copy of chromosome 10 and a portion translocated to another chromosome. As cisplatin resistance increases in the 2-, 4- and 6- fold resistant cell lines, increasing numbers of fragments derived from chromosome 10 were observed. The Tel-1 gene (a transcription factor commonly expressed in acute lymphoid leukaemia) was found not to be involved in the deleted region of chromosome 12 in the 41McisR6 variant. However, it was a useful marker to show progressive rearrangement and deletion of the short arm of chromosome 12 with increasing cisplatin resistance. We are currently probing with a whole chromosome 5 paint (which has a gene for Na/K/Cl transporter) and p53 (located on chromosome 17p).

Ref: Loh *et al.*, Br J Cancer, 66, 1109-1115, 1992.
*This work was supported by Cancer Research Campaign.*

P49

### CHARACTERISATION OF P53 STATUS IN HUMAN OVARIAN CANCER CELL LINES SENSITIVE TO CISPLATIN.

KE Pestell*, CJ Medlow, J Titley, LR Kelland, MI Walton. CRC Centre for Cancer Therapeutics, ICR, 15 Cotswold Road, Belmont, Sutton, Surrey, SM2 5NG, UK.

We have studied the p53 status in a panel of human ovarian cancer cell lines relatively sensitive to cisplatin to determine whether this gene may be important in governing cisplatin sensitivity. The cell lines investigated were LK1, LK2, PA1, OVIP, CH1 and the known wild type p53 expressing cell line A2780. Functional p53 status was determined by measuring p53 induction 4 hours following 5 Gy using immunoblotting techniques. $G_1$/S checkpoint integrity was determined by measuring bromodeoxyuridine incorporation 16 hours following 5 Gy (4 hour pulse) and subsequent FACS analysis. Single-strand conformation polymorphism (SSCP) and direct PCR sequencing of exons 5, 6, 7 and 8 of p53 were carried out using standard techniques. Chemosensitivity to cisplatin was determined by 96 hour SRB growth delay assay. Constitutive p53 protein levels were barely detectable in A2780, CH1, LK1, LK2 and PA1, however following irradiation levels were induced greater than 4-fold, suggesting wild type p53 function. In OVIP constitutive p53 protein was readily detectable and levels were only induced 2-fold following irradiation. There was marked $G_1$/S arrest in A2780 (75.0 ± 13.9, % mean ± SD, n = 4) following irradiation but a range of partial arrests in LK2, OVIP, PA1, CH1 and LK1, with 60.5 ± 9.8, 55.9 ± 1.5, 55.5 ± 12.0, 47.4 ± 5.1 and 28.8 ± 15.8 % arrests respectively, suggesting wild type p53 protein induction cannot be directly correlated with $G_1$/S arrest capacity. No mutations were found in A2780, CH1, LK1, LK2 and PA1 but a heterozygous point mutation in OVIP was found in exon 5: an A to G conversion at codon 126, resulting in a switch from tyrosine to cysteine. The wild type p53 expressing lines A2780, LK1, CH1, PA1 and LK2 were relatively sensitive to cisplatin with $IC_{50}$ values of 0.33 ± 0.11, 0.16 ± 0.07, 0.11 ± 0.02, 0.10 ± 0.05, 0.09 ± 0.02 μM (mean ± SD, n = 3) compared to 4.2 ± 1.5 for the heterozygous p53 mutant OVIP. This work suggests that wild type p53 expression may have a role in governing cisplatin sensitivity in these ovarian cancer lines.

P5]

### Identification of a p53-derived peptide which confers increased cisplatin sensitivity to drug resistant cells.

M. Cairney, W. Gallagher and R. Brown. CRC Department Medical Oncology, CRC Beatson Laboratories, Switchback Road, Glasgow, G61 1BD

**Loss of p53 function is associated with the acquisition of cisplatin resistance in the human ovarian adenocarcinoma A2780 cell line. Selection for cisplatin resistance of A2780 cells was used to isolate genetic suppressor elements (GSEs) from a retroviral library expressing random fragments of human or murine TP53 cDNA. Six GSEs were identified, encoding either dominant negative mutant peptides or antisense RNA molecules which corresponded to various regions within the TP53 gene (Gallagher, et al (1996) Oncogene, *in press*). Expression of antisense GSEs led to decreased intracellular levels of p53 protein. A 17 amino acid synthetic peptide (PEP-1F), representing the predicted amino acid sequence of a sense GSE, inhibited the sequence specific DNA binding activity of baculovirus expressed p53 protein *in vitro*. We have previously shown that a dominant negative mutant of TP53 when introduced into the cisplatin resistant A2780/cp70 line gives increased sensitivity to cisplatin. We now show that introduction of the peptide PEP-1F into A2780/cp70 cells confers increased sensitivity to cisplatin. The p53-derived PEP-1F peptide or the control reverse peptide were introduced into A2780/cp70 cell by lipofection. The synthetic peptides (10μM final concentration) were transfected into A2780 cells using the cationic lipid DOTAP. Cells were treated with a range of concentrations of cisplatin 24h after application of the peptide. Cell viability was measured 3 days after cisplatin treatment using the MTT cell viability assay.**

| Cisplatin concentration | Cell viability (1) | | Significance (2) |
|---|---|---|---|
| | PEP-1F | PEP-1R | |
| zero | 1.08 | 1.11 | >0.1 |
| 20μM | 0.28 | 0.35 | 0.02 |
| 4μM | 0.5 | 0.77 | 0.004 |
| 0.16μM | 0.87 | 1.09 | 0.001 |
| zero | 1.01 | 1.09 | >0.1 |

**(1) Cell viability is measured using the MTT assay either after application of the test p53-derived PEP-1F peptide or the control reverse peptide PEP-1R. Results represent the average of eight independent assays.**
**(2) Statistical analysis was using a one-tailed t-test.**

**Thus, the p53-derived PEP-1F peptide significantly sensitises A2780/cp70 to the growth inhibition effects of cisplatin compared to the control reverse peptide.**

P51

### THE DEVELOPMENT OF AN RT-PCR BASED ASSAY FOR THE ANALYSIS OF DIHYDROPYRIMIDINE DEHYDROGENASE EXPRESSION IN TISSUES.

SJ Johnston*, SA Ridge, J Sludden, and HL McLeod. Department of Medicine and Therapeutics, Institute of Medical Sciences, University of Aberdeen, Foresterhill, Aberdeen, UK.

Dihydropyrimidine dehydrogenase (DPD) is the rate limiting enzyme of 5-fluorouracil (5FU) reduction. 5FU is rapidly degraded by DPD and its antitumour efficacy may be influenced by patient DPD activity. DPD activity is highly variable in populations and has been correlated linearly with DPD protein expression. However, the *ex vivo* methods currently used to assess DPD activity may not accurately reflect activity *in vivo* and so may be an unreliable indicator of patient metabolic capacity.

We have developed a highly sensitive reverse transcriptase PCR (RT-PCR) based assay for the analysis of DPD mRNA expression in all types of tissue including colon tumours. This assay will allow us to study the relationship between DPD mRNA levels and enzyme activity. A competitor template molecule has been synthesized which competes with the wild type cDNA for primer binding. The two PCR products are of different sizes; the wild type being 465bp and the competitor 400bp. By titrating known amounts of the competitor against standard amounts of the wild type cDNA the levels of DPD cDNA within a sample can be measured using densitometric analysis of ethidium bromide stained agarose gels.

DPD activity is highest in liver tissue and lower in other tissues such as lymphocytes, spleen, and gastro-intestinal tract. RNA from human liver and spleen was used to determine the reproducibility of the RT-PCR assay yielding coefficients of variation of 12.3% and 11.9% respectively. This assay also showed expression in the liver to be 1.7 fold greater than in the spleen. This was less than the 3.5 fold difference in catalytic activity seen between the tissues ( liver - 211.7 versus spleen - 61.0 pmol $min^{-1}$ $mg^{-1}$ protein ). RNA has been extracted from, and activity measured in human, rat, and mouse livers, human lymphocytes, gastro-intestinal mucosa, and colon tumours for the comparison of DPD expression and enzyme activity.

This assay provides a useful tool for the study of DPD mRNA *in vivo* and will allow assessment of the clinical utility of DPD expression as a factor in optimising 5FU based chemotherapy.

This work was supported by a University of Aberdeen Research Committee grant and a Faculty of Medicine studentship.

P52

### ALTERED EXPRESSION OF FOLYLPOLYGLUTAMATE SYNTHETASE (FPGS) GENE IN HUMAN LEUKAEMIA CELLS WITH DEFECTIVE POLYGLUTAMATION OF RALTITREXED (ZD1694),

Yuzuru Takemura*[1], Hiroyuki Kobayashi[1], Hayato Miyachi[2] and Ken-ichi Furihata[3], [1]Dept. Lab. Med., Natl. Defense Med. Coll., Tokorozawa, Saitama 359, [2]Dept. Clin. Pathol., Tokai Univ. Sch. Med., Isehara, Kanagawa 259-11 and [3]Dept. Lab. Med., Shinshu Univ. Sch. Med., Matsumoto, Nagano 390, Japan.

A human acute lymphoblastic leukemia cell line, MOLT-3, has been cultured in RPMI-1640 medium supplemented with 10% fetal bovine serum in the continuous presence of raltitrexed (ZD1694) for the establishment and subsequent characterization of the resistance to this novel thymidylate synthase (TS) inhibitor. A 1600-fold resistant subline has been selected after 6-months exposure to the drug up to 1 mM and designated as MOLT-3/ZD1694•C. The raltitrexed-resistant subline showed a cross-resistance to methotrexate (120-fold) only under short-term drug-exposure conditions and a significant collateral sensitivity to trimetrexate (0.033-fold) which is a non-polyglutamatable dihydrofolate reductase inhibitor. The cells demonstrated extremely low accumulation and poor retention of [$^3$H] ZD1694, with no change of initial drug uptake and a minimal increase of TS activity. HPLC analysis displayed a virtual absence of ZD1694-polyglutamates in the resistant cells (Y. Takemura, et al., *Int. J. Cancer* 66 : 29-36, 1996). However, folylpolyglutamate synthetase (FPGS) mRNA was only moderately decreased in spite of completely defective polyglutamation of the drug. The growth of the resistant cells was only minimally affected either in drug-free or in drug-containing culture medium, suggesting that physiological folates essential for cell survival and proliferation could be effectively polyglutamated. Molecular search of FPGS gene by direct-sequencing of FPGS mRNA extracted from the sensitive and raltitrexed-resistant MOLT-3 cells demonstrated an alternative splicing skipping exon 12 of the gene sequence reported. Furthermore, a deletion of guanine at nucleotide 1252 in exon 13 was identified in a single allele of the FPGS gene in the resistant cells, resulting in a frame shift of amino acid codons and an appearance of premature stop codon (TGA, nucleotides 1274-1276 in exon 13). This genetic change may result in the production of altered FPGS with decrease affinity only for the drug or class of drugs used for the selection of the resistance, but not for physiological folates. These results also indicate variable expressions of the different splice forms of FPGS mRNA in different cells.

### P53 MOLECULAR CHARACTERIZATION OF HUMAN ACUTE LEUKAEMIA CELLS RESISTANT TO ZD9331, A NON-POLYGLUTAMATABLE THYMIDYLATE SYNTHASE (TS) INHIBITOR.

Hiroyuki Kobayashi*[1], Yuzuru Takemura[1], Hayato Miyachi[2] and Kenichi Furihata[3], [1]Dept. Lab. Med., Natl. Defense Med. Coll., Tokorozawa, Saitama 359, [2]Dept. Clin. Pathol., Tokai Univ. Sch. Med., Ischara, Kanagawa 259-11 and [3]Dept. Lab. Med., Shinshu Univ. Sch. Med., Matsumoto, Nagano 390, Japan.

ZD9331 is a non-polyglutamatable, potent quinazoline antifolate inhibitor of TS, which exploits the reduced folate carrier (RFC) for cell entry. Since resistance to chemotherapeutic agents remains one of the major obstacles in the treatment of malignancies, the mechanism of resistance to a newly developed anticancer drug should be thoroughly investigated. There are three major mechanisms of resistance to the selective TS inhibitors; (1) overexpression of the target enzyme, TS, (2) impaired RFC-mediated membrane drug transport, and (3) diminished polyglutamation. In an effort to clarify the mechanism of the resistance to this novel TS inhibitor, human acute lymphoblastic leukaemia cell line, MOLT-3, has been cultured in RPMI-1640 medium supplemented with 10% fetal bovine serum in the continuous presence of ZD9331 for the establishment of the drug-resistant subline. A 300-fold resistant subline has been selected after 6-months exposure to the drug up to 7 $\mu$M and designated as MOLT-3/ZD9331. The ZD9331-resistant subline showed a cross-resistance to CB3717 (5-fold), ZD1694 (63-fold) and methotrexate (MTX) (120-fold), but retained sensitivity to trimetrexate (0.88-fold), a dihydrofolate reductase inhibitor that does not use RFC for cell entry. The MOLT-3/ZD9331 cells demonstrated impaired initial uptake and low accumulation of [$^3$H] MTX in concord with the decreased expression of *RFC1*, suggesting the down-regulation of RFC made the cells cross-resistant to antifolates using RFC-mediated transport. In addition, Northern analysis revealed the elevation in *TS* mRNA level in MOLT-3/ZD9331 cells compared with the parent MOLT-3 cells. Although no major rearrangements of the *TS* gene were noted by Southern analysis, there was a significant amplification of the *TS* gene in ZD9331-resistant cells. These results demonstrate that continuous exposure of human leukaemia cells to ZD9331 leads not only to the decreased expression of *RFC1* and subsequent impaired RFC function but also to *TS* gene amplification and overexpression which is responsible for the cross-resistance to other TS inhibitors. Since ZD9331 is not a substrate for folylpolyglutamate synthetase, the expression of this enzyme did not alter as expected. These observations suggest that the development of a particular resistance phenotype, such as amplification of the target enzyme and impaired drug transport, is likely regulated at genetic and transcriptional level to circumvent the cytotoxic effect of the drug.

### P54 IMPAIRED POLYGLUTAMATION AS A MECHANISM OF TOMUDEX RESISTANCE IN A CCRF-CEM CELL LINE

M.J.Barnes[1], E.J.Estlin[2], G.A.Taylor[1], J.A.Calvete[1], D.R.Newell[1], J.Lunec[1], A.G.Hall[2], A.D.J.Pearson[2], W.Aherne[3], A.Hardcastle[3]. [1]Cancer Research Unit and [2]Department of Child Health, University of Newcastle.UK. [3]The Institute of Cancer Research, Sutton, Surrey.UK.

A CCRF-CEM cell line that is insensitive to Tomudex, a classical antifolate thymidylate synthase (TS) inhibitor has been identified. After a 96 hr continuous exposure to Tomudex the resistant cell line (CCRF-CEM:R$^{Tomudex}$) was > 400 fold less sensitive ($IC_{50}$ 560ng/ml) when compared to the CCRF-CEM cell line ($IC_{50}$ 1.7ng/ml). The Tomudex resistant cell line was cloned and a subline (CCRF-CEM:RC2$^{Tomudex}$) was isolated. CCRF-CEM:RC2$^{Tomudex}$ cells were >1000 fold resistant to Tomudex ($IC_{50}$ 1777ng/ml for a 96 hr continuous exposure) but were fully sensitive to the non-classical TS inhibitor, AG337 ($IC_{50}$ 360ng/ml for CCRF-CEM and 281ng/ml for CCRF-CEM:RC2$^{Tomudex}$ 96 hr continuous exposure). There were no differences in isolated TS activity or $^3$H-MTX uptake between the cloned resistant and sensitive cells. However, after a 24 hr exposure to 1μM $^3$H-MTX, CCRF-CEM cells accumulated significant levels of MTX polyglutamate metabolites (2152 pmoles/$10^9$ cells) upto the pentaglutamate form. In direct contrast, CCRF-CEM:RC2$^{Tomudex}$ cells contained only parent drug (697 pmoles/$10^9$ cells) and produced no detectable polyglutamate metabolites. On exposure to 10μM $^3$H-MTX for 24 hrs, the cloned resistant cells did produce MTX diglutamate, but again lacked longer chain polyglutamates. Following a 24 hr exposure to 0.1μM Tomudex, CCRF-CEM:RC2$^{Tomudex}$ cells accumulated >50 fold less total intracellular Tomudex (6.53 pmoles/mg protein) than CCRF-CEM cells (345 pmoles/mg protein). Consistent with these findings was the FPGS activity in cell extracts with MTX as the substrate. The CCRF-CEM:RC2$^{Tomudex}$ cell line was shown to have <11% the activity of CCRF-CEM cells, but Northern blot analysis revealed similar FPGS mRNA levels in both cell lines. These data suggest either FPGS gene mutation or defective translation as the basis of Tomudex resistance due to impaired polyglutamation.

### P55

**CELLULAR RESISTANCE TO MITOMYCIN C IS ASSOCIATED WITH OVEREXPRESSION OF MDR-3 IN A UROTHELIAL CANCER CELL LINE (MGHU-1).**

**M C Hayes*, A J Cooper, K-J Palmer, A C Gough. University Dept. of Surgery, Southampton General Hospital, SO16 6YD.**

Mitomycin C (MMC), an antitumour antibiotic, is used in the intravesical context in the treatment of superficial bladder cancer. It remains uncertain whether this agent falls within the classical multidrug resistance (MDR) family, mediated primarily by the expression of P-glycoproteins A and B, and encoded by genes MDR-1 and MDR-3 respectively. We have induced resistance to MMC in a urothelial cancer cell line (MGHU-1) and compared MDR-1 and 3 expression and MMC cytotoxicity against drug sensitive controls.

MMC resistance was induced by exposure of wildtype MGHU-1 cells to increasing concentrations (from 20 to 400 nM) of MMC in DMEM/10% FCS/glutamine over six months in culture. Cytotoxicity of MMC and epirubicin was then compared for MGHU-1, MGHU-MMC (MMC resistant) and MGHU-1R (established MDR, adriamycin-induced) by MTT biomass assay after one hour exposure in culture to varying concentrations (0.6-80 μg/ml) of MMC or epirubicin. Expression of MDR-1 and 3 was investigated for each line by RT-PCR using cDNA specific primers after titration, and compared to DNA and negative controls.

MDR-1 and 3 were both significantly overexpressed in MGHU-1R and were associated with a dramatic increase in the IC50 ($p<0.001$ paired t test) for MMC and epirubicin. In MGHU-MMC, overexpression of MDR-3 was more marked than for MDR-1 but was associated with a near-identical cytotoxiciy profile for both agents. Trace amounts of MDR-3, but not MDR-1, were identified in MGHU-1 wildtype. We conclude that urothelial cancer cell resistance to mitomycin C is associated primarily with the overexpression of MDR-3 in the MGHU-1 line and therefore suggest that mitomycin C must fall within the MDR category.

### P56 EMERGENCE OF P-GLYCOPROTEIN EXPRESSION IN HUMAN SARCOMA CELLS AFTER 72 HOUR SELECTION IN DOXORUBICIN,

H.C.L. Traunecker*[1], M.C.G. Stevens[2], D.J. Kerr[1], and D.R. Ferry[1], 1 CRC Institute for Cancer Studies, University of Birmingham, Birmingham B15 2TJ, 2 Dept. of Paediatric Oncology, The Birmingham Children's Hospital, Birmingham B16 8ET.

*In vitro* investigation of drug resistance mechanisms relies heavily on the use of resistant clones derived from their parental tumour cell line by continuous multi-step selection with a cytotoxic agent.

We investigated the emergence of doxorubicin resistant clones after a single-step, 72 hour exposure of the human uterine sarcoma cell line MES-SA to 450 or 500 nM doxorubicin after allowing the cells to grow for 15 generations by using the Luria-Delbrück fluctuation analysis. Growing cell populations, derived from a small number of cells, in parallel cultures allowed us to describe the emergence of resistant clones, calculate mutation rates and investigate P-glycoprotein expression in the resistant clones.

Three fluctuation analysis experiments were performed by exposing non-resistant, non-P-glycoprotein expressing MES-SA cells to 450 nM (n=2) or 500 nM (n=1) doxorubicin for 72 hours. The calculated mutation rates were $3.5 \times 10^{-8}$, $1.0 \times 10^{-7}$ and $3.0 \times 10^{-6}$ clones/generation (Catcheside, The Genetics of Micro-organisms, London, 1951, p 158). In the two experiments with low mutation rates 17/19 clones were analyzed. Only 2 clones were ≥ 2-fold resistant to doxorubicin compared to MES-SA cells and neither expressed P-glycoprotein.

The experiment with a high mutation rate yielded 359 clones of which 17 were analyzed. 8 clones were ≥ 2-fold resistant to doxorubicin compared to MES-SA cells. All 8 clones expressed P-glycoprotein as judged by Western blotting and had detectable [$^3$H]-vinblastine binding of ≥ 1 pmol/mg protein (Ferry et al, Biochem and Biophys Research Commun, 188:440, 1992). There was a strong correlation between [$^3$H]-vinblastine binding and resistance to doxorubicin ($n = 19$, $r = 0.8$, $p < 0.001$).

These experiments show that a short, single-step exposure of MES-SA cells to doxorubicin led to selection of clones which expressed P-glycoprotein. Other mechanisms of resistance appear to be rarely operative. The reason why non-resistant clones were selected could be due to inducible mechanisms of resistance or statistical survival of non-resistant cells. If similar biological processess occur in human tumours, even if they are initially P-glycoprotein negative, treatment may select resistant clones at a frequency not detectable by current immunohistochemical methods.

### P57 THE DEVELOPMENT OF *MDR1* AND *MRP* RIBOZYMES AS A NEW STRATEGY TO STUDY MULTIDRUG RESISTANCE IN TUMOUR CELLS.

E.L. Lewis* and I Gibson. University of East Anglia, School of Biological Sciences, Norwich NR4 7TJ.

Multidrug resistance (MDR) in tumour cells is often associated with over- expression of the human *mdr1* and *mrp* genes encoding the 170 kDa phospho- glycoprotein (P-gp) and 190 kDa multidrug resistance associated protein (MRP), respectively, and numerous studies suggest they are involved in the efflux of many structurally unrelated anticancer drugs from cultured MDR tumour cells (Gottesman & Pastan, 1993, Ann. Rev. Biochem, 62, 385). The high incidence of *mdr1* and/ or *mrp* overexpression in patients who are relapsed or resistant to chemotherapeutic drugs underlies the clinical importance of controlling P-gp and MRP and we are using ribozymes (catalytic antisense RNAs; reviewed by Eckstein *et al*, 1996, Nuc. Acids. Mol. Biol., 10, *RNA catalysts*, Springer Verlag, Heidelberg) to target these genes.

We have designed and developed a novel and flexible strategy for the exogenous and endogenous synthesis of *trans*- acting asymmetric hammerhead ribozymes (StemIII 100-300 mer, StemI, 3 mer) addressing their effectiveness as potential therapeutic agents (Lewis & Gibson, 1996, Proc. of Int. Congress; Therapeutic Oligonucleotides, Rome, Abstract no. PR6, 108). Possible cleavage sites on the *mdr1* and *mrp* genes were selected in conserved sequences and/or regions wherein the mRNAs were predicted to have a low folding potential (Sczakiel *et al*, 1993, Antisense Res. Dev.,3, 453). Appropriate *mdr1* and *mrp* target sequences were amplified by RT-PCR using total cellular RNA templates extracted from parental (NIH H69/P; COR-L23/P) and resistant derivatives (H69/LX4; CORL23/R) of human small and large cell lung carcinoma cells, respectively. Using the *XbaI* and *EagI* sites incorporated on the PCR primers, the *mdr1* and *mrp* cDNA sequences were converted into a ribozyme construct by directional cloning into the pFORTH DNA cassette (Tabler & Tsagris, 1991, Gene, 108, 175). Ribozymes and substrate RNAs for *in vitro* analyses were transcribed from the vector-encoded T7 or T3 promoter, respectively, using Ampliscribe (Cambio). Cleavage products were assessed by electro-separation through denaturing gels (Separide, Life Technologies) or PAGE analysis. Ribozymes showing catalytic activity *in vitro* were subcloned into the mammalian expression vector, pHBApr1-neo (Gunning *et al*, 1987, PNAS, 84, 4831), for constitutive expression of the ribozyme under the B-actin promoter.

Cell lines were transfected with the ribozyme constructs (complexed with cationic lipids or polyamines) to assess their effect on the MDR phenotype *ex vivo*. Expression of the ribozymes in stably (500Ug/ml G418 selection) and transiently transfected cell lines were studied using Northern blotting, RT-PCR and toxicity assays.
This work is funded by Mrs. P. Salter.

### P58 REVERSAL OF MULTIDRUG RESISTANCE *IN VITRO* BY XR9051, A POTENT INHIBITOR OF P-GLYCOPROTEIN

W. Tuffley, K. Martin, I. Dale, M. Luscombe, P. Bevan, H. Ryder and P. Mistry. Xenova Ltd, Slough, SL1 4EF.

Expression of P-glycoprotein (P-gp) can confer multidrug resistance (MDR) on cancer cells and limit the effectiveness of many clinically important drugs. XR9051* is a potent inhibitor of P-gp, which restores drug sensitivity in a broad panel of murine and human P-gp overexpressing cell lines including examples where the resistance was intrinsic (e.g. MC26 murine colon carcinoma) or acquired following exposure to increasing concentrations of cytotoxic drug (e.g. EMT6 AR1.0 murine mammary carcinoma and H69/LX4 human lung carcinoma). Full reversal of resistance to several cytotoxic drugs, including doxorubicin, vincristine and taxol, was observed following 4-6 days coexposure to XR9051 (0.1-0.5 μM). In the drug resistant 1847AD human ovarian cancer cell line, P-gp expression results in reduced calcein-AM accumulation relative to the parental drug sensitive cell line. XR9051 reversed this accumulation deficit with an $EC_{50}$ of 0.07μM. Furthermore, in experiments designed to examine the duration of modulator action, a prolonged biological activity was observed following removal of XR9051 from the incubation medium. These studies in a number of cell lines demonstrated that P-gp dependent rhodamine-123 efflux was inhibited for several hours and that reversal of the [$^3$H] daunorubicin accumulation deficit was maintained for 22 h. These results suggest that XR9051 may not be transported by P-gp since two resistance modulators known to be P-gp substrates, cyclosporin A and verapamil, lost their inhibitory activity within 1 h of removal from the medium. Further studies on the mechanism of XR9051 action using [$^3$H]-azidopine, a photoaffinity label, demonstrated that XR9051 interacts with P-gp causing a concentration dependent inhibition of [$^3$H]-azidopine binding. These mechanistic studies suggest that XR9051 reverses drug resistance through a direct inhibition of P-gp function, but may not itself be a substrate.

In summary, XR9051 is a potent modulator of P-gp mediated MDR and may be beneficial in the treatment of MDR cancers. XR9051 has also been evaluated in several *in vivo* cancer models and Phase I studies in healthy volunteers are currently in progress.

*N-(4-(2-(6,7-dimethoxy-1,2,3,4-tetrahydro-2-isoquinolyl)ethyl)phenyl)-3-((3Z,6Z)-6-benzylidine-1-methyl-2,5-dioxo-3-piperazinylidene)methylbenzamide.

### P59 DIPYRIDAMOLE REVERSES VP16 RESISTANCE IN MULTIDRUG RESISTANCE-ASSOCIATED PROTEIN (MRP)-OVEREXPRESSING CELLS.

N.J. Curtin* and D. Turner. Cancer Research Unit University of Newcastle upon Tyne, UK

Multidrug resistance may be mediated by either P-glycoprotein (P-gp), the first identified plasma membrane drug efflux pump, or the more recently discovered, MRP. The nucleoside transport inhibitor, dipyridamole (DP), can reverse multidrug resistance in P-gp overexpressing cells (Shalinsky *et al Cancer Res.* **50** 7537: 1990). However, DP potentiated the cytotoxicity of VP16 in both wild-type and P-gp overexpressing cells to the same extent, suggesting this was not *via* P-gp (Turner and Curtin *Br.J.Cancer* **73** 856: 1996). This observation was explored using COR L23/P lung cancer cells and the MRP overexpressing drug-resistant variant, COR L23/R (Versantvoort *et al Br.J.Cancer* **72** 82: 1995)

In growth inhibition assays coincubation with 10μM DP potentiated VP16 and adriamycin 8- and 7.5-fold respectively but there was only a 2-fold potentiation of either drug in the parental cells. The resistant cells accumulated about 50% less VP16 and DP caused a modest time-dependent increase (1.5-2x) in both cell lines. Efflux of VP16 was initially very rapid, and faster in the resistant cells, but by 20 minutes only 20-30% of the original cellular concentration remained in both cell lines. DP slightly inhibited the rapid phase of VP16 efflux in the resistant but not the parental cells, but after 5 minutes the effect was similar in both cell lines.

Drug transport, and cytotoxicity, is regulated by cellular glutathione (GSH) in MRP-overexpressing cells, depletion of GSH by BSO potentiates vincristine and daunorubicin in COR L23/R cells[3]. DP lowered the GSH concentration significantly (24%) after 1 hour in the resistant cell line but only after 24 hours in the parental cells. The potentiation of VP16 and adriamycin cytotoxicity by DP may be mediated by GSH depletion

This work was supported by the NECRC (UK)

### P60 LOW LEVEL MULTIDRUG RESISTANCE EXPRESSION TO C1311, A POTENTIAL CLINICAL CANDIDATE.

J.R.Warr*[1], D.M. Quinn[1], J.A. Double[2,3] & M.C. Bibby[2,3], [1]Dept. of Biology, University of York, York, YO1 5YW, [2]Clinical Oncology Unit, University of Bradford, Bradford BD7 1DP, [3]Screening and Pharmacology Group, EORTC.

C1311 is a novel, water soluble, imidazoacridinone with potent *in vitro* activity against several colorectal and breast cancer cell lines and is also active *in vivo* at well tolerated doses against the corresponding solid tumours and is a candidate for clinical development. We have used a series of P-glycoprotein expressing KB multidrug resistant (MDR) cell lines to examine the extent to which the expression of the P-glycoprotein confers resistance to C1311. We have also examined the resistance of these cell lines to the structurally related compound, DUP-941, which is currently undergoing clinical trials. The low level MDR cell line, KB-8.5 (which is 3 fold resistant to doxorubicin, only expressed very slight resistance to C1311 (1.2 fold), but was 7 fold resistant to DUP-941. Two more highly resistant KB cell lines, KB-A1 and KB-C1, (which are 97 and 160 fold resistant to doxorubicin respectively), were 2.2 fold and 4 fold resistant to C1311 respectively. These two cell lines were 100 fold and over 100 fold resistant to DUP-941. Resistance to both drugs was reversible by verapamil. We have also made preliminary studies on the levels of resistance to these drugs shown by the MRP expressing MDR lung adenocarcinoma cell line, COR-L23/R, which indicated that this cell line is 4.7 fold resistant to C1311 and 10 fold resistant to DUP-941 respectively. Whilst the ultimate potential of C1311 can only be determined by clinical trials, these data add to the growing body of evidence that suggests it may have a significant advantage over the standard and investigational agents currently in use against breast and colorectal cancer.

P6. QUANTITATION OF RADIOLABELLED ANTIBODY DISTRIBU I ION IN TUMOUR BEARING NUDE MICE USING RADIOLUMINOGRAPHY.

*A. A. Flynn, A. J. Green, G. Boxer, J. Casey, R.B.Pedley,R.H.J. Begent. Department of Clinical Oncology, Royal Free Hospital School of Medicine, London, NW3 2PF.

Conventional dose estimates, based on the Medical Internal Radiation Dose (MIRD) formulation, assume uniform distribution of radionuclide within tissue. This assumption has been tested, in the case of radiolabelled antibodies directed against CEA, by using a statistical model to predict the pixel value distribution obtained from the digitised radioluminographs of a known radioactive source. The model is based on the statistical nature of the detection of radiation where any uniform source distribution can be expected to have a detected distribution that is Normal or Gaussian. We describe the novel use of such a test to assess the degree of uniformity of radionuclide distribution in tissue.

Radiolabelled anti CEA antibodies were administered to nude mice bearing LS174T colorectal cancer xenograft, and then sacrificed at 3, 24, 48 and 72 hours after injection. The uniformity of antibody distribution in tumour and normal tissues was measured using the radioluminographs of formalin fixed paraffin sections.

A normal distribution has a specific shape and is symmetric. Two statistical techniques have been used to test the shape and symmetry of the histogram of pixel values produced from the antibody distribution in a tissue section. Kurtosis and skew are measures of shape and symmetry and have statistically defined critical values for a normal distribution. The test statistic for kurtosis and skew was calculated for each tissue and compared with critical values from statistical tables (as shown in table below).

The radiolabelled antibody was found to be distributed uniformly in liver, spleen, muscle, lung and colon (insignificant kurtosis and skew values) and therefore allows conventional use of the MIRD formulation. The results showed that for kidney and tumour the kurtosis and skew values were significant and for bone the skew values alone.

| | Kurtosis | Skew |
|---|---|---|
| Bone | 0.7908 ($p>0.2$) | 0.508 ($p<0.01$) |
| Colon | 0.7936 ($p>0.2$) | 0.0734 ($p>0.1$) |
| Kidney | 0.8956 ($p<0.02$) | -0.23 ($p<0.01$) |
| Liver | 0.7936 ($p>0.2$) | -0.022 ($p>0.1$) |
| Lung | 0.7906 ($p>0.2$) | 0.1155 ($p>0.1$) |
| Muscle | 0.7916 ($p>0.2$) | 0.0856 ($p>0.1$) |
| Spleen | 0.8012 ($p>0.2$) | 0.0229 ($p>0.1$) |
| Tumour | 0.7549 ($p<0.02$) | 1.309 ($p<0.01$) |

This study demonstrates that for macroscopic absorbed dose calculations, kidney cortex and medulla should be considered separately, as should bone marrow and hard bone. Antibody heterogeneity in the tumour requires the incorporation of a microdosimetric tumour model for the accurate absorbed dose calculations.

A further potential application of this method is to evaluate the relative uniformity of radiolabelled antibody distribution within tumours, for intact antibodies and their fragments, to assist in the selection of antibody for radioimmunotherapy and other tumour targeting strategies.

P62 PRODUCTION OF A CHIMERIC ANTI-NCAM Fab' FOR USE IN TARGETING OF SMALL CELL LUNG CANCER

K.F., Eagle*, K.A., Chester, G.M.Boxer, R.B., Pedley, R.H.J., Begent. Department of Clinical Oncology, Royal Free Hospital School of Medicine, London, NW3 2PF.

Small cell lung cancer (SCLC) is a common disease accounting for 25% of all lung cancer. It is characterised by its innate chemo- and radiosensitivity but despite this, the 2 year survival is <5% due to the emergence of subsequent drug resistant disease. SCLC expresses a number of cellular antigens including the neural cell adhesion molecule (NCAM) which is expressed by all SCLC and therefore is a suitable target for antibody therapy. An intact murine $IgG_1$ antibody - NY.3D11 which recognises NCAM has previously shown specific uptake in nude mice bearing SCLC xenograft tumours. A recombinant chimeric Fab' (CFC) with Fvs derived from the monoclonal NY.3D11 and human CH1 and C kappa has been cloned. CFC was produced in *E. coli* culture supernatant and isolated by affinity chromatography and size fractionation. The purified CFC was tested for NCAM binding using ELISA and western blotting, demonstrating specific binding compared to a negative control which was a recombinant Fab' molecule (THFC) containing the NY.3D11 V kappa chain. FACS analysis showed specific binding of CFC only at a concentration of 50μg/ml but immunohistochemistry using both H69 cell lines and xenografts was negative. A biodistribution study in H69 xenografts showed no specific localisation with tumour:blood ratios of 1.2 and % injected dose of 0.3 at 24 hours. We conclude that, although a chimeric anti-NCAM Fab' was successfully engineered, there was no specific localisation *in vivo*. This may be due to a reduction in avidity by using a univalent Fab' or alterations in the murine framework regions in Vkappa which were made to allow forced cloning.

P63 RADIOIMMUNOGUIDED SURGERY (RIGS) WITH ANTI-CEA SINGLE CHAIN FV ANTIBODY IN COLORECTAL CANCER

*A. Mayer[1], K. Chester[1], G.Boxer[1], D. O'Malley[1], B. Davidson[2], M.A.M. Winslet[2], A.J.W. Hilson[3], R.H.J. Begent[1]. ([1]CRC Targeting and Imaging Group, Dep. of Clin. Onc., [2]Dep. of Surgery, [3]Dep. of Med. Physics, Royal Free Hospital MS, London NW3 2PF)

RIGS is based on the preoperative injection of a radiolabelled antitumour antibody and the intraoperative detection of radioactivity by a hand-held gamma detecting probe (Neoprobe). The slow blood clearance of whole IgG, which results in intervals of up to three weeks before tumour:blood ratios are high enough to permit accurate distinction of tumour tissue during surgery, has been disadvantagous. Single chain Fv antibody fragments (scFvs) consist of a variable heavy and a variable light chain region joined by a flexible linker and are the smallest antibody fragments to retain full binding capacity. The low molecular weight (27 kD) gives better tumour penetration and more rapid blood clearance than whole IgG resulting in high tumour:blood ratios at early time points.

MFE-23-his is an scFv with high affinity and specificity for CEA derived from bacteriophage technology. It was secreted as a bacterial supernatant using an *E.coli* expression system and was purified by immobilised metal ion affinity chromatography and gel filtration.

40 patients undergoing surgery because of primary or recurrent colorectal or any other CEA-expressing abdominal cancer will receive 1mg MFE-23-his labelled with $^{125}$Iodine to a specific activity of 185 MBq/mg 24, 48 or 72 hours prior to operation. After traditional exploration the abdomen is scanned with the hand-held probe. Results obtained by the probe are correlated with histology and counts obtained by a gamma well counter. Phosphorimaging is performed to determine the microdistribution of the antibody. Preliminary results showed selective tumour localisation in a patient undergoing resection of 2 liver metastases. This was confirmed by gamma well counting (tumour:normal liver - 3.3:1) and histology. RIGS-positive tissue was also recorded in the region of the celiac axis, the hepatoduodenal ligament and suprapancreatic aorta. Rapid excretion of the radiolabelled scFv was confirmed by a tumour to blood ratio of 4.4:1. A further two patients undergoing resection of primary colorectal cancer showed localisation of the antibody within their primary tumour. The results show that scFv antibodies can be prepared and used successfully for RIGS.

P64 99mTc LABELLING OF MFE-23 (AN ANTI-CEA SINGLE-CHAIN ANTIBODY)

D.A.Read*, M.S.Cooper[2], R.Boden, J.A.Boden, K.A.Chester & R.H.J.Begent. CRC Labs., Dept. Clin. Oncology & Dept. Pharmacy[2], Royal Free Hospital Shool of Medicine, London NW3 2PF.

The single-chain Fv antibody, MFE-23, radiolabelled with $^{123}$I, has been demonstrated to be an effective diagnostic imaging agent in patients with CEA expressing tumours (Begent *et al.* 1996, Nature Medicine **2**, 979). For routine use, the labelling of MFE-23 with $^{99m}$Tc rather than $^{123}$I may have several advantages ; Technetium is inexpensive, readily available for use in general hospital practice and is imaged efficiently by gamma cameras. In addition, direct labelling with $^{99m}$Tc to a residue away from the binding site is likely to be less damaging to antigen binding than the indescriminate iodination of tyrosine residues, some of which are sited in the CDRs.

A gly-ala-ser-(gly)$_4$-cys Tc chelating peptide (George *et al.* 1995, Proc.Natl.Acad.Sci.USA **92**, 8358) was fused to the carboxyl terminus of MFE-23. The scFv was expressed by *E.coli* and purified from culture supernatant by CEA affinity chromatography, before reduction by mercaptoethanol, aliquoting and storage in PBS at -70'C.

50μg thawed scFv was adjusted to pH 9.8 then labelled with 10MBq 99mTc "Gluheptoscint"(DuPont), a glucoheptonate based Tc labelling kit. TLC/phosphor imaging systems were used to assess % activity associated with colloid (3.0 %), glucoheptonate (68.6 %), free pertechnetate (8.6 %) and scFv (19.8 %). The *in vivo* distribution properties of the Tc labelled MFE-23 product were assessed in nude mice bearing LS174T (CEA producing) xenografts. 400kBq labelled product was injected iv/mouse.

The mean % injected activity per gram tumour at 24 hours was 0.67 with a tumour to blood ratio of 5.88. Normal tissue to blood ratios ranged from 0.31 to 2.03 (liver) with the exception of the kidney which displayed a tissue to blood ratio of 31.4.

Both circulating scFv and pertechnetate/glucoheptonate clear via the kidneys (reflected in high %inj. activities) whereas uptake in the liver is related to levels of colloidal Tc in the labelling product. Recent improvements in the methodology have reduced this colloidal Tc to <0.3%. This simple "one-pot" labelling method fulfills the requirement for the clinically- friendly production of a Tc labelled single chain antibody.

P65 **TUMOUR ANTIGEN GENE EXPRESSION IN DIFFERENT TUMOUR TYPES.** S. Rodgers[1], K. A. Mulcahy[1], K. E. Platts[1], C. A. McIntyre[1], R. C. Rees[2] and A. K. Murray[1]*.

[1]Institute for Cancer Studies, University of Sheffield Medical School, S10 2RX,
[2]Department of Life Science, Nottingham Trent University, NG11 8NF

It is well established that tumours can express antigens which distinguish theM from surrounding normal tissue. The MAGE, GAGE and BAGE gene families are silent in normal tissue except for the testes but expressed in tumours of different histological types. These antigens therefore represent potential targets for tumour directed therapy. To determine which patient groups would be potential candidates, the expression of MAGE, GAGE and BAGE genes in a variety of tumour types has been determined. These included head and neck squamous cell carcinoma, cutaneous malignant melanoma, lung carcinoma, prostate carcinoma and seminoma.

MAGE gene expression was assessed by reverse transcription followed by polymerase chain reaction amplification and ethidium bromide staining. Products were verified by Southern blots with digoxigenin-labelled oligonucleotide probes specific for each MAGE gene (Mulcahy K. A. *et al.*, (1996) *Int. J. Cancer* **66**:738). The results show that 6/39 head and neck tumours, 6/23 melanomas, 0/2 lung tumours, 12/37 prostate cancer and 2/5 seminomas express MAGE-1 MAGE-2 was expressed by 4/39 head and neck tumours, 7/23 melanomas, 0/2 lung tumours, 2/37 prostate cancers and 1/5 seminomas. MAGE-3 was expressed by 7/39 head and neck tumours, 7/23 melanomas, 1/2 lung tumours, 1/37 prostate cancers and 0/5 seminomas. MAGE-4 was expressed in 7/39 head and neck tumours, 1/23 melanomas, 1/2 lung tumours and 1/37 prostate cancers. GAGE and BAGE gene expression is currently being investigated using a similar technique.

Phase I clinical trials of MAGE peptides are currently underway in Sheffield in collaboration with the Ludwig Institute for Cancer Research (Brussels Branch). These data show that the MAGE gene family of tumour antigens are expressed in a variety of tumour types, some of which have not yet been reported in the literature, and so widens the patient groups for which targeted therapy may be appropriate as and when it become available in the future.

**This work was supported by the YCRC**

P66 **MUC1 MUCIN AS A TARGET FOR ANTIBODY MEDIATED ANTI-TUMOUR REACTIONS,** E. Petrakou*[1], G. Denton[1], A. Murray[1], R.A. Robins[2] and M.R. Price[1], [1]Cancer Research Lab., Dept. Pharm. Sci., [2]Dept. Immunology, Univ. of Nottingham, Nottingham

The MUC1 mucin is a high molecular mass transmembrane glycoprotein (>400kD) with lubricant and anti-infective properties in normal epithelia. In malignant cells, MUC1 expression is up-regulated, the molecules are incompletely/aberrantly glycosylated and normal cellular architecture is lost. There is increasing evidence that MUC1 expressed by tumour cells may provoke an anti-tumour immune response in the autochthonous host. We have therefore undertaken firstly to characterise the expression of MUC1 molecules on a panel of human breast tumour cell lines, and then determine whether the MUC1 mucin mav function as a target for complement dependent cytotoxicity as an example of an anti-tumour immune reaction.

MUC1 antigen expression on human tumour cells was assessed using both adherent cells and cells in suspension harvested by trypsinisation. Good concordance was obtained using these cell preparations in an evaluation of antigen expression by ELISA, FACS analysis and immunofluorescence. The cell lines T47D and MCF7-wt showed highest MUC1 expression, followed by MCF7-adr and MDA231.

The 4 cell lines were examined for their susceptibility to complement dependent antibody-mediated lysis using both murine IgG and IgM anti-MUC1 monoclonal antibodies which recognise determinants within the MUC1 mucin protein core. Only T47D showed any indication of complement dependent lysis using rabbit serum as source of complement. Antibody binding to MUC1 could be enhanced by treatment of cells with neuraminidase to remove sialic acid and render the protein core more accessible to antibody. However, this did not result in any marked increase in cytotoxicity.

It is possible that the inability of MUC1 mucin to function as an effective target for complement dependent cytotoxicity, despite high levels of antibody binding, is attributable to the extended rod-like conformation of the antigen. Antibody is bound, but is not in sufficiently close proximity to the lipid bilayer to initiate effective lysis.

P67

**Gene Transfer Therapies for Colorectal Cancer.** H. Chong, [1]R.M. Diaz, G. Hutchinson [1], I. Hart [1], N. Hardwicke, S. Castledon and R.G. Vile. ICRF Laboratory of Cancer Gene Therapy, Hammersmith Hospital, [1]ICRF/Richard Dimbleby Department, St Thomas' Hospital, London.

To develop protocols for the molecular immunotherapy of colorectal cancer we have used the poorly immunogenic CMT93 colorectal tumour line to compare the efficacy of different genes to treat primary tumours and/or to stimulate anti-tumour immunity. Simultaneously, we have developed efficient and rapid gene delivery systems.

Eradication of primary tumorigenicity was most effective with the HSVtk/Ganciclovir system. Even large tumours (~0.7 $cm^3$) could be cured with GCV treatment, consistent with the strong local bystander killing effects in this line. Primary tumorigenicity was also greatly reduced by expression of IL-12, B7.1, B7.2 or IL-2, all of which required T cells for the anti tumour effects. Expression of GM-CSF, or the cytosine deaminase genes were largely ineffective.

Killing CMT93 cells *in vivo* with HSVtk/GCV afforded some protection against parental cells. IL-2, GM-CSF or B7.2 were more potent but not completely effective. Therefore, we used combinations of genes to improve on these effects. Tumour cells expressing IL-12 and B7.1 or GM-CSF and B7.1 were optimal for generating systemic protection although the combination of GM-CSF and HSVtk also conferred some protection. Other cytokines are currently being tested.

To complement these studies, we have continued to develop protocols for the rapid transduction of primary tumour cells *ex vivo*. Previously, we described the use of 1st generation recombinant adenoviral vectors to obtain IL-2 gene expression in freshly resected colorectal cells at levels equivalent to those which are effective in our animal experiments in stimulating tumour cell rejection (~70ng IL-2/$10^6$ cells/48hrs) as little as seven days following surgery. We have now shown that the adenoviral system is consistently superior in terms of titre and speed of transduction to retroviral-based delivery systems (MFG-lacZ) in both primary colorectal and melanoma cells. In addition, experiments are underway using 2nd generation adenoviral vectors free of replicating virus expressing other cytokines (HSVtk, GM-CSF, IL-12, B7), alone or in combination, to demonstrate the feasibility of such approaches for clinical trials.

P68 BIODISTRIBUTION OF A5B7-F(ab')2-CPG2 CATALYTIC ACTIVITY IN XENOGRAFTED NUDE MICE FOR ADEPT. S.K.Sharma*, R.B.Pedley, J.A.Boden, R.W.Boden & R.H.J.Begent. CRC Labs., Dept. of Clinical Oncology, Royal Free Hosp. Sch. of Med., Rowland Hill St., London NW3 2PF.

The ADEPT approach of targeting enzymes to tumours, where they can convert a non-toxic prodrug to a cytotoxic molecule at the tumour sites, has shown feasibility as a treatment for cancer.

The bacterial enzyme carboxypeptidase G2 (CPG2), conjugated to an anti-CEA antibody fragment A5B7-F(ab')2, has been used extensively in our ADEPT pre-clinical and clinical studies. Biodistribution studies of radiolabelled conjugate have shown preferential localisation in the tumour, but the radioactivity does not indicate whether the localised CPG2 is active. Although an established spectrophotometric assay can measure enzyme activity in plasma using methotrexate as a substrate, it is not suitable for measurement of enzyme activity in tissues. A more sensitive HPLC assay for enzyme activity has therefore been developed in order to assess both the therapeutic potential and normal tissue toxicity of the conjugate. Nude mice bearing LS174T xenografts were injected with A5B7-F(ab')2-CPG2 (36units per mouse) and CPG2 activity measured in tumour and normal tissues at time points ranging from 1hr to 72hrs after conjugate injection. The % injected enzyme activity per gram of tumour at 24 and 72hrs was 8.9 and 1.4 respectively. The tumour to plasma ratio was 1.6 at 24hrs and 6 at 72hrs. The tumour to other normal tissue ratios ranged between 1.6-8 at 24 hrs and 2.4-11 at 72hrs after the conjugate injection. This shows the level of enzyme necessary for anti-tumour activity in mouse models and defines levels needed for therapy in man. Further experiments to investigate the effect of dose escalation of conjugate on CPG2 activity in both normal tissues and the tumour are in progress.

P69 **ZD2767, an improved system for ADEPT - investigation of different dosing regimes for the prodrug, ZD2767P.** S.J. East*, B.J. Curry, R.I. Dowell, D.H. Davies and D.C. Blakey. Cancer, Metabolism and Endocrine Research Dept., ZENECA Pharmaceuticals, Alderley Park, Macclesfield, Cheshire, SK10 4TG, UK.

ZD2767 is being developed as an improved system for antibody-directed enzyme prodrug therapy (ADEPT). It consists of a conjugate (ZD2767C) of the $F(ab')_2$ A5B7 antibody fragment and carboxypeptidase G2 (CPG2) and a bis-iodo phenol mustard prodrug (ZD2767P). Previously (Blakey D.C. et al, Cancer Research 56, 3287, 1996) we have described studies in which administration of ZD2767 to nude mice bearing human LoVo colorectal tumour xenografts resulted in major tumour regressions and prolonged growth delays. These studies have now been extended to investigate different dosing regimes for ZD2767P.

Mice bearing established LoVo tumour xenografts (approx. 6 x 6 mm) were administered with a single i.v. dose of ZD2767C (500 U/kg) followed 72 hr later by ZD2767P. Three different ZD2767P dosing regimes (single bolus injection, three doses at hourly intervals, 24 hr infusion using a mini-pump) were investigated. The dose of ZD2767P was chosen to give similar toxicity, as judged by body weight loss, and anti-tumour activity was assessed by measuring the size of the tumours and calculating growth delays compared to control groups. The results are summarised below:

| ZD2767P schedule | GD (days) | BWL (%) | N° of studies |
|---|---|---|---|
| Single bolus | 24 | 6 | 5 |
| 3 doses in 2 hr | 31 | 6 | 5 |
| 24 hr infusion | 20 | 7 | 2 |

(GD - Growth Delay, BWL - Body weight loss)

The best anti-tumour activity with ZD2767P was achieved when the prodrug was administered as 3 doses at hourly intervals. This regimen has been selected for initial clinical trials with ZD2767 in CEA positive cancer patients.

P70 MUTANT HUMAN RIBONUCLEASE FOR USE IN ANTIBODY-DIRECTED ENZYME PRODRUG THERAPY (ADEPT), D. C. Blakey*[1], F. T. Boyle[1], D. H. Davies[1], R. I. Dowell[1], D. W. Heaton[1], M. S. Rose[1], A. M. Slater[1], H. J. Eggelte[2], A. Tarragona-Fiol[2], C. J. Taylorson[2] and B. R. Rabin[2], [1]Zeneca Pharmaceuticals, Alderley Park, Macclesfield, Cheshire, SK10 4TG, [2]University College London, London, WC1E 6BT.

ADEPT is a two step antibody based targeting strategy for the treatment of cancer. We have initially focused on developing ADEPT systems incorporating the bacterial enzyme carboxypeptidase G2 (D. C. Blakey et al, Cancer Research, 56, 3287, 1996). These systems utilise an enzyme component which is immunogenic and this will limit the courses of therapy that can be given in the clinic. In an attempt to overcome this limitation we have been developing an ADEPT system incorporating a mutated version of human ribonuclease in which the substrate binding pocket has been altered to change substrate specificity. This involved mutating a positively charged lysine residue at position 66 in human ribonuclease to a negatively charged glutamic acid residue. A positively charged uracil based prodrug was synthesised which releases, by the action of mutant ribonuclease, the potent dichlorophenol mustard drug. This prodrug was shown to be a substrate for Lys66Glu mutant human ribonuclease ($k_{cat}/Km$ = 3.6 $mM^{-1}$ $s^{-1}$). Native human ribonuclease cleaved the prodrug approximately 20 fold slower ($k_{cat}/Km$ = 0.2 $mM^{-1}$ $s^{-1}$). The uracil based prodrug was approximately 30 fold less cytotoxic than the corresponding dichlorophenol mustard drug ($IC_{50}$ approximately 1 μM) when incubated with LoVo colorectal tumour cells. These studies demonstrate the potential to reverse the polarity of human ribonuclease for use in ADEPT.

P71 SENSITIVITY OF COLORECTAL CELL LINES TO THE NOVEL ADEPT AGENT ZD2767: THE ROLE OF CELLULAR GLUTATHIONE. N.R.Monks*[1], D.R.Newell[1], J.A.Calvete[1], S.J.East[2], D.C.Blakey[2]. [1] Cancer Research Unit, University of Newcastle, Newcastle upon Tyne, NE2 4HH. [2] Dept of Cancer, Metabolism and Endocrine Research. Zeneca Pharmaceuticals. Alderley Park. Cheshire. SK10 4TG.

ZD2767P is a novel phenol mustard glutamate prodrug which has been developed for use in ADEPT, (Blakey *et al. Cancer Research.* **56**:3287 (1996). ZD2767P (4-[*N,N*-bis(2-iodoethyl)amino]phenoxy carbonyl-L-glutamic acid) is cleaved to the active bifuntional alkylating agent (4-[*N,N*-bis(2-iodoethyl)amino] phenol, ZD2767D) by the action of the bacterial enzyme carboxypeptidase G2. The aims of these investigations were to determine the sensitivity of a panel of six colorectal cell lines (HT29, LoVo, LS174T, HCT15, SW620 and HCT116) to ZD2767D and P using the sulphorhodamine B staining growth inhibition assay, and the role of glutathione (GSH) as a determinant of cellular sensitivity. The $IC_{50}$ values for ZD2767D and P, ranged from 0.06-1.4μM and 8-200μM respectively. In all cell lines ZD2767D was 100-200 times more potent than ZD2767P. Chlorambucil demonstrated a similar range of sensitivities ($IC_{50}$ 26-200μM) with the cell lines that were at the extremes being the same as for ZD2767D and P (most sensitive LS174T, least sensitive HT29). Intracellular GSH concentrations, determined by HPLC varied between 1.3-12.5 nmoles/$10^6$ cells, but did not correlate with cellular sensitivity to ZD2767D, P or chlorambucil. Depletion of GSH using a non-toxic concentration of butathionine sulfoximine (10μM), resulted in an 80% reduction in intracellular GSH, although this was found to only increase cell sensitivity by a maximum of 2.3-fold. These studies have demonstrated that GSH is not a major determinant of inherent chemosensitivity to ZD2767 in six colorectal cell lines.

P72 **CHARACTERISATION OF THE *MYCN* AMPLICON IN NEUROBLASTOMA**

*RM Kenyon, RE George, AG McGuckin, ADJ Pearson, [2]P Kogner, [3]H Christiansen, J Lunec. Cancer Research Unit, Univ. of Newcastle upon Tyne, Newcastle upon Tyne, NE2 4HH, UK, [2]Childhood Cancer Research Unit, Karolinska Institute, Stockholm, Sweden, [3]Univ. Kinderklink, Giessen, Germany.

Neuroblastoma is a paediatric solid tumour with a varied clinical course. One genetic feature used as a poor prognosis marker for patients is the amplification of the *MYCN* gene, which is found in 25% of primary tumours. However, patients whose tumours exhibit this feature are not all seen to have a poor survival. We therefore investigated the co-amplified DNA surrounding the *MYCN* gene which potentially includes other genes, that may influence the biology of these tumours.

Two sequence tagged sites (STSs) were isolated from a yeast artificial chromosome (YAC) clone containing the *MYCN* gene. These lay 70Kb 5' and 3' of the *MYCN* gene and were used as DNA probes in a Southern blot analysis to asses the co-amplification patterns of outlying sequences in neuroblastoma primary tumour and cell line samples. A panel of 35 *MYCN* amplified primary tumour samples were studied. The STS probes identified a subset of primary tumours that showed amplification of the 3' STS, but not the 5' STS, with the *MYCN* gene, while the majority of samples were seen to amplify both the 3' and 5' STSs. When tested by Log-rank survival analysis, this subset of patients was seen to have a significantly longer progression-free interval than those patients that were seen to amplify both STSs in their tumours (p= 0.036). This association was independent of the stage of the tumours and the number of copies of the *MYCN* gene. The STSs also identified a difference in the region amplified between cell lines and primary tumour samples with cell lines amplifying the 5' STS, but not the 3' STS; a pattern not seen in primary tumours. This suggests a selection pressure for the amplification of the region 5' of *MYCN* in cell culture, which could be provided by another co-amplified expressed gene.

The amplification status of three genes known to map to the same chromosomal region as *MYCN* was analysed. The number of copies of the genes for Adenylate cyclase 3 and thyroid peroxidase was indistinguishable from the normal diploid complement in these tumours. The *N-cym* gene, which occurs on the antisense strand opposite the *MYCN* gene was found co-amplified in 16/17 tumours. One tumour was identified that did not amplify exon three of the *N-cym* gene, placing the start of the amplified region within 0.5Kb of the start of exon 1 of the *MYCN* gene. This redefines the "core region" (the smallest region commonly amplified in all neuroblastoma *MYCN* amplified tumours) reported by Reiter *et al*, 1996, Genomics, *32*; p97.

### P73 ALTERATIONS OF P53 AND MDM2 IN TRANSITIONAL CELL CARCINOMA (TCC) OF THE BLADDER,

R. Abdel-Fattah*[1], I. Sigalas[1], L.Griffiths[2], D. Neal[2], J. Lunec[1], [1]Cancer Research Unit, [2]Dept. of Surgery, University of Newcastle upon Tyne, Newcastle upon Tyne, NE2 4HH.

Genetic alterations in the p53 gene have been reported as frequent events in bladder cancer and are associated with disease progression. The mdm2 gene is a target for transcriptional activation by p53 and the MDM2 protein binds to p53 , acting as an autoregulatory feedback inhibitor of p53 transcriptional function. The aims of this study were to: i) determine and relate mdm2 and p53 abnormalities in bladder tumours and ii) examine the clinical relevance of altered patterns of expression in bladder cancer. 74 cases of TCC were assessed by immunohistochemistry (IHC) for p53 using DO7 antibody. Exons 4 to 9 of p53 were sequenced in 32 frozen tumours. Expression of the mdm2 gene was studied by IHC (NCL-MDM2 Clone 1B10, Novocastra Laboratories) and RT-PCR. Focal (>20%) and heterogeneous (<75%) MDM2 staining was detected in 47% of tumours and was associated with Ta disease. No association was found with grade and p53 staining. Diffuse (>75%) MDM2 staining was detected in 12% of tumours, none of which had p53 mutation. MDM2 overexpression in tumours that did not show p53 mutation suggested that binding by mdm2 might be an alternative way in which p53 function is abrogated in these tumours. At short-term follow-up, five T1 tumours progressed. Diffuse MDM2 and diffuse p53 staining was detected in all five tumours ($P$=0.03, Fisher's). Our studies revealed different patterns of multiple-sized RT-PCR products in 11/39 (28%) tumour samples when amplified for the complete coding region of mdm2. There was a higher incidence of alternatively spliced forms in 8/15 (53%) late stage (T2-T4) compared with 3/24 (12.5%) early stage (Ta-T1) tumours, suggesting an association with tumour progression. Studies are in progress to investigate the prognostic and functional significance of these forms of mdm2.

### P74 DNA BINDING BY THE HUMAN MDM2 ONCOPROTEIN.

C.Challen[1], I. Mitrou[1], J.J.Anderson[2] and J.Lunec[1]. Cancer Research Unit, University of Newcastle upon Tyne Medical School, England, NE2 4HH. [2]Dept of Pathology, RVI, Newcastle Upon Tyne, England, NE2 4HH.

The MDM2 oncogene encodes a 90 kilodalton nuclear phosphoprotein that binds and inactivates the p53 tumour suppressor gene.We have previously characterised alternatively spliced MDM2 transcripts which retain transforming ability despite the loss of sequences from the 5' end of the gene that encode the p53 binding domain (Sigalas I, et al, Nature Medicine 1996 2:912-917). Although the C-terminal region of MDM2 includes an acidic domain together with zinc and Ring-Finger regions, suggesting a DNA binding and transcription factor function, these properties have yet to be shown. We have examined the DNA binding properties of both full length and C-terminal fragments of the MDM2 protein. Gel retardation analysis confirmed the ability of full length and C-terminal regions of MDM2 to bind DNA from a pool of random sequences. Furthermore, sub-pools of DNA could be selected from this random mixture for which the C-terminal region of MDM2 had increased binding ability, shown by gel retardation assays, indicating some degree of selective binding. Characterisation of the DNA binding by MDM2 showed that increased concentrations of EDTA (100mM), DTT (1mM-10mM) or β-mercaptoethanol (2mM-10mM) decreased the efficiency with which MDM2 bound DNA. Heat denaturation completely abolished the binding. Current studies are aimed at the cloning of DNA sequences for which MDM2 shows enhanced binding and establishing whether there is a consensus binding sequence for MDM2.

### P75 THE MAPPING OF INTRON-EXON BOUNDARIES IN THE HUMAN *MDM2* ONCOGENE AND IDENTIFICATION OF A POSSIBLE P53 PROMOTER REGION IN THE INTRON UPSTREAM TO EXON 3,

**H. Liang,*I. Sigalas, C. Challen, J. Lunec, Cancer Research Unit, University of Newcastle upon Tyne, NE2 4HH, U.K**

We hypothesised that the five alternatively sized transcripts of the human *mdm2* oncogene (*mdm2* a-e) with deletion of internal sequences in human tumour samples (Nature Medicine 1996 2, 912-917) resulted from an alternative splicing mechanism and that the junctions of the deleted region corresponded to the boundaries between introns and exons. To test this hypothesis, sequence analysis of long range PCR products of human genomic DNA across these putative splice junctions has been carried out.

| Exon no. | Sequence of boundary | Exon no | Intron size (kb) |
|---|---|---|---|
| | GCAGgtgctgg---------ccttgtagGCAA<br>307 308 | 3* | ~1.2 |
| 3* | CCTGgttagtat----------tcttatagGTTA<br>392 393 | 4* | ~6 |
| 4* | AGAGgtaagctg---------gatttcatGTTC<br>467 468 | 5* | ~5 |
| 9* | TCCGgtaatgtt---------- tgttttagGATC<br>977 978 | 10* | ~8 |
| 11* | AGCTgtagtata---------cattgaagGACT<br>1211 1212 | 12* | ~5 |

The figures underneath the underlined nucleotides denote their cDNA sequence number.

*: The exon numbers are denoted by homology with the corresponding exons of mouse *mdm2*, since the full human intron-exon map has not been produced. Our data indicates that the equivalent of exon 3 of the murine gene should be designated exon 2 in human *mdm2*.

The result shown in the table has confirmed that three transcripts (*mdm2* a-c) with missing nucleotide 393 to 977, 393 to 1211 and 468 to 977 are generated by multiple exon skipping. However, the remaining two transcript variants (*mdm2* d and e) missing nucleotide 400 to 1476 and 536 to 1759 have neither intron nor consensus splicing sequences at the deletion junctions, suggesting that these two transcripts result from either very unusual splicing or partial gene deletion.

Interestingly, the 3' end of the intron upstream to exon 3* has a TATA box and several motifs which show a good match to consensus p53 binding sequence. We are investigating whether there is a p53-dependent promoter in this intron.

### P76 THE INFLUENCE OF MICROSOMAL EPOXIDE HYDROLASE GENETIC POLYMORPHISMS IN COLORECTAL CANCER SUSCEPTIBILITY.

K-J. Palmer , A.C. Gough, C.A.D.Smith and J.N. Primrose. University Department of Surgery, Southampton General Hospital, Tremona Road, Southampton, Hampshire, SO16 6YD.

Human cancer risk has been associated with exposure to pro-carcinogens. The Epoxide Hydrolases are a family of at least four enzymes which play an important role in the detoxification of mutagens, carcinogens and other xenobiotics. One of these, microsomal epoxide hydrolase (mEH), has been shown to be polymorphic. Two specific sites within the gene have been associated with alterations in activity. An increase in activity has been associated with a mutation in exon 4 and a decrease in activity associated with a mutation in exon 3. (C.Hassett et al, Human Molecular Genetics, 1994, vol 3(3):421-428) As epidemiological studies have suggested that colorectal cancer is linked with exposure to dietary and environmental carcinogens, it is the aim of this study to ascertain the influence of polymorphisms within the mEH gene on colorectal cancer susceptibility.

Genomic DNA was extracted from colorectal cancer mucosae (n=58) and random unaffected individuals (n=72). Polymerase Chain Reaction (PCR) was performed using specific oligonucleotide primer sequences designed to detect the genetic polymorphism in exon 4 of the mEH gene, which results in an amino acid substitution at position 139 (His/Arg). PCR products were analysed by restriction digestion with the restriction endonuclease RsaI, polyacrylamide gel electrophoresis and visualised by ethidium bromide staining under ultraviolet transillumination.

Of the 58 colorectal cancer samples genotyped at position 139, 1.7% were homozygous $Arg^{139}/Arg^{139}$, 25.9% heterozygous $Arg^{139}/His^{139}$ and 72.4% homozygous $His^{139}/His^{139}$, whereas the random population frequencies were 5.6%, 40.2% and 54.2% respectively. Statistical analysis showed that there was a significant difference between the two populations.($\chi^2$ Yates correction 0.05>P>0.01).

In conclusion, these preliminary results suggest that the exon 4 polymorphism in mEH may influence colorectal cancer susceptibility. The exon 3 mutation at position 113 has been suggested to lower the activity of mEH by the replacement of a tyrosine with a histidine. The genetic analysis of this site in conjunction with the exon 4 site is in progress.

P77 CYTOGENETIC AND MULTIPLE CHROMOSOME PAINTING STUDIES OF HUMAN LYMPHOMA CELL LINES.

Mohammed Alqahtani[1]*, David W.Hammond[1] and Malcolm H. Goyns[2]. [1]Department of Clinical Oncology, Y.C.R.C. Institute for Cancer Studies, Sheffield University Medical School, Beech Hill Road, Sheffield, S10 2RX; [2]School of Health Sciences, University of Sunderland,Whamcliffe Street, Sunderland, SR2 3SD, U.K.

The use of cell lines can be of considerable importance in studying the genetics of cancer. However, the detection of chromosome abnormalities in malignant cell lines by conventional cytogenetics is particularly problematic because of the difficulty in routinely preparing metaphase spreads of sufficient quality or quantity and because of the complex nature of many of the chromosomal changes. Fluorescence in-situ hybridization (FISH) is a powerful tool for improving the analysis of malignant cell chromosomes (Hammond et al., Ann. Oncol., 1994, 5 suppl.1, 51-54) and we have therefore adopted this approach in our studies of lymphoma cells. In this study, we have analysed five human lymphoma cell lines (Daudi, HS 602, Raji, MC116 and Namalwa). Some of these were partially analysed cytogenetically in the early 1970s using conventional staining techniques, but no chromosome analysis had been previously attempted on the Raji and HS 602 cell lines. Initially we completed the cytogenetic analysis of each of these cell lines using the G-banding technique. We then refined these preliminary findings by applying multicolour FISH analysis with whole chromosome paint probes for all twenty three human chromosome types using direct and indirect combinatorial labelling with two fluorochromes (FITC and Texas Red). In this way we were able to simultaneously visualise multiple target chromosomes in the same cell. These studies are still in progress, but they have already enabled complex rearrangements such as an ins(1p), ins(3p) and dup(1p)to be characterised and made the identification of marker chromosomes e.g. as a t(6q;7q) possible.

M. A. acknowledges a Government of Saudi Arabia studentship. This work was supported in part by the Yorkshire Cancer Research Campaign.

P78 ALU-PCR FINGERPRINTING ANALYSIS IN PROSTATE CANCER.

H. Y. Leung, R. Brown, A. B. McKie, N. R. Lemoine, C. N. Robson and D. E. Neal., Dept. of Surgery, Newcastle upon Tyne NE2 4HH

Genetic events related to the development of human prostate cancer are under intense investigations using conventional methods including loss of heterozygosity, microsatellite instability and linkage analysis. We describe the use of *Alu*-repeat based PCR DNA fingerprinting to scan genomic DNA from prostate cancer for identifying novel prostate cancer related genes. *Alu*-specific primers have been designed for PCR analysis on paired genomic DNA (tumour and blood) from ten patients with locally advanced prostate cancer. Control samples from a normal prostate (organ donor) and a normal testes were included. Two sub-clones from $SV_{40}$ transformed prostatic epithelial cell lines (PNT1) were also examined. Five *Alu*-specific primers were assessed. Genomic DNA were subjected to restriction digest prior to PCR analysis to reduce the number of band products and four endonucleases were assessed.

The condition for *Alu*-PCR was optimised. Two restriction digests were found to be most useful. Similarly, three *Alu*-specific primers were particular informative. Differences were seen between the two sub-clones of the PNT1 cells. In prostate cancer, different band profiles were noted between tumour and normal DNA and four bands of interest (three from tumour tracks and one form blood track) were identified and cloned for further characterisation.

*Alu*-based PCR DNA fingerprinting have been successfully applied in prostate cancer and may facilitate further study to identify novel prostate cancer related gene.

P79 INVESTIGATION OF GENES WHICH EXHIBIT DIFFERENTIAL EXPRESSION DURING AGEING, AND THEIR RELEVANCE TO THE IMMORTALISATION OF TUMOUR CELLS.

M.A. Charlton[1,2]*, M. Salehi[1,2], B.J. Merry[3] and M.H. Goyns[1], [1]Molecular Gerontology Unit, School of Health Sciences, University of Sunderland, Wharncliffe Street, Sunderland, SR2 3SD, [2]Institute for Cancer Studies, University of Sheffield Medical School, Beech Hill Road, Sheffield, S10 2RX and [3]Institute for Human Ageing, University of Liverpool, P.O. Box 147, Liverpool, L69 3BX.

Malignant transformation of cells produces a number of differences in phenotype compared to their non-malignant counterparts. One such change, which is poorly understood, is the suggestion that tumour cells may undergo immortalisation so that they no longer exhibit signs of senescence. To study which genes might be important in this process, we have studied changes in gene expression that occur during ageing of adult mammalian tissues as a means of identifying putative immortalising genes.

In this study we have used the PCR-based technique of differential display (Liang & Pardee, *Science*, 1992, **257**, 967-971) to analyse changes in gene expression during ageing of the brains and livers from CFY rats. This colony of animals has a maximum life span of 35 months when fed ad libitum, but few animals reach this age, and the LD50 of the colony has been 23 months for the past 20 years. In this study we have compared three sexually-mature young adults (6 months) with three old adults (22 months). RNA preparations were initially prepared from homogenised brains and subjected to RT-PCR using 40 different combinations of primer pairs. The comparison of three young to three old animals ensured that we reduced the risk of scoring artefacts on the differential display gels. Over 2,000 PCR products were identified from brain mRNA, of which 40 appeared to represent genes that exhibited differential expression during ageing (Salehi et al., *Experientia*, 1996, **52**, 888-891). Since this preliminary study, we have greatly improved both the resolution and reproducibility of the differential display gels. As a result, a combination of 30 primer pairs now enables up to 9,000 PCR products to be revealed for each tissue, of which some 200 appear to represent genes which are differentially expressed. This approach has now been applied to a study of the liver and rat hepatoma cell lines. We are currently characterising these PCR products, but have already observed that the *fos* proto-oncogene, which has previously been implicated in the immortalisation of cells, is down-regulated during the ageing of all tissues so far studied.

MAC acknowledges a Yorkshire Cancer Research Campaign studentship. MS acknowledges a Government of Iran studentship.

P80 ACTIVATION OF TELOMERASE DURING HEPATOCELLULAR CARCINOGENESIS.

M. Miura*[1], K. Higashi[2], K. Ikemura[1] and S. Gotoh[2], [1]Dept. of Oral Surgery, [2]Dept. of Biochem.,Univ. Occupational and Environmental Health. Kitakyushu City 807, JAPAN

Telomerase is required for the maintenance of telomere length during chromosome replication. Activation of telomerase has been associated with human cancers and immortal cells.

To study the telomerase activity , we first, improved the original TRAP assay ( Kim et al., 1994, Science, 266, 2011 ), by modifying the labelling method and the designs of the primers. With these modifications, the assay is more sensitive and more quantitative.

With our improved TRAP assay, we wanted to study on the dynamic process of upregulation of the telomerase activity during in vivo carcinogenesis. Rats were administered with a potent hepatocellular carcinogen, 3'-Me-DAB for 8 weeks. Every week after the first day of the administration, rats were sacrificed and the telomerase activity in liver homogenates was assayed . The augmented telomerase activity was detected a couple of weeks after the initiation of 3'-Me-DAB administration , before the appearance of hyperplastic nodules. On the contrary, the specimens of normal control liver showed weak telomerase activity.

These results suggest that activation of telomerase is an early event in hepatocellular carcinogenesis and may be a characteristic feature of precancerous hepatocellular lesions.

P81 **p16$^{INK4A}$ POINT MUTATIONS LEAD TO LOSS OF CDK4 INHIBITORY ACTIVITY,** L. Betts*, R.M. Haigh+, N. Redemann and J.C.A. van Meel, Oncology Research, Boehringer Ingelheim Pharma Germany, 88397 Biberach, FRG, +present address: Ferring Research Ltd, Southapton S016 7NP, UK

In many tumours, the INK4 gene is homozygously or heterozygously deleted leading to a loss of p16 which contributes to the tumourigenic process and has led to p16 being recognized as an important tumour suppressor. Recently, a number of point mutations in p16 have been detected in pancreas carcinomas. We constructed two of these mutatations, G23N (first ankiryn repeat) and H83Y (third ankiryn repeat), by site-directed in vitro mutagenesis in order to examine their functional effects on CDK4 kinase activity in comparison to wild type p16. Using retinoblastoma protein as substrate, results showed that 1 μg of p16 protein was able to inhibit fully cyclin D1/CDK4 activity whereas the same amount of both mutants had no effect. The inhibition by wild type p16 was concentration-dependent with a mean IC50 of 0.17 $\pm$ 0.07 μg/ml (SEM of 3 experiments). For mutant G23N there was a 400-fold increase in the IC50 and for H83Y over a 500-fold increase indicating that both are significantly less able to inhibit cyclin D1/CDK4 activity. Since p16 must first bind to the CDK4 complex to inhibit its activity, we further investigated whether the mutants could bind to CDK4. Wild type p16 or mutants attached to glutathione-Sepharose beads through a GST tag were incubated with cyclin D1/CDK4 and specifically bound complex was assayed by immunoblotting. Wild type p16 clearly formed stable complexes with cyclin D1/CDK4 whereas in contrast using the two mutants binding above non-specific background was not detected. It is concluded that p16$^{INK4A}$ point muatations can lead to a loss of inhibitory activity which may contribute to tumourigenesis in pancreatic adenocarcinoma.

P82 CDK4 AND CDC2 EXPRESSION ARE DIFFERENT IN HUMAN FIBROBLASTS AND KERATINOCYTES, S. Moumtzi*[1] and H.M. Warenius[1], [1]Department of Medicine, University Clinical Dept Daulby Street, Liverpool, L69 3GA.

In vitro cell culture systems provide the opportunity to analyse the growth requirements and behaviour or normal cells, to compare the properties of normal cells and cancer cells and to develop quantitative and reproducible assays for neoplastic cellular transformation.
The ultimate purpose of this work is to compare and identify the differences in the expression of proliferation related genes between transformed and normal keratinocytes and fibroblasts. We have examined four primary keratinocytes cultures in low calcium and four primary fibroblasts cultures from human skin of 8 different individuals. The fibroblasts grew rapidly (doubling time 2.86 days) in DMEM + FCS 10%. Whereas, the keratinocytes grew slowly (doubling time 4.75 days) in MCDB 153 + EGF medium. Initial studies involving Western immunoblotting for the two cyclin dependent kinases cdc2 and cdk4 showed a strong relationship between these proteins in fibroblasts (r=0.96, p=0.04) but not in keratinocytes (r=-0.66, p=0.34). Previously in our laboratory we have shown that in a wide range of human cancer cells there was a strong relationship between cdc2/cdk4 as observed in the normal fibroblasts reported here. Many of these cancer cell lines were of epithelial origin, however, suggesting cdk4/cdc2 relationships might be changed by malignant transformation. We are now undertaking transfection studies of normal keratinocytes to investigate this possibility.

P83 **EXPRESSION AND PRODUCTION OF TGF-β IN EPITHELIAL CULTURES DERIVED FROM NORMAL BREAST AND BREAST TUMOURS,** A.R. Green* and V. Speirs, Department of Medicine, University of Hull, Hull HU6 7RX.

A role for TGF-β as a mediator of cell proliferation and differentiation in both normal breast and breast cancer has been proposed. However, most previous reports have not classified between the three human isoforms of TGF-β and the expression/production of these have not been fully investigated. Using a series of primary epithelial cultures derived from normal breast (n=6) and breast tumours (n=9), we have studied the expression and production of TGF-β1 and β2 and the expression of TGF-β3. Epithelial cells were isolated from collagenase dispersed tissue by differential centrifugation followed by culture in selective media. Total RNA was extracted from cell cultures and analysed for TGF-β1, β2 and β3 mRNA transcripts by RT-PCR. Furthermore, CM was collected from the same cultures prior to RNA extraction and analysed for TGF-β1 and β2 protein by ELISA. Constitutive expression of TGF-β1 mRNA was observed in all cultures irrespective of origin whereas TGF-β2 and β3 were less commonly expressed with one culture from each population failing to express either isoform. Production of TGF-β1 and β2 by these cultures is summarised below:

**Table 1** Production of TGF-β1 and β2 by primary epithelial cultures derived from normal breast and breast tumours (pg/ml/μg RNA)

| | TGF-β1 | TGF-β2 |
|---|---|---|
| Normal | 4.9 - 66.3 (mean = 26.9) | 0.7 - 15.1 (mean = 6.1) |
| Tumour | 6.6 - 41.7 (mean = 20.9) | 2.1 - 7.0 (mean = 3.1) |

Production of TGF-β1 corresponded with mRNA expression in all cases, however TGF-β2 mRNA and protein did not always agree. There were no significant differences in expression of TGF-β mRNA or protein between the epithelial cultures derived from normals or tumours. However secretion of TGF-β2 was on average 4-7 fold lower compared with TGF-β1 in both sets of cultures. This study confirms that all three human isoforms of TGF-β are expressed by epithelial cells of neoplastic origin together with those derived from normal breast and it is likely that differential expression and regulation between these three isoforms may occur.
***Supported by Yorkshire Cancer Research Campaign***

P84 THE EFFECT OF 12-LIPOXYGENASE OVER-EXPRESSION IN THE HUMAN BREAST CANCER CELL LINE MCF-7 ON CELL GROWTH, APOPTOSIS, AND THE BCL-2 PROTEIN. Jeanne M. Connolly* and David P. Rose, American Health Foundation, Valhalla, NY 10595, USA.

The human breast cancer cell line MCF-7 grows slowly both *in vitro* and as solid tumors in nude mice *in vivo*. We have previously reported the successful transfection of 12-lipoxygenase (12LOX) into this estrogen receptor-positive cell line, which results in high secretion of 12-hydroxyeicosatetraenoic acid (12-HETE) and enhanced cell growth both *in vitro* and *in vivo* (Liu *et al.*, Cancer Letters, *in press*, 1996). One mechanism which can result in enhanced cell accumulation is reduced apoptosis. Our current investigations show that soluble nuclear matrix protein (NMP) levels, released by dead and dying cells and detected by ELISA in conditioned medium of MCF-7/12LOX cell cultures, are significantly lower than those in identically treated MCF-7 cultures (59.7±12.9 *vs* 100±6.7% of MCF-7 NMP U/ml, p<0.001), which suggests a reduced rate of programmed cell death in the 12LOX transfectants. In addition, levels of the bcl-2 proto-oncogene protein, an inhibitor of apoptosis, are elevated in the 12LOX transfected cell line compared to the MCF-7 parent as detected by Western blot. These data suggest that endogenous overexpression of 12LOX results in reduced apoptosis in these human breast cancer cells, and may contribute to enhanced cell growth and tumor development.

P85 **NAVELBINE (NVB) & FRACTIONATED DOSE DOXORUBICIN (DX) IMPROVES 1ST LINE ADVANCED BREAST CANCER (ABC). AN OVERVIEW OF 3 PHASE II TRIALS.**

J. Carmichael[1], R. Hegg[2], D. Firat[3], M. Pawlicki[4], F. Le Bras[5], F. M. Delgado[5], P. Danel[5] and S.A.N. Johnson[6]. [1]Nottingham City Hosp UK; [2]ELAN Brazil; [3]Ankara, Turkey; [4] Krakow-Poland; [5]I.R.P.F., France; [6]Taunton & Somerset Hosp, UK

Anthracycline combinations represent the most powerful chemotherapeutic approach in the treatment of ABC inducing objective response in 40%-70% of patients (pts) and halting progression in the majority of the others. The limiting toxicities of anthracyclines are neutropenia and cardiac impairment. NVB as a single agent has demonstrated a high activity in the treatment of ABC, the overall response rate (RR) ranging from 40% to 60% with good tolerance by pts. Impressive results have previously been obtained with NVB 25 mg/m² on days 1 & 8 + DX 50 mg/m² on day 1, every 21 days. Of 89 pts, 74% responded to the therapy (21% CRs),. and high RRs were observed in visceral metastatic sites: liver 50% (13% CRs), lung 68% (21% CRs) (Spielmann JCO 1994). A significant survival advantage was observed in pts with liver metastases treated NVB + DX compared to CAF (Blajman, ESMO 1996). Concerning DX there is evidence that dividing the dose and administering it at weekly intervals may reduce the cardiac toxicity without substantially impairing the efficacy (Chlebowki R, CTR 1980). Based on this rationale 3 Phase II studies were conducted in order to check the efficacy, improve the tolerance of NVB+DX in combination and to make out patient administration easier. 1[illegible] Chemotherapy naive ABC pts were treated with NVB and DX, both at 25 mg/m² IV on days 1 & 8 every 21 days, for a maximum of 8 cycles. Pts characteristics were: age from 30-73y; PS 0-1: 85%; visceral involvement: 52%; adjuvant chemotherapy: 18%. 668 cycles were administered; at least one episode of WHO grade (G) 3-4 neutropenia was observed in 24% of pts; the incidence of infection episodes was very low (G 3: 6/120 pts); G 3-4 nausea/vomiting was seen in 17 pts; only 1.5% of patients experienced G 3-4 constipation; G 1 peripheral neuropathy was observed in 13% pts. 53.5% of pts developed grade 3 alopecia. No life threatening (G 3-4) cardiac impairment was observed. The overall RR ranges from 70% to 76% showing the high and reproducible efficacy, furthermore, 18-35% of pts achieved a CR. On visceral sites, RR ranges from 56% to 86%. These results confirmed that NVB + fractionated doses of DX has major and reliable antitumour activity as front line therapy. Given its very favourable tolerance profile, low morbidity and absence of life threatening cardiac toxicity, out patient administration of NVB + DX (both 25 mg/m² on days 1 & 8) could be recommended as first line treatment for ABC.

P86 IDENTIFICATION AND CHARACTERISATION OF TWO GENES ASSOCIATED WITH ANDROGEN REGULATION IN THE HUMAN PROSTATE.

G.P. Collett*., A.M. Betts, D.E. Neal, and C.N. Robson, Department of Surgery, Newcastle University, Newcastle, NE2 4HH

Androgens are intimately associated with the growth and progression of prostate cancer. In an attempt to understand the mechanisms by which these tumour cells undergo the transition from an androgen dependent to an androgen independent state we have used the mRNA differential display technique to isolate androgen regulated genes from the prostatic epithelial cell line LNCaP and from primary cultured stromal cells obtained from patients undergoing transurethral resection of the prostate for bladder outflow obstruction. Northern analysis was used to confirm that these genes were truely androgen regulated. DNA sequence analysis of two cDNA clones revealed greater than 95 per cent homology to Peroxisome proliferator activated receptor-alpha (PPARa), a member of the steroid receptor superfamily of nuclear transcription factors and to Talin, a gene involved in cell motility and adhesion.

Both PPARa and Talin showed between 2-3 fold down-regulation in mRNA levels following chronic exposure to the synthetic androgen, Mibolerone (48-96 hours). Exposure of cells to the anti-androgen, Casodex prevented Mibolerone-induced down-regulation of these genes implying regulation is through the Androgen Receptor. Treatment of cells with the protein synthesis inhibitor cycloheximide prior to Mibolerone exposure blocked the down-regulation in mRNA suggesting that these genes are indirectly regulated by androgens.

Using RNA in situ hybridisation we have localised the PPARa and Talin transcripts within the prostate gland. Further data will be presented on the association between the expression of these genes and the stage or grade of prostate cancer.

P87 ROLE OF TRK-A EXPRESSION IN PPNET CELL LINES*

D.Li, S.Buckley, P.Berry, I.Lewis and S.A.Burchill, Cancer Research Unit, St James University Hospital, Leeds LS9 7TF.

Peripheral primitive neuroectodermal tumours (pPNETs) and neuroblastoma are paediatric malignancies derived from neural crest cells. We have previously shown nerve growth factor (NGF) induces differentiation in neuroblastoma cells[1]. This effect occurs following phosphorylation of Trk-A (the high affinity NGF receptor) which is expressed by neuroblastomas. In this study we have examined the effect of NGF and Trk-A expression in pPNET cell lines.

Two pPNET cell lines were used, RDES and TC-32. A neuroblastoma cell line, SK-N-SH, was used as a positive control. The effect of NGF (10-80ng/ml) on cell growth and differentiation was measured over 3 days. Cells were analysed by reverse transcriptase polymerase chain reaction (RT-PCR) for expression of the high affinity NGF receptor, Trk-A. TC-32 and RDES cells were transfected by lipofection with Trk-A DNA in the PLNCX vector (a kind gift from Dr D Kaplan, NCI). Transfected cells were selected with G418. Expression of Trk-A was confirmed at the RNA level by RT-PCR and at the protein level by Western blot analysis using a rabbit polyclonal Trk-A antibody. The effect of Trk-A expression on cell growth and proliferation was examined by incorporation of bromodeoxyuridine incorporation and cell number using a haemocytometer. Cell phenotype was examined by Western blot analysis and morphology. The effect of NGF in transfected and nontransfected cells was examined. NGF did not induce proliferation or differentiation in the pPNET cells. In SK-N-SH cells, NGF enhanced the neuronal phenotype demonstrated by increased neurofilament expression and neurite extension. Trk-A mRNA was detected by RT-PCR in the SK-N-SH cells, but not in the pPNET cells. Following transfection of RDES and TC-32 cell lines with Trk-A, a neuronal phenotype and neurite extension was induced under normal growth conditions. Transfection of cells with Trk-A had no effect on cell proliferation and cell number. Treatment of transfected cells with NGF increased proliferation and cell number, but did not further increase neurite extension.

In summary, NGF did not induce differentiation of pPNET cell lines. This lack of effect may be due to absence of a functional NGF receptor. Transfection of pPNET cell lines with Trk-A induced a neuronal phenotype, which was associated with an increase in cell proliferation following treatment with NGF. These observations suggest absence of Trk-A receptors in pPNETs may contribute to their undifferentiated phenotype and may provide a mechanism for the modulation of pPNET cell behaviour.

1 Burchill SA, Berry PA and Lewis IJ.(1995). *J. Neurol. Sci.*, 133:3-10.

P88 **THE REGULATORY ACTION OF CYTOKINES ON TUMOUR PROLIFERATION**

**J. Lawry*, A. Andalib, M.O.Smith, J.A.Royds[1], Institute for Cancer Studies and Department of Pathology[1], University Medical School, Beech Hill Road, Sheffield. S10 2RX (J.Lawry@sheffield.ac.uk)**

Whilst the growth stimulatory or inhibitory actions of cytokines have been reported for many cell types, the analysis of their mode of action is less well known. We have previously reported on the role of TGF$\beta_1$ in controlling proliferation in colon cancer cell lines, through the actions of the cyclin dependent kinase inhibitors (CDKIs) p15$^{(INK4B)}$, p21$^{(Cip1/WAF1)}$ and p27$^{(Kip1)}$. As colon tumours progress, cells become unresponsive to TGF$\beta_1$ which has been linked with recurrence and metastases. *In-vitro* proliferation studies were undertaken using flow cytometry and identified TGF$\beta_1$ as being growth inhibitory for HT29 cells, inducing p27, reducing p15 & p21expression; but growth stimulatory for SW742 cells (low level p27, induced p15 & p21), in serum free conditions. As this may be tissue biased, we have extend this study to analyse the modulation of cyclin expression by TGF$\beta_2$, IL-6, TNF$\alpha$ & IFN$\alpha$, on two melanoma cell lines derived from primary (WM793) and metastatic (WM1205) lesions of the same patient (M. Herlyn, Wistar Institute, USA).

WM793/1205 were both growth inhibited by TNF$\alpha$; stimulated by IFN$\alpha$; unresponsive to IL-6; whilst TGF$\beta_2$ stimulated growth of the metastatic line (43% increase in cyclin D expression), yet inhibited growth of the primary line (cyclin D reduced by 36%, cyclin B by 44%). Minimal changes were seen in cyclin A and E with any cell line or cytokine. In the parental line (WM793), TGF$\beta_2$ induced inhibition was also associated with a 200% increase in the expression of CDKI p15, 30% increase in p21, and a 30% decrease in p27 over controls, representing opposite results to those seen in the colon cell lines.

Funding was from the Yorkshire Cancer Research Campaign.

P89 PRODUCTION OF THE ANGIOGENIC FACTORS VEGF AND bFGF AT TUMOUR SITES MAY ALSO CONFER AN ACUTE HAEMODYNAMIC ADVANTAGE TO THE TUMOUR.

J.O. Curwen and D. Ogilvie, CME department, Zeneca Pharmaceuticals, Mereside, Alderley Park, Macclesfield, Cheshire SK10 4TG.

A wide range of human tumours express the growth factor VEGF. It has been postulated that both this growth factor and bFGF may play an important role in tumour progression by aiding angiogenesis within tumours. Recent literature reports have demonstrated that both VEGF and bFGF have haemodynamic effects when given systemically to rats and rabbits (Cuevas *et. al.*, Science 1991 254, 1208-1210 and Yang *et. al.*, J. Cardiovasc Pharmacol 1996 27, 838-844 ). In both cases the growth factors have been shown to induce significant hypotension without direct cardiac inhibition. In addition, VEGF has been shown to cause dilation of dog coronary arteries (Ku *et. al.*, Am J Physiol 1993 265, H586-H592). In the experiments described below we examined the simple haemodynamic effects of both human VEGF and human bFGF in an anaesthetised rat preparation.

Rats were anaesthetised and the carotid artery and the jugular vein were canulated to enable blood pressure and heart rate measurement and i.v. growth factor administration respectively. The preparation was allowed to equilibrate until a steady baseline blood pressure was obtained. Growth factors were then administered via the jugular vein cannula and their effect on blood pressure and heart rate recorded.

VEGF induced blood pressure falls of up to 82 mmHg at a maximal dose of 8 μg. bFGF induced smaller decreases in blood pressure with maximal falls of 36 mmHg at a maximal dose of 20 μg. A rapid desensitisation (tachyphylaxis) was observed following both VEGF and bFGF administration. It was found that, following a submaximal dose of VEGF a second identical dose of VEGF was ineffective but a submaximal dose of bFGF induced an additional blood pressure fall. Conversely, following a submaximal dose of bFGF, a second identical bFGF dose was ineffective but a submaximal dose of VEGF induced an additional blood pressure fall. This indicated that bFGF and VEGF induced blood pressure changes via different pathways. Given that VEGF and bFGF have their own receptors it seemed likely that the hypotensive effect was mediated by the individual growth factor receptor.

In conclusion, VEGF and bFGF both induced hypotension associated with a reflex tacchycardia by independent pathways. Local dilatation of the blood supply near a tumour induced by either VEGF or bFGF could result in an increased blood supply to the tumour by the process of "vascular steal". Thus bFGF and VEGF may act to promote tumour progression by acute haemodynamic mechanisms in addition to their angiogenic properties.

P90

**Effects of Enhanced Platelet Derived-Endothelial Cell Growth Factor/ Thymidine Phosphorylase Activity on Tumour Growth and Metastasis**

Leigh Griffiths*, Roy Bicknell+ and Ian J. Stratford*

* Experimental Oncology, Department of Pharmacy, Manchester University, Oxford Road, Manchester, M13 9PL.
+ Molecular Angiogenesis Group, ICRF, Institute of Molecular Medicine, University of Oxford, John Radcliffe Hospital, Oxford, OX3 9DU

The vascularisation of tumours is relatively poor and disordered compared with that of normal tissue, leading to insufficient oxygenation and delivery of nutrients to some tumour cells. Platelet derived-endothelial cell growth factor (PD-ECGF) (also known as thymidine phosphorylase) is a 55kDa polypeptide existing *in vivo* as a 110kDa homodimer. PD-ECGF, which was originally isolated from platelets as an endothelial mitogen and has chemotactic activity *in vitro* and angiogenic activity *in vivo*. It has been shown that thymidine phosphorylase activity is critical for the angiogenic activity of PD-ECGF. Overexpression of PD-ECGF/TP in MCF-7 breast carcinoma cells has produced markedly enhanced tumour growth. Thus the presence of PD-ECGF/TP may affect tumour oxygenation which has implications for both radiotherapy of tumours and use of bioreductive drugs. Expression of PD-ECGF/TP protein has been investigated as a prognostic indicator in breast, ovarian, bladder and colorectal tumours, and in some instances has been associated with invasiveness and malignancy of tumours.

We are investigating the effects of modest overexpression of TP on tumour growth, vascular density, hypoxic fraction and metastasis. Results indicate that tumours grown from the human breast cancer cell line MDA 231 (with a physiologically significant expression 6 fold higher than basal levels) develop significantly quicker than their wild type counterparts. However these cells do not grow more rapidly *in vitro*. Tumours with enhanced TP also show differences in their vasculature and hypoxic fraction. Currently we are studying the metastatic characteristics of these cells.

P91 ANTIVASCULAR EFFECTS OF CHEMOTHERAPEUTIC AGENTS

GG Dark*, SA Hill, DJ Chaplin. Tumour Microcirculation Group, Gray Laboratory, Northwood, Middlesex, HA6 2JR, UK.

Anticancer agents damage both malignant and non-malignant tissues and a major pharmacological goal remains the development of highly selective non-toxic therapies for the treatment of malignancies. Many antineoplastic agents act on rapidly proliferating cells, without regard for the malignant phenotype. Damage to the endothelium may be responsible for unwanted toxicity manifested by non-malignant tissues, however, tumour associated endothelium represents a key target for the development of new approaches to cancer therapy.

Using a tumour microenvironment model system we have examined the cytotoxic effects of a number of chemotherapeutic agents. Many agents do not have an effect on endothelial cells, but the tubulin polymerisation and depolymerisation inhibitors show a preferential action against endothelium, compared to cancer cells. Podophyllotoxin, although a weak tubulin-binding agent, is more toxic to tumour cells than endothelium. The derivative Etoposide shares this property, without any tubulin-binding action. Colchicine, Dolastatin, Taxotere, Vinblastine, Vincristine and Combretastatin all have increased toxicity towards endothelium, with enhancement of this action under hypoxic (1% $O_2$) conditions. Tumour cells show a reverse relationship with increased resistance to these agents under hypoxia. The presence of tumour cells in coculture with the endothelium causes further enhancement of the cytotoxicity. This is particularly true for Taxotere and Vincristine, at 1% $O_2$, and there is a small enhancement with Combretastatin A-4 and its more soluble prodrug. The tubulin-binding agents appear to have most of their effect on proliferating endothelium, and this may explain the enhancement seen with endothelial:tumour coculture. The breast cancer cell lines studied, produce VEGF in significant quantities, and cause a higher endothelial proliferation rate. In contrast, quiescent endothelium is more resistant to these agents, particularly combretastatin A-4 prodrug, which appears to have no detectable toxicity.

In view of these selective actions against tumour associated endothelium, vascular studies were performed in experimental and human breast cancer models *in vivo*. Histology of a variety of human xenografts and murine transplants indicated extensive haemorrhagic necrosis with the tubulin binding agents Colchicine, Vinblastine and Vincristine, although only when administered at the maximum tolerated dose (MTD). Taxotere does not share this effect. Combretastatin A-4 prodrug is most promising as it causes selective vascular shutdown in tumour vasculature at doses <10% MTD and without evidence of morbidity or action to non-tumour tissues. Thus the antivascular effects of these agents could be used for therapeutic gain.

P92

**Analysis of integrin expression on six melanocytic cell lines derived from C57/Bl mice.**

D.R.Rutherford, D.Davies, I.R.Hart and J.F.Marshall
Richard Dimbleby/ICRF Department of Cancer Research, St. Thomas Hospital, London SE1 7EH, UK.

As a prelude to investigating their in vivo behaviour we have examined the integrin repertoire of 2 melanoma lines (B16 F1 and B16 F10), 2 melanocyte strains (Melan-a and Mel-ab) and two H-Ras-transformed melanocyte lines (Ltr Ras2 and Ltr Ras3) all of which are syngeneic for C57/Bl mice. Using monoclonal antibodies specific for the mouse integrin subunits α1, α2, α4, α5, α6, αv, β1 and β3, flowcytometry revealed that the most abundant subunits were αv, β1 and α6 whereas α4 was expressed only by the B16 lines. Immunoprecipitation showed that the α4 subunit exists both as the 180 kDa form and as a doublet of approximately 85/70 kDa. In contrast, the 150kDa and 80/70kDa forms, but not the 180kDa form, were detected in human melanoma cell lines. Immunoprecipitation with mAb C8F12 (anti-αv) revealed a 150 kDa αv subunit associated with 3 subunits of 120, 95 and 90 kDa. Co-migration of the 120 kDa band with the β1 subunit suggested this band was β1. Immunoprecipitation of β3 integrins with mAb 2C9 suggested the 90 kDa band associated with αv probably was β3. Thus all 6 lines appeared to express at least three different αv integrins: αvβ1, αvβ3 and $\alpha v\beta_{90kDa}$ (possibly αvβ5). Since C57/Bl-derived melanocytic lines express many of the same integrins as those expressed by human melanoma cell lines, this syngeneic model may be useful for studying the role of integrins in metastatic melanoma.

P93 **IN VITRO EFFECTS OF TWO NOVEL CYTOCHALASINS ON TUMOUR AND ENDOTHELIAL CELLS**

I.G.Popa*, K.Grosios[1], M.J.Thompson[2], D.J.Maitland, M.C.Bibby[1], J.A.Double[1]
Chemistry and Chemical Technology, Clinical Oncology Unit[1], Biomedical Sciences[2], University of Bradford, Bradford, UK.

Cytochalasins are a class of more than 50 structurally related secondary fungal metabolites, which have been shown to have marked effects on the morphology and motility of cells and have cytostatic, as well as cytotoxic activity. The reported effects and properties of these compounds on cells and tissues in culture vary vastly within the class. Their ability to inhibit cell motility could have important implications, considering that tumour and endothelial cell migration is a major aspect of tumour development and progression. The present study investigated the potential of these natural products as anti-cancer agents.
Two novel cytochalasins $S_{Hyp}$ and $T_{Hyp}$ were isolated from the fungus *Hypoxylon terricola* and their respective structures determined using various spectroscopic techniques. They have been tested for their cytotoxic activity on four cell lines and have been found to have differential effects after continuous 96 h exposures, varying from 0.02 mM ($IC_{50}$ value for $S_{Hyp}$ on MT1, a breast cancer cell line) to 3 mM ($IC_{50}$ value for $T_{Hyp}$ on MAC15A, an adenocarcinoma of the colon cell line). These cytotoxic effects are much lower than the cytotoxicities of other members of the class previously tested in our laboratory.
The importance of endothelial cells in tumour angiogenesis and tumour development is widely recognised. and therefore our finding that primary human umbilical vein endothelial cells (HUVEC) are more sensitive to these agents than tumour cell lines, promoted their further investigation.
Staining for F-actin, using rhodamine conjugated phalloidin, revealed that exposure of HUVEC and HMEC-1 (a human microvascular endothelial cell line) to non-cytotoxic concentrations of both cytochalasins caused extensive F-actin disorganisation. Additionally, this effect was much stronger than that exhibited by other members of the class.
The above results indicate that these natural products have potential antiangiogenic activity and therefore further evaluation is worthwhile.
This work has been supported by War on Cancer, Bradford, UK.

P94 **ELEVATED EXPRESSION OF VASCULAR ENDOTHELIAL GROWTH FACTOR IN METASTATIC HUMAN COLORECTAL CARCINOMA CELL LINES AND IN SERA FROM PATIENTS WITH ADVANCED COLORECTAL CANCER.**

D.F.Baban[1], N.R.J. Cruickshank[2], A. Fabra[3], B. Liu[1] D.J. Kerr[1] and L.W. Seymour[1].
[1]CRC Institute for Cancer Studies, University of Birmingham, Birmingham B15 2TJ, U.K., [2]Dept. of Surgery, Queen Elizabeth Hospital, B15 2TH,, U.K. & [3]Department of Metastasis, Hospital Duran y Reynals, Barcelona 08907, Spain.

Vascular endothelial growth factor (VEGF) plays an important role in tumour growth and differentiation. VEGF is expressed by numerous human tumour cells *in vitro* and *in vivo* In contrast VEGF expression in normal tissues is limited and found only in activated macrophages and keratinocytes during wound healing, ovarian corpus luteum formation and endometrium regeneration during the female menstrual cycle, renal glomerular visceral epithelium and mesangial cells. VEGF is a paracrine-acting growth factor which exert its several effects by binding tyrosine kinase-linked receptors (flt-1 & KDR) on vascular endothelial cells. It has been suggested that VEGF expression may play an important role in regulation of tumour angiogenesis and also to mediate the enhanced permeability of tumour vasculature important for protein extravasation and stroma formation. Recently there have been suggestions that VEGF may also play a role in metastasis. It has been shown (Claffey et.al., 1996, Cancer Res.56: 172) that the human melanoma cell SK-MEL-2 stably transfected with full-length mouse VEGF-164 cDNA expressed and secreted large amounts of mouse VEGF and formed well-vascularized tumours with approx. 50-fold higher experimental metastasis in SCID mice compared to parental cells.

In this study we have examined the possible link between VEGF expression and metastasis in colorectal cancer. In preclinical studies we have characterised VEGF expression in human colorectal cell lines HT29 & KM12, comparing parental cells and their metastatic variants *in vitro* and *in vivo*. These cells were injected into nu/nu mice either orthotopically or ectopically (s.c.) and VEGF expression was studied by RT-PCR and northern blotting in cell lines, in primary tumours and spontaneous metastases. Our preliminary results suggest that VEGF expression is higher (60-550%) in metastatic variants of cell lines under-study than in corresponding parental cells. Furthermore, greater VEGF expression was observed when cells were injected into the orthototopic site from which spontaneous metastatic lesions are formed than into the ectopic site. In clinical studies we have used ELISA to measure VEGF levels in pre-operative serum from patients with varying stages of colorectal cancer. There was a significant increase in VEGF level (pg/ml) in serum of patient with Duke stage D (905 ± 117) tumours compared with stage A (282 ± 60) patients.and age-matched normals (233 ± 29). We aim to further elucidate the role of VEGF in tumour biology and progression, and to establish whether the simple and relatively non-invasive procedure of measuring serum levels of VEGF can have significance as a diagnostic test..

P95 **PLASMINOGEN ACTIVATOR INHIBITOR TYPE 1 (PAI-1) OVER-EXPRESSION IN HUMAN FIBROSARCOMA CELLS FACILITATES EXPERIMENTAL METASTASES IN ATHYMIC MICE**, V.A. Carroll*[1], P. Hufnagl[1], E. Matsuda[2] and B.R. Binder[1], [1]Dept. of Vasc. Biol. and Thromb. Res., Vienna Univ., Austria and [2]Dept. of Orthopaedic Surgery, Kanazawa Univ., Japan.

Plasminogen activator inhibitor type 1 (PAI-1) has been implicated in the metastatic process in a number of human cancers including breast, skin, colon, lung and brain. In an experimental metastasis model in athymic mice we have shown that the ability of HT1080 human fibrosarcoma cells to colonise the lung after intravenous injection into the lateral tail vein increased after consecutive *in vivo* passages and that this increased metastatic potential correlated positively with their PAI-1 and tissue factor (TF) expression, (Tsuchiya H., *et al. Fibrinolysis* 1992; 6(2):60). Additionally, two cell lines were established from the parent HT1080 with either a low endogenous expression of PAI-1 and TF (1-3C) that exhibited a low metastatic potential, and a second cell line with a high endogenous expression of PAI-1 and TF (26-6) that exhibited a high colonisation potential (Matsuda E., *et al. Thromb. Haemost.* 1993;69(6):548). Furthermore, the number of pulmonary metastases formed after injection with 26-6 cells were significantly decreased after pretreatment of mice with either heparin to inhibit TF activity or with an inhibitory antibody against PAI-1 (Matsuda E., *et al. Fibrinolysis* 1994;8(1):7).

In order to further elucidate the role of PAI-1 and TF in this model, the low metastatic cell line (1-3C) was transfected with human PAI-1 cDNA containing the entire coding region (1430bp) inserted into a plasmid (BCMGSNeo). A stable transfected clone was isolated (3F52) that exhibited a twenty fold increase in PAI-1 antigen concentration (28.9±8.4μg/$10^6$cells/24hours) as compared to mock transfected (1-3CM) cells (1.2±0.5μg/$10^6$cells/24hours). TF activity between these two cell lines remained virtually unchanged.

When the ability of these cells to form metastatic colonies in the lung was evaluated, it was observed that 3F52 exhibited a significantly increased metastatic potential as determined by the mean number±SD of colonies on the lung surface, 276±251; range (120-650) as compared to the control (1-3CM), 0.5±0.6; range (0-1); $p<0.05$.

We therefore provide evidence that PAI-1 antigen overexpression facilitates pulmonary metastasis formation in this mouse model. Lack of dissolution of fibrin due to local inhibition of the fibrinolytic system may favour the formation and/or growth of metastases in this system.

P96 **UTILISATION OF VARIOUS MODEL SYSTEMS TO EXAMINE TUMOUR CELL-DERIVED FACTOR STIMULATION OF FIBROBLAST GLYCOSAMINOGLYCAN SYNTHESIS**

M. Edward, J.L. Godden and R.M. MacKie, University Department of Dermatology, The Robertson Building, Glasgow G12 8QQ.

We have previously shown that melanoma cell-conditioned medium (CM) contains potent fibroblast glycosaminoglycan (GAG) stimulating activities, and now present evidence that such tumour-derived factors are equally potent in stimulating GAG synthesis by fibroblasts within contracted collagen lattices. Such interactions may be important in facilitating tumour growth and invasion as hyaluronan is associated with cell proliferation and migration, and found in enhanced amounts surrounding many tumours. We have also examined the ability of melanoma cells to release GAG-stimulating activities when grown as spheroids, and the effect of cocultures of fibroblasts and melanoma cells on GAG synthesis.
Human breast skin fibroblasts were seeded into collagen gels which were allowed to contract for 14 days before a 24 hr exposure to serum-free human melanoma cell CM containing $^3$H-glucosamine. Radiolabelled GAGs were isolated from both the medium fraction and the contracted collagen lattices (pretreated with bacterial collagenase), by cetylpyridinium chloride precipitation, the precipitate dissolved in 2M NaCl, and samples taken for scintillation counting. Spheroid cultures were prepared by incubating melanoma cells on agar-coated plates, the spheroids washed with serum-free medium, and CM prepared under serum-free conditions on an agar substrate. The spheroid CM was then added to fibroblast monolayers and incubated in the presence of $^3$H-glucosamine for 24hr. C8161, Hs294T and A375 melanoma cell CM from monolayer cultures stimulated a 5.0, 5.1 and 3.7 fold increase respectively in incorporation of $^3$H-glucosamine into medium GAGs, and a 4.1, 5.1 and 4.9 fold increase respectively into collagen lattice-associated GAGs. Conditioned medium from spheroid cultures appeared to lack GAG-stimulating activity, however, preincubation of CM from monolayer cultures of tumour cells on agar coated dishes resulted in loss of activity suggesting that the GAG-stimulating activity is binding to the agar substrate. Spheroid CM is currently being utilised using non-agar substrates.
We have already shown that there are a number of factors produced by melanoma cells that act synergistically in stimulating fibroblast GAG synthesis, and have now demonstrated that such factors also stimulate GAG synthesis by fibroblasts in a contracted collagen lattice model in which fibroblasts are normally poorly responsive to various growth factors. These preliminary results suggest the large increase in fibroblast GAG stimulation brought about by melanoma cell-derived factors in monolayer cultures is also applicable to more biologically relevant models substantiating the importance of this paracrine relationship.

P97

PROGNOSTIC EVALUATION OF STROMELYSIN 3 EXPRESSION IN HUMAN BREAST CANCER.

A.Ahmad[1], E.A.Dublin[2], D.M.Barnes[2], A.Hanby[2], P.Basset[3] and I.R. Hart[1].

[1]Richard Dimbleby/ICRF Department of Cancer Research, St. Thomas Hospital, London SE1 7EH, UK.[2]ICRF Clinical Oncology Unit, Guys Hospital, London, SE9 7RT, UK.[3]IGBMC, CNRS/INSERM/ULP, Strasbourg, France.

Stromelysin 3 (ST3) is a matrix metalloproteinase which is expressed specifically in stromal fibroblasts surrounding invasive foci in human breast cancers (Basset *et al*, Nature 348:699-704,1990). In particular, ST3 RNA has been detected in most invasive breast carcinomas and in situ carcinomas of the comedo type. Moreover, high levels of ST3 RNA in primary tumours have been reported to predict greater likelihood of recurrent disease (Engel *et al*, Int. J. Cancer,58,1-7, 1994). In order to study the possibility that ST3 expression serves as a prognostic marker in breast cancer patients we have used a monoclonal antibody (5ST-4A9) raised against the C-terminal portion of the hemopexin-like domain of the ST3 molecule. Using MAb 5ST-4A9, immunohistochemical analysis on formalin-fixed, paraffin-embedded material was performed utilising heat-mediated antigen retrieval and a peroxidase-conjugated streptavidin biotin system. 30 samples from each of Grade I, II and III invasive breast carcinoma, 30 cases of lobular carcinoma, 30 cases of ductal carcinoma-in-situ and 20 cases of benign breast disease have been immunostained. Preliminary analysis of the data indicates that: a) there is no ST3 expression in benign breast tissue (20 out of 20 negative) b) increasing ST3 expression is associated with higher grade tumours (26/30 strongly positive grade III versus 5/30 grade I strongly positive) and c) it appears that high ST3 levels are associated with increased risk of recurrence. These results support the data of Engel *et al* and show that differences in mRNA are carried through to the protein level in breast cancer progression.

P99

PHASE II STUDY OF MITOMYCIN C AND ORAL ETOPOSIDE FOR ADVANCED GASTRO-OESOPHAGEAL CARCINOMA. J.P. Braybrooke*, K.J. O'Byrne, D.J. Propper, M.P.Saunders, A.J. Salisbury, P. Boardman, M. Taylor, T. S. Ganesan, D. C. Talbot, A. L. Harris. ICRF Medical Oncology Unit, Churchill Hospital, Oxford OX3 7LJ, U.K.

Etoposide is a topoisomerase II inhibitor with phase specific and schedule dependent activity. Previous studies have shown that oral etoposide is an effective treatment in small cell lung cancer and ovarian cancer. This study evaluated protracted oral etoposide in combination with mitomycin C for the treatment of inoperable gastro-oesophageal carcinoma.

28 consecutive patients (22 male, 6 female; age range 36 - 86 years, median 59 years) with advanced histologically proven adenocarcinoma of the upper gastrointestinal tract (12 gastric, 6 gastro-oesophageal, 9 oesophageal) were treated with intravenous bolus mitomycin C 6mg/m$^2$ every 21 days to a maximum of 4 courses. Oral etoposide capsules 50 mg b.i.d.(or 35mg b.i.d. liquid) were administered days 1 to 10, extending to 14 days in subsequent courses if no haematological toxicity, to a maximum of 6 courses.

A total of 107 courses of chemotherapy were administered. 26 patients were evaluable for response (12 measurable, 14 evaluable). 4 patients had a documented radiological response (1 complete response, 3 partial responses) with an objective response rate of 15% (95% confidence interval 4 - 35%). 8 patients had stable disease and 14 progressive disease. The median survival in all patients was 6 months extended to 9.5 months in those patients with stable disease or documented response.. The schedule was well tolerated with no treatment related deaths. 9 patients experienced leucopenia (7 grade II, 2 grade III). Nausea and vomiting, alopecia and fatigue were the predominant toxicities. This out-patient schedule is well tolerated and shows modest activity in the treatment of advanced upper gastrointestinal adenocarcinoma.

P98

INFUSIONAL ETOPOSIDE PHOSPHATE IN RELAPSED OVARIAN CANCER. K.J. O'Byrne*, S. Joel[1], D. Propper, J. Braybrooke, A. Sanders, M. Elliott, M.Slevin[1] and T.S. Ganesan. ICRF Medical Oncology Unit, Churchill Hospital, Oxford, OX3 7LJ, U.K.; [1]Dept. of Medical Oncology, St Bartholomew's Hospital, London, EC1A 7BE, U.K.

Oral etoposide is effective in the treatment of relapsed, cisplatin resistant, ovarian cancer (response rate 21-24%). The absorption of oral etoposide is incomplete and variable, both between and within patients. We have shown that therapeutic monitoring of infusional etoposide is feasible to maintain a desired plasma concentration. Etoposide phosphate (EP), a more water soluble pro-drug of etoposide permits easier continuous intravenous infusion (inf) in outpatients. We have therefore conducted a study evaluating the administration of EP as an inf over 120 hours every three weeks, maintaining the plasma etoposide concentration ([E]) at 2µg/ml in patients with recurrent or refractory ovarian cancer. A loading EP dose of 20mg/m$^2$, given over 30 minutes, was immediately followed by the inf at a starting dose of 2mg/m$^2$/hour. [E] was monitored approximately 18 and 66 hours into the infusion and the rate modified to ensure patients were treated at the planned target level 2µg/ml [E]. Evaluation of response was performed at the end of 3 cycles and patients received a further 3 if there was no progressive disease. 16 patients (age 42-79, median 64 years) with resistant (9) or recurrent (7) disease, having been previously treated with platinum based chemotherapy (16) and taxol (2) were entered into the trial. 66 cycles (median 4 cycles) of EP were administered with 7/16 patients receiving six cycles. Mean [E] in 15 evaluable patients at 18 hours into cycle 1 was 2.89±0.78 µg/ml, and was within 20% of target in only 3 patients. At 66 hours of cycle 1, after dose modification, mean [E] was 2.16±0.33 µg/ml, with 10/14 patients within 20% of target. Etoposide plasma clearance significantly correlated with EDTA clearance (r=0.64, p=0.015) such that cycle 1 [E] at 18 hours in patients with EDTA clearance < 55mls/minute was 3.54 µg/ml, compared to 2.38 µg/ml in patients with clearance >55mls/minute. Alopecia (100%) was the main toxicity and 4/16 patients developed Hickman line related deep venous thrombosis. 15/16 patients had neutropenia grade 2 or less. One patient died during the first cycle of treatment from a disease related bowel perforation with grade 4 neutropenia. 14/16 patients are evaluable for response; the disease was stable in 4/16 and progressive in the remaining 10. These results demonstrate the feasibility of pharmacokinetically guided EP infusions to maintain [E] thereby reducing interpatient variability. A further fourteen patients are being treated to maintain [E] at 3 µg/ml.

WITHDRAWN

P101 LEVELS OF TAMOXIFEN AND TWO MAJOR METABOLITES IN TAMOXIFEN-TREATED BREAST CANCER PATIENTS AND CORRELATIONS WITH RESPONSE.

J.MacCallum, J.Cummings, J.M.Dixon* and W.R.Miller, ICRF Medical Oncology Unit and *Edinburgh Breast Unit, Western General Hospital, Edinburgh, EH4 2XU.

Patients treated with tamoxifen for primary breast cancer often manifest de novo or acquired resistance, which may be the result of tamoxifen metabolism. We have developed a solid-phase extraction method and reversed-phase HPLC separation to determine levels of tamoxifen (TAM) and metabolites 4-hydroxytamoxifen (4OH) and desmethyltamoxifen (DMT). Portions of tumour (50mg) were taken at surgery from 45 patients with oestrogen-receptor positive tumours, after between 3-90 months of treatment. During this time, response (both after 3 months treatment and at definitive surgery) was monitored by regular ultrasound and clinical measurement of the tumour. Patients were classified as responding where tumour volume decreased by at least 20% between initial biopsy and these chosen timepoints; non-responders showed either no change or an increase in tumour volume. Patients were then categorised according to the response of their tumour throughout treatment; tumours decreasing in size, with a continued reduction or no change in tumour size thereafter (R-R, n=20); tumours initially reducing in size on therapy, then increasing in size at some point thereafter (R-NR, n=21); tumours remaining the same size or increasing in size on treatment (NR-NR, n=4). Levels of TAM, DMT and 4OH for the three categories of patients are shown in the table below as ng/g of tissue ± S.D..

| | R-R | R-NR | NR-NR |
|---|---|---|---|
| TAM | 378.84±283.65 | 387.98±330.49 | 296.47±124.45 |
| DMT | 746.71±1123.93 | 599.6±579.3 | 393.42±232.12 |
| 4OH | 142.67±110.89 | 82.69±148.36 | 145.05±98.7 |

Although mean levels between tumours vary greatly, these results provide no evidence that resistance to tamoxifen treatment is caused by reduced levels or metabolism of TAM.

P102 3-METHYLADENINE EXCRETION IN PATIENTS WITH MALIGNANT MELANOMA RECEIVING DACARBAZINE.

D.E.G. Shuker*[1], J. Braybrooke[2], J.E. Crawley[1], E. Flanagan[2] and A.L. Harris[2]. [1]MRC Toxicology Unit, University of Leicester, Leicester LE1 9HN, [2]ICRF Medical Oncology Unit, The Churchill, Oxford Radcliffe Hospital, Oxford OX3 7LJ.

Dacarbazine is converted to a methylating agent after oxidative N-demethylation. A number of studies have shown that $O^6$- and N7-methylguanine are increased in peripheral lymphocyte DNA of patients treated with dacarbazine and other methylating agents. We have recently shown that urinary 3-methyladenine (3MA) is increased in patients treated with methylating agents and that there is substantial interindividual variation in the response (Prevost *et al., Biomarkers,* 1996. **1**, 244-251). As part of a broader study of pharmacodynamic monitoring of dacarbazine therapy, 3MA excretion has been measured in patients with malignant melanoma who were treated with dacarbazine 1g/$m^2$ iv infusion over 1 hour every 3 weeks for a maximum of 6 cycles. Tamoxifen 20 mg o.d. was commenced 24 hours after the first infusion and continued throughout the study. Timed urine samples were collected from a series of patients (n=12) undergoing cycles 1 and 2 of treatment. A urine sample was collected immediately prior to drug administration and at timed intervals (0-4, 4-10, 10-20 and 20-24 hours) over the following 24 hours. Following collection the total urine volume was recorded and aliquots were stored at -70°C until analyses were carried out 3MA was determined in 2 mL aliquots from each sample by immunoaffinity-ELISA (Prevost *et al., Carcinogenesis,* 1990, **11**, 1747-1751). The total amount of 3MA excreted 24h. post administration varied about fivefold (249-1637 nmol/24h, n=12). In most patients for whom complete sets of data are available (n=9) the profiles of 3MA excretion showed either a broad peak of excretion over 24h (n = 5) or a sharper peak of excretion between 4 and 10h (n = 3). In one case a clear maximal excretion was not observed within 24h. These preliminary results suggest that there are differences in not only the extent of formation but also the kinetics of repair of 3MA between individuals receiving the same dose of dacarbazine. The differences in repair may be due to polymorphisms in 3MA glycosylase activity and this aspect is currently under investigation.

P103 TUMOUR CONTAMINATION OF PERIPHERAL BLOOD PROGENITOR CELL (PBPC) HARVESTS IN PATIENTS WITH HIGH RISK STAGE II AND IIIA BREAST CANCER.

A.C.Humphreys, K.MacRae, M.J.Lind, A.V.Boddy. Cancer Research Unit, University of Newcastle, Newcastle upon Tyne, NE2 4HH.

High dose chemotherapy (HDC) with PBPC support is being used increasingly in the treatment of solid tumours, particularly breast cancer. There is concern that circulating tumour cells may also be collected during PBPC harvest, reinfused into the patient and contribute to relapse.
Unfortunately there is no unique antigen which characterises breast tumour cells, and therefore epithelial cell antigens have been used as targets for identification of tumour cells. Cytokeratins (CK), which form part of the cytoskeleton of epithelial cells, are not present in haemopoietic cells. They have therefore been used to detect breast tumour cells in bone marrow, PBPC and peripheral blood. Using reverse transcriptase polymerase chain reaction (Rt-PCR), CK19 has been the most promising target, although it is not specific in all hands and has a false positive rate of 0 - 38%.
We have used the tumour cell line MDA-MB-231 to develop immunocytochemistry (ICC) and Rt-PCR methods to apply to clinical samples. Rt-PCR of CK19 can detect as low as 1 tumour cell in $10^6$ mononuclear cells (MNC). ICC using a pancytokeratin antibody (EPIMET kit) at the present time detects 1 tumour cell in $10^4$ MNC consistently. Work continues on this to improve sensitivity.
We have treated 14 patients with HDC and PBPC reinfusion as part of the Anglo-Celtic trial, which compares conventional and HD chemotherapy in patients with stage II and IIIa breast cancer with 4 or more positive axillary lymph nodes. Aliquots of PBPC were cryopreserved and analysed by Rt-PCR and ICC.
Mononuclear cells from 25 normal female volunteers without cancer and PBPC from patients with chronic myeloid leukaemia were used as negative controls for Rt-PCR.
The sensitivity and specificity of detection of tumour cells by the two methods will be compared, and the prevalence of tumour cell contamination in this group of patients presented.

P104 ISOLATION AND CHARACTERIZATION OF A LIPID MOBILIZING FACTOR FROM THE URINE OF CACHECTIC CANCER PATIENTS.

K Hirai*[1], O Ishiko[1], P T Todorov[2], M J Tisdale[2], [1]Dept of Ob&Gyn, Osaka City University, Japan 545, [2]Pharm Sci Inst, Aston University, Birmingham B4 7ET

Loss of body fat is common in many types of cancer and may be driven by catabolic factors produced by certain tumours. A lipid mobilizing factor (LMF) has been detected in the urine of patients with cancer cachexia by monitoring the ability of stimulating lipolysis in freshly isolated murine epididymal adipocytes. The material was found not to be present in the urine of patients without cachexia. The LMF has now been isolated from urine using a combination of ion-exchange and hydrophobic chromatography and shown to stimulate glycerol release in a dose-dependent manner. Using plasma membranes from epididymal adipocytes the LMF caused stimulation of adenylate cyclase in a GTP-dependent process with maximal stimulation occurring at 0.1$\mu$M GTP. The material thus behaves like the natural lipolytic hormones, but differs in both charge (acidic) and molecular weight (40kDa). In addition the capacity to induce lipolysis and to stimulate adenylate cyclase were inhibited by the selective $\beta_3$-adrenergic receptor antagonist (SR59230A) at a concentration of $10^{-5}$M. The LMF was also capable of stimulation of adenylate cyclase in murine hepatocyte plasma membranes suggesting that other effects of cachexia (decrease in liver glycogen) may also be mediated by this factor. These results suggest that the LMF may be responsible for the systemic effects of cancer cachexia

### P105 MR SPECTROSCOPY IN PATIENTS RECEIVING CHEMOTHERAPY FOR BREAST CANCER.

M.W.Verrill*[2], D.J.Collins[1], J.Glaholm[1], G.S.Payne[1], M.O.Leach[1].

Institute of Cancer Research[1]& Royal Marsden NHS Trust,[2]Sutton, UK.

Primary medical therapy is an increasingly popular treatment option in patients with large operable breast cancers as it may downsize the tumour enabling breast conserving surgery, preferred by many patients to mastectomy. Although the response rates to pre-operative chemotherapy are high[1], not every patient benefits, and it is important to identify those with non responding tumours to enable early referral for surgery. Difficulties in clinical evaluation of these patients arise from inter-observer differences in tumour measurements and changes in tumour consistency which may impair tumour edge definition. To overcome these problems, it would be useful to have objective methods of assessment which are independent of volume.

From a group of patients included in trials of primary medical therapy[2,3], 14 were entered into a study of sequential magnetic resonance spectroscopy (MRS) tumour assessments. MRS was performed before and during treatment and the MRS changes were compared with volume response to chemotherapy, where possible measured by MRI. Using the Spearman rank correlation test of association, absolute phosphomonoester (PME) and total phosphate (TP) levels at week 3 were statistically significantly associated with volume response after 3 weeks (p=0.001, p=0.05 respectively) as were early changes in PME and TP (p=0.008, p=0.03 respectively) . In addition, in a number of cases, marked changes in MRS parameters occurred before corresponding changes in volume.

Although volume remains standard for response assessment in these patients, MRS examinations early in treatment may provide an objective measurement in those patients who are difficult to assess clinically. In the future we hope to feed information back into the clinic which may help to predict the eventual outcome of chemotherapy.

1. Smith IE, Walsh G, Jones A, et al. 1995: J Clin Oncol 13,424
2. Smith IE, Jones A, O'Brien M, et al. 1993: Eur J Cancer 29A,1796
3. Powles TJ, Hickish T, Makris A, et al. 1995: J Clin Oncol 13, 547

### P106 ELEVATED BLOOD MONOCYTE TISSUE FACTOR LEVELS IN PATIENTS WITH CANCER.

BA Lwaleed*[1], M Chisholm[1] and JL Francis[2]. [1]Dept. of Haematology, University, Southampton, U.K. [2]Hemostasis and Thrombosis Research Unit, FL 32701, U.S.A.

**The association between cancer and thromboembolic disease has been known since 1865[1]. The phenomenon is poorly understood, but continuous expression of tissue factor (TF), the principle initiator of blood coagulation[2] by endothelial cells, monocytes (mTF)/macrophages, may be implicated. Measurements of mTF may provide a good index of intra-vascular clotting activation and disease activity. Using a two stage kinetic chromogenic assay established in this laboratory[3], mTF levels were measured in controls [normal (n=60) and patients undergoing hernia/cholecystectomy (n=60)] and patients with benign and malignant disease of the bladder (n=73), prostate (n=81), breast (n=83) and large bowel (n=62), under resting conditions and after incubation for 6 hours with and without *E. Coli* endotoxin. Each benign disease group was divided into inflammatory and non-inflammatory categories. Cells were isolated in a one step procedure using leuko prep tubes with negligible effect on the mTF expression[3]. The control groups and the benign non-inflammatory groups gave similar results and have therefore been unified for further analysis. There was a significant difference between the malignant groups and the control groups for resting (P<0<001), un-stimulated (P<0.05) and stimulated cells (P<0.001). Similarly, the benign inflammatory groups differ from the control groups for resting (P<0.001), un-stimulated (P<0.05) and stimulated (P<0.001). There was no difference between the malignant groups and the benign inflammatory groups. Patients with malignant diseases gave results above the upper quartile of the normal control for resting (46%), un-stimulated (26%) and stimulated cells (62.4%). In conclusion, mTF level is raised in malignant conditions compared to controls and patients with non-inflammatory conditions but not patients with inflammatory conditions. Stimulated cells give better discrimination between the groups and may be of value in identifying high risk individuals.**

**References:**

1) Trousseau A. *Balliere*, 1865.
2) Nemerson Y. *Semin Haemost*. 1992; 29: 170.
3) Carvalho MG, Francis JL. *Thromb Haemostas*. 1993; 69: 601.

### P107 PHASE I TRIAL OF ORAL VINORELBINE (VRL) IN PATIENTS (PTS) WITH ADVANCED BREAST CANCER (ABC).

B.Chevallier[1†], J.Bonneterre[2], F. Le Bras[3], C.Focan[4], L.Mauriac[5], M.Piccart[6], P. Danel[3], E.Favreau[3], I.Sénac[3]. 1.CAC H. Becquerel (Fr.); 2. CAC O. Lambret (France); 3. I.R.P.F.(Fr.); 4. Hôp. St J. Espérance (Belg.); 5. Fond. Bergonié (Fr.); 6. Inst. J. Bordet (Belg.).

A new formulation of oral VRL is being developed, which should offer several advantages from the patients' perspective over the intravenous (I.V.) administration, particularly in the setting of chronic or palliative treatment. The aims of this Phase I were to determine the maximum tolerated dose (MTD) of oral VRL administered weekly, defined as more than 50 % incidence of grade (G) 4 haematological or G 3/4 non haematological toxicity, to define a recommended dose (RD) for further trials and to evaluate the activity profile of the drug.The initial dose level was 60 mg/m$^2$/week and the dose was increased by a 20 mg/m$^2$ stepwise increment in subsequent cohorts of 6 pts each. Once the MTD was reached, 6 more pts had to be included at the previous level in order to confirm the RD. I.V. VRL having demonstrated to be highly effective in ABC, this Phase I study was performed in this indication. Results are available on 27 pts (mean age : 55 y.o. ; range : 33-77): 7 at 60 mg/m$^2$, 14 at 80 mg/m$^2$ and 6 at 100 mg/m$^2$. Pts were pretreated by previous adjuvant chemotherapy for 6 of them and for advanced/metastatic disease for 19 of them. About 50 % had predominantly visceral disease (9 liver, 8 lung). 9 pts had bone metastasis and 16 presented locally advanced/metastatic disease. 100 mg/m$^2$/week was shown to be the MTD : among the 6 pts included, 3 experienced G 4 neutropenia, 2 G 3/4 constipation and 2 G 3 vomiting. Consequently, 80 mg/m$^2$/week was defined as the RD : G 3-4 neutropenia occurred following 21.4 % of cycles, but without any clinical consequences ; there were no G 3 nausea and G 3/4 vomiting occured following 1.4 % of cycles ; G 3/4 neuroconstipation occurred following 1 % of cycles. No G > 2 anemia or thrombocytopenia was observed at any dose level and only 1 pt experienced G 3 alopecia. Efficacy results were very encouraging : over 13 evaluable pts treated at 80 mg/m$^2$/week and, for 2 of them, at 100 mg/m$^2$ for 2 courses and then at 80 mg/m$^2$/week, 6 partial responses were observed (ORR = 46 % - IC95 : 19.1-73.3), 3 of them on visceral disease (2 lung, 1 liver). The median duration of response was $30^+$ weeks ($20^+$-$60^+$). It is concluded that oral VRL administered at the weekly dose of 80 mg/m$^2$ is well tolerated by the patients and presents a very interesting activity in the treatment of ABC.

### P108 Pattern of expression of MHC antigens and epidermal growth factor receptor (EGFr) in oral cancer :

A M E Nouri, H Cannell* and Oliver RTD. Depts. of Medical Oncology and of Oral & Maxillofacial Surgery*, London Hospital

Whilst the profiles of major histocompatibility complex (MHC) antigen expression for malignancies of bladder, oesophagus, breast, cervix and prostate have been published, less is known concerning their expression in oral squamous cell carcinoma (OSCC). Using monoclonal antibodies (Mabs) and immunocytochemical staining, the pattern of expression of MHC class I (monomorphic and polymorphic), class II antigens, pan-T cell marker, EGFr and adhesion molecule (ICAM-1), was investigated on OSCC and the results compared with those from ameloblastoma and from bladder tumours.

Results showed:- compared with benign tumour and with normal controls:-

a) $\beta$2-macroglobulin and Class I antigens were expressed in most cases of oral SCC.

b) both Class II and ICAM-1 (cell adhesion molecule) were expressed in most cases of oral OSCC.

c) T cell infiltration was also found to be at a higher density in oral OSCC.

d) strong EGFr expression was present in most OSCC.

These findings are consistent with the notion that there were abnormalities in the expression of MHC antigens in oral cancers. Our working hypothesis to explain the unexpected higher frequency of these abnormalities in the benign ameloblastoma is that although both tumour types (more so in the case of ameloblastoma) have in place an escape mechanism from the immune system, the over expression of EGFr in OSCC may in part explain the more aggressive behaviour of the malignancy.

P109

### THE UTILITY OF A CANCER INFORMATION SERVICE ON THE INTERNET

GG Dark*. CancerWEB, Gray Laboratory Cancer Research Trust, Northwood, Middlesex, HA6 2JR, UK.

CancerWEB was established as a cancer information resource site on the World-Wide Web (WWW) in 1995. The aims were to provide up-to-date information about cancer for patients, clinicians and scientific researchers. An agreement was made with the National Cancer Institute to redistribute the Physicians Data Query (PDQ) database of information and the CancerLIT abstracts. These plain text files were processed into the formatting language HTML (HyperText Mark-up Language) for availability on the WWW. Further functionality was provided by the addition of a powerful search and indexing program which allows users to locate files or documents that contain the specified search terms. The site has had over 2 million requests from over 65 countries and has transferred in excess of 15 Gigabytes of information in the first 12 months.

The provision of information on the Internet allows patients and clinicians to access the documents at a time and setting to suit their needs. However, this flexibility also extends to learning modules or multimedia teaching files, that allow users to learn new topics at their own learning rates.

Random users were prompted for feedback on their views of the service, regarding; the ease of access to information compared to traditional sources, the relevance to their enquiry, the usefulness of the service and whether they would return to use the service again.

Of the 1000 users questioned, 69% replied. The majority felt that the Internet gave far greater ease of access to information than traditional sources, 93% of users had their enquiries answered by the content of the documents, but many wanted additional more detailed information. The organisation of resources into sections for groups of users, such as clinicians and patients, was felt to be the most useful structuring of the resources and 95% of responders indicated satisfaction with the service and would be returning.

The resources available on the Internet for patients with cancer and clinicians working in oncology are increasing every day. This media certainly has an exciting future with an increasing amount of information becoming available to a desktop computer in the home, laboratory or outpatient clinic.

url: *http://www.graylab.ac.uk/cancerweb.html*

mail: *cancerweb@www.graylab.ac.uk*

P110

### EVALUATION OF CANCERHELP UK, INTERNET INFORMATION PROVIDER.

*S.J.Tweddle[1], B. Wake[2], D. Davies[3], C. James[1], P. Riding[4], D. Coats[5], N. James[1]. 1 CRC Institute for Cancer Studies, Clinical Research Block, Edgbaston, Birmingham B15 2TT. 2 National Council for Educational Technology, Milburn Hill Road, Science Park, Coventry CV7 2JJ, 3 Medical School, University of Birmingham, Edgbaston, Birmingham B15 2TT, 4 Webserve, PO Box 56, Saffron Walden, Essex CB11 4HT, 5 BACUP, 3 Bath Place, Rivington Street, London EC2A 3JR

There is a proliferation of cancer information services on the World Wide Web emanating from a variety of sources and an increasing demand from British Internet users for the information they provide. The evaluation of such services is essential given that they will become more widely available in the future. We have developed a British-based cancer information service for the general public (CancerHelp UK) in collaboration with BACUP which we have evaluated through 2 pilot studies as a preliminary to a multi-site randomised cross-over study. The first study, of usage in hospital waiting rooms by 60 patients and staff, evaluated the useability of a pilot service. The second study evaluated the usefulness of a more fully developed prototype service over a four month period on the Internet through analysis of electronic feedback and computer usage data. Of the patients and staff who used the service in waiting rooms, 82% were women; all patients were over 40 and 94% of all users said they would use the service again, although none had prior Internet experience and only 9% had 'a lot' of prior computer experience. 3.1% of Internet users returned feedback forms of whom 52% were male, 47% female and 1% unspecified. 36% were friends and relatives, 25% patients, 22% healthcare professionals and the remainder 'other'.Thematic analysis of coded responses indicated that information of most interest was that about specific cancers, treatment and research into new treatments. Data collected on the computer server showed that information on specific cancers and treatment was most frequently accessed. 83% of users appeared to be serious users, as opposed to 'surfers', accessing 5 or more pages of the service. The CancerHelp UK service appears to be accessible and highly valued as a provider of information about cancer and its treatment. A study currently in progress and funded by the Cancer Research Campaign will indicate for which sectors of the population CancerHelp UK proves most beneficial and how the service can be further improved.